# Antioxidant, Anti-Inflammatory, and Antidiabetic Activities of Bioactive Compounds from the Fruits of *Livistona chinensis* Based on Network Pharmacology Prediction

**DOI:** 10.1155/2021/7807046

**Published:** 2021-10-18

**Authors:** Yuwei Wang, Jianxiu Zhai, Dan Yang, Na Han, Zhe Liu, Zhihui Liu, Sikai Li, Jun Yin

**Affiliations:** Department of Pharmacognosy and Utilization Key Laboratory of Northeast Plant Materials, School of Traditional Chinese Medicine, Shenyang Pharmaceutical University, Shenyang 110016, China

## Abstract

In this study, a chemical investigation on the fruits of *Livistona chinensis* (FLC) led to the isolation and identification of 45 polyphenols and 5 alkaloids, including two new compounds (Livischinol (1) and Livischinine A (46)), an undescribed compound (47) and 47 known compounds. FLC was predicted with novel potential antidiabetic function by collecting and analyzing the potential targets of the ingredients. Compound 32 exhibited significant *α*-glucosidase inhibitory activity (IC_50_ = 5.71 *μ*M) and 1, 6, and 44 showed the PTP1B inhibitory activity with IC_50_ values of 9.41-22.19 *μ*M, while that of oleanolic acid was 28.58 *μ*M. The competitive inhibitors of PTP1B (compounds 1 and 44) formed strong binding affinity, with catalytic active sites, proved by kinetic analysis, fluorescence spectra measurements, and computational simulations, and stimulated glucose uptake in the insulin-resistant HepG2 cells at the dose of 50 *μ*M. In addition, FLC was rich in antioxidant and anti-inflammatory bioactive compounds so that they could be developed as nutraceuticals against diabetes.

## 1. Introduction

*Livistona chinensis* is a common ornamental plant widely distributed in eastern Asia. Studies on the seeds (containing flavonoids) [1], leaves (containing flavonoids) [2], and roots (containing phenols, ceramides and glycerides) [3, 4] of *Livistona chinensis* have demonstrated several biological activities, such as anticancer [1, 4], antiangiogenesis [5, 6], cardioprotective effect [2], antioxidant [2, 3], and antiosteoporosis [3]. The fruits of *Livistona chinensis* (FLC) were an edible functional food used for soup cooking [7]. It is also used to cook soups with pork to alleviate chronic hepatitis and liver cancer in folk, especially in eastern Asia [7, 8]. FLC was found to be enriched in natural polyphenolics consisting of flavonoids, phenols, lignans, and anthraquinones [8]. Pharmacological studies showed that FLC possessed antioxidant, antitumor, and hepatoprotective effects [7, 9–17]. Due to the huge production of FLC and diverse biological activities of the polyphenols in FLC, their potential edibleness needs to be exploited. FLC might possess a new antidiabetic function by systematic phytochemical investigations and network pharmacology analysis according to our study.

Type 2 diabetes is a complex metabolic disorder associated with developing insulin resistance, impaired increased oxidative stress, inflammation, insulin signaling, abnormal glucose metabolism, and so on. The disorder leads to a consequent decrease in quality of life and an increase in the rate of mortality [18]. At present, *α*-glucosidase inhibitors have been used against type 2 diabetes in clinic, such as acarbose and miglitol [19–23]. However, the above drugs could cause some side effects such as diarrhea, flatulence, and acute hepatitis. Protein tyrosine phosphatase 1B (PTP1B), another key enzyme related to type 2 diabetes, works as a negative governor for the insulin signaling pathway by dephosphorylating both the insulin receptor and the downstream insulin receptor substrate proteins [24, 25]. Consequently, new *α*-glucosidase inhibitors and PTP1B inhibitors are promising therapeutic agents to treat type 2 diabetes. Moreover, a large number of investigations have suggested that a diet rich in foods with antioxidant polyphenols is related to a lower risk of diabetes and predisposing factors [26].

To develop the potential edibleness of FLC, systematic phytochemical investigations were undertaken in this study. Then, FLC was predicted with novel antidiabetic function utilizing chemical profile and network pharmacology. The network pharmacology analysis also revealed that the biological process of FLC against diabetes might be involved in the regulation of inflammatory response, oxidation-reduction process, glucose metabolic process, insulin signaling pathway, and so on. Therefore, the extract, the fractions, and all isolated compounds were assayed for their antioxidant, anti-inflammatory, and inhibitory effect on *α*-glucosidase and PTP1B. The PTP1B inhibitory mechanism and effect on glucose uptake in insulin-resistant HepG2 cells of FLC-derived polyphenolic PTP1B inhibitors were further explored.

## 2. Materials and Methods

### 2.1. Plant Material

The fruits of *Livistona chinensis* R. Brown were collected in July 2017 from Xinhui, Guangdong Province, China, and were identified by Jun Yin, the professor of Shenyang Pharmaceutical University, and were deposited in the laboratory of the Department of Pharmacognosy. A voucher specimen (FLC-20170710) of this crude drug was deposited in the herbarium of the Department of Natural Products Chemistry, Shenyang Pharmaceutical University.

### 2.2. Chemicals and Reagents

Column chromatography was performed on silica gel (100-200 mesh and 200-300 mesh, Qingdao Marine Chemical Group, Co., Qingdao, China), ODS (50-100 mesh, YMC, Co., Ltd., Japan), polyamide (100-200 mesh, Sinopharm Chemical Reagent Co., Ltd.), and Sephadex LH-20 (Amersham Pharmacia Biotech AB Co.). Semipreparative HPLC was conducted on a Shimadzu LC-10A instrument with an LC-10ATVP pump, a Shimadzu LC-10AVPUV-VIS detector (Shimadzu Co., Ltd.), and an N-2000 chromatographic work station (Intelligent Information Engineering Co., Ltd.) using Welch 5 *μ*m C18 column (250 × 10 mm). Analytical HPLC was carried out on an Agilent 1260 HPLC system using a Welch 5 *μ*m C18 column (250 × 4.6 mm). *α*-Glucosidase (from Baker's yeast) and indomethacin were purchased from Shanghaiyuanye Bio-Technology Co., Ltd. Recombinant human PTP1B protein (ab51277) was purchased from Abcam (Shanghai) Trading Co., Ltd. All chemical reagents used in the studies were produced by Laibo Chemicals Industries, Ltd.

### 2.3. Extraction and Isolation

The whole dry fruits (40 kg) of *Livistona chinensis* R. Brown were extracted with 70% ethanol by heat reflux extraction (3 × 320 L, 2 h each time). The ethanol extract was suspended in water and successively partitioned with petroleum ether, dichloromethane, ethyl acetate, and butyl alcohol, respectively, to obtain petroleum ether extract (8 g), dichloromethane extract (26 g), ethyl acetate extract (55 g), and butyl alcohol extract (420 g). The petroleum ether extract was not further fractioned, since fatty acids are primary constituents in it [8]. The dichloromethane layer (22 g) was chromatographed on a silica gel column (100-200 mesh) with petroleum ether/acetone (50 : 1-1 : 2, *v*/*v*) to yield 6 fractions (Fr.A1-Fr.A6). Compound 45 (370 mg) was recrystallized from Fr.A2. Fr.A3 was further separated with Sephadex LH-20 (MeOH) and semipreparative HPLC (MeOH-H_2_O, 65 : 35, *v*/*v*) to obtain compounds 43 (2.6 mg) and 48 (3.0 mg). Fr.A4 was subjected to Sephadex LH-20 (MeOH) to yield 5 subfractions (Fr.A4.1-Fr.A4.6) according to their TLC profiles. Fr.A4.2 was further purified by semipreparative HPLC (MeOH-H_2_O, 50 : 50, *v*/*v*) to obtain compound 27 (3.2 mg). Fr.A4.3 was further purified using semipreparative HPLC (MeOH-H_2_O, 60 : 40, *v*/*v*) to afford compounds 28 (5.0 mg), 29 (4.1 mg), and 30 (3.2 mg). Compounds 13 (4.2 mg) and 31 (2.6 mg) were purified from Fr.A4.4 by semipreparative HPLC (MeOH : H_2_O, 45 : 55, *v*/*v*). Fr.A4.5 was further purified by semipreparative HPLC (MeOH : H_2_O, 35 : 65, *v*/*v*) to obtain compounds 14 (2.4 mg), 32 (2.0 mg), 33 (2.0 mg), 34 (1.8 mg), 35 (1.9 mg), and 36 (2.3 mg). Fr.A4.6 was further purified using semipreparative HPLC (MeOH-H_2_O, 65 : 35, *v*/*v*) to obtain compounds 19 (2.9 mg), 20 (2.5 mg), 21 (3.8 mg), 25 (2.7 mg), and 26 (2.3 mg).

The ethyl acetate layer (50 g) was chromatographed on a silica gel column (100-200 mesh) with dichloromethane/methanol (100 : 1-1 : 1, *v*/*v*) to yield 6 fractions (Fr.B1-Fr.B6) according to their TLC profiles. Fr.B2 was fractionated by silica gel column chromatography with petroleum ether/ethyl acetate (30 : 1-1 : 1, *v*/*v*) to produce 5 subfractions (Fr.B2.1-Fr.B2.5). Fr.B2.2 was further purified using semipreparative HPLC (MeOH : H_2_O, 60 : 40, *v*/*v*) to obtain compounds 41 (5.0 mg) and 42 (8.0 mg). Fr.B2.3 was further fractionated by silica gel column chromatography with petroleum ether/acetone (12 : 1, *v*/*v*) to produce compound 40 (4.0 mg). Fr.B2.4 was further fractionated by silica gel column chromatography with petroleum ether/acetone (8 : 1, *v*/*v*) to produce compound 50 (5.0 mg). Compound 37 (49.0 mg) was recrystallized from Fr.B3. Fr.B4 was further separated by a reversed-phase ODS (MeOH : H_2_O, 20 : 80-60 : 40, *v*/*v*) to produce 3 subfractions (Fr.B4.1-Fr.B4.3), and compound 8 (25 mg) was recrystallized from Fr.B4.2. Fr.B5 was chromatographed on a reversed-phase ODS (MeOH : H_2_O, 20 : 80-60 : 40, *v*/*v*) to obtain 5 subfractions (Fr.B5.1-Fr.B5.5). Fr.B5.1 was fractionated by silica gel column chromatography with petroleum ether/ethyl acetate (10 : 1-2 : 1, *v*/*v*) to yield compounds 38 (280 mg) and 39 (3.5 mg). Fr.B5.2 was separated by polyamide (100-200 mesh) column chromatography (MeOH : H_2_O, 10 : 90-30 : 70, *v*/*v*) to get 3 subfractions (Fr.B5.2.1-Fr.B5.2.3). Fr.B5.2.2 was further purified by semipreparative HPLC (MeOH : H_2_O, 45 : 55, *v*/*v*) to obtain compound 15 (4.0 mg). Fr.B5.3 was separated by polyamide (100-200 mesh) column chromatography (MeOH : H_2_O, 30 : 70-50 : 50, *v*/*v*) to get 3 subfractions (Fr.B5.3.1-Fr.B5.3.3). Fr.B5.3.1 was further purified by semipreparative HPLC (MeOH : H_2_O, 45 : 55, *v*/*v*) to obtain compound 12 (5.0 mg), compound 7 (3.0 mg) was purified by semipreparative HPLC (MeOH-H_2_O, 50 : 50, *v*/*v*) from Fr.B5.3.2, and compound 11 (9.8 mg) was recrystallized from Fr.B5.3.3.

The butyl alcohol layer (200 g) chromatographed on a silica gel column (100-200 mesh) with dichloromethane/methanol (50 : 1-1 : 1, *v*/*v*) to yield 6 fractions (Fr.C1-Fr.C6). Fr.C2 was fractionated by silica gel column chromatography with petroleum ether/ethyl acetate (15 : 1, *v*/*v*) to get compound 6 (12 mg). Fr.C4 was subjected to a reversed-phase ODS by elution (MeOH : H_2_O, 10 : 90-70 : 30, *v*/*v*) to obtain 5 subfractions (Fr.C4.1-Fr.C4.5). Fr.C4.2 was further separated with Sephadex LH-20 (MeOH) to get compound 44 (4.2 mg). Fr.C4.3 was separated by polyamide (100-200 mesh) column chromatography (MeOH : H_2_O, 10 : 90-30 : 70, *v*/*v*) to get 4 subfractions (Fr.C4.3.1-Fr.C4.3.4). Fr.C4.3.1 was further purified by semipreparative HPLC (MeOH-H_2_O, 35 : 65, *v*/*v*) to obtain compounds 46 (7.0 mg) and 47 (4.0 mg). Fr.C4.3.2 was further purified by semipreparative HPLC (MeOH-H_2_O, 35 : 65, *v*/*v*) to obtain compounds 46 (7.0 mg) and 47 (4.0 mg). Fr.C4.3.3 was further purified using semipreparative HPLC (MeOH-H_2_O, 32 : 68, *v*/*v*) to obtain compounds 1 (3.0 mg), 2 (14.0 mg), 3 (96.0 mg), and 4 (72.0 mg). Compound 5 was obtained from Fr.C4.3.4 by semipreparative HPLC (MeOH : H_2_O, 30 : 70, *v*/*v*). Fr.C4.4 was further separated with Sephadex LH-20 (MeOH) to get compound 9 (26.0 mg), and the subfraction was purified using semipreparative HPLC (MeOH-H_2_O, 38 : 62, *v*/*v*) to obtain compounds 17 (3.7 mg) and 18 (5.5 mg). Compound 10 (152 mg) was recrystallized from Fr.C4.5. Fr.C5 was chromatographed on a reversed-phase ODS (MeOH : H_2_O, 15 : 85-25 : 75, *v*/*v*) to obtain 3 subfractions (Fr.C5.1-Fr.C5.3). Compound 49 (4.6 mg) was isolated from Fr.C5.1 by semipreparative HPLC (MeOH-H_2_O, 65 : 35, v/v). Further purification of Fr.C5.2 by Sephadex LH-20 (MeOH) and semipreparative HPLC (MeOH-H_2_O, 35 : 65, *v*/*v*), yielded compounds 16 (3.6 mg), 22 (3.2 mg), 23 (3.3 mg), and 24 (4.0 mg).

Livischinol (1): orange amorphous powder;  [*α*]_D_^20^ -23 (*c* 0.22, MeOH); UV (MeOH) *λ*_max_ (log *ε*) 210 (1.37), 283 (0.189) nm; IR (KBr) *v*_max_ 3392, 1600, 1626, 1464, 1384, 1025 cm^−1^; ^1^H and ^13^C NMR data, Table 1; HRESIMS *m*/*z* 359.1131 ([M-H]^−^, calculated for C_19_H_19_O_7_, 359.1131).

Livischinine A (46): light yellow amorphous powder; [*α*]_D_^20^ was -33 (c 0.23, MeOH); UV (MeOH) *λ*_max_ (log *ε*) 213 (2.13), 276 (1.48) nm; IR (KBr) *v*_max_ 3333, 1712, 1641, 1517, 1457, 1412 cm^−1^; ^1^H and ^13^C NMR data, Table 1; HRESIMS *m*/*z* 236.1137 ([M + H]^+^, calculated for C_10_H_14_N_5_O_2_, 236.1147).

8-hydroxy-zeatin (47): light yellow amorphous powder; UV (MeOH) *λ*_max_ (log *ε*) 213 (2.93), 275 (2.06) nm; IR (KBr) *v*_max_ 3393, 1706, 1640, 1515, 1464, 1412 cm^−1^; ^1^H and ^13^C NMR data, Table 1; HRESIMS *m*/*z* 236.1136 ([M + H]^+^, calculated for C_10_H_14_N_5_O_2_, 236.1147).

### 2.4. Network Pharmacology Analysis

#### 2.4.1. Chemical Ingredient Database Building

The information of compounds was selected from our laboratory and references. Two-dimensional structures and the SMILES strings of the compounds were sketched by ChemBioDraw19.0.

#### 2.4.2. Target Fishing

The candidate targets of the ingredients from FLC were collected from PubChem (http://pubchem.ncbi.nlm.nih.gov), SwissTargetPrediction (http://www.swisstargetprediction.ch/), and SuperPred (http://prediction.charite.de/index.php?site=chemdoodle_search_target) database and references. The targets related to diabetes were obtained using Comparative Toxicogenomic Database (http://ctdbase.org/), Therapeutic Target Database (http://bidd.nus.edu.sg/group/cjttd/), and DrugBank (http://www.drugbank.ca/) database. The targets were normalized in UniPort (http://www.uniport.org/) [27].

#### 2.4.3. Network Construction and Analysis

The related diseases were predicted by the targets of the ingredients. And the disease enrichment analysis was obtained by DAVID database (http://david.ncifcrf.gov/). The overlap targets were the candidate targets of FLC against diabetes. To cluster the biological functions and clarify the pathways that were involved in putative drug targets, the analysis of GO function and KEGG (http://www.genome.jp/kegg/mapper.htmL) signaling pathways was performed using the clusterProfiler and ggplot2 packages in R software (version.3.3.0). GO enrichment analysis mainly consisted of biological processes. Besides, the enrichment analysis was also obtained by DAVID database. *P* ≤ 0.05, as the cutoff value, was calculated by the two-sided hypergeometric test method to identify enriched GO terms and the localization of the biological and molecular functions of the proteins, which indicated the relative importance of enriched GO terms and pathways. The ingredient-target network was established by Cytoscape.3.4.0 software [27].

### 2.5. Antioxidant Activities

#### 2.5.1. DPPH Assay

The DPPH free radical scavenging assay was performed according to a previously reported method [28]. A series of different concentrations of the tested samples and the positive control ascorbic acid were mixed with freshly prepared DPPH (0.2 mM) in a 96-well microplate with a total volume of 150 *μ*L. After leaving the mixture to react in the dark for 30 min, its absorbance was determined at 517 nm. All of the wells with only samples were set as blanks to obtain an absorbance value, which was subtracted from the test sample readings. The percentage inhibition (%) for each sample was calculated by the following formula:

(1) $$\begin{array}{c} \text{Inhibition }\left(\%\right)=\left(\frac{{\text{Abs}}_{\text{blank}}-{\text{Abs}}_{\text{samples}}}{{\text{Abs}}_{\text{blank}}}\right)\times 100\%, \end{array}$$

where Abs_blank_ and Abs_samples_ correspond to the absorbance units (at 517 nm) of DPPH solutions incubated in the absence and presence of the tested samples.

#### 2.5.2. ABTS Assay

The ABTS radical cation (ABTS·+) was prepared by reacting ABTS with potassium persulfate following the method previously described [28]. The ABTS radical cation (ABTS·+) was prepared by mixing with 2.45 mM potassium persulfate and equal volumes of an ABTS solution (7 mM), and then, they were permitted to react in the darkness at room temperature for 12–16 h. Thereafter, the ABTS reagent was diluted with methyl alcohol until reaching an absorbance of 0.70 ± 0.02 units at 734 nm. In a 96-well microplate, ABTS reagent (190 *μ*L in each well) and samples (10 *μ*L in each well) were incubated at room temperature for 20 min. To determine the antioxidant capacity by the ABTS method, the experimental conditions and protocol were identical to those of the DPPH assay. The only difference was that the absorbance was measured at 734 nm.

### 2.6. Anti-Inflammatory Assay

The anti-inflammatory activity was estimated using lipopolysaccharide- (LPS-) induced RAW264.7 cells as reported [29].

#### 2.6.1. MTT Assay for Cell Viability

RAW 264.7 cells were plated into a 96-well plate (2 × 10^4^ cells/well) and cultured in RPMI-1640 with 10% FBS at 37°C under 5% CO_2_. After incubation for 12 h, the cells were pretreated with the samples for 4 h, which were then stimulated with LPS (1 *μ*g/mL) for 20 h. And the control group was without the samples. Besides, 0.5 mg/mL MTT was then added to plates, which were incubated for further 4 h. Subsequently, 100 *μ*L dimethyl sulfoxide (DMSO) was then added to each well to dissolve the crystals. The absorbance was recorded at a wavelength of 490 nm by a BioTek microplate reader.

#### 2.6.2. Nitrite Assay

The level of NO production was tested by measuring the nitrite level in the culture medium with the Griess method [29]. Cells were seeded at a density of 2 × 10^4^ cells per well in 96-well plates. After the cells were pretreated with indomethacin and the test samples for 4 h, LPS (1 *μ*g/mL) was added to the medium for further incubation. 20 h later, nitrite production was measured by mixing 100 *μ*L of supernatant and 100 *μ*L of Griess reagent in a 96-well plate for 10 mins. A microplate reader was employed for the optical density measurement at 570 nm. The percentage inhibition of NO was determined as follows:

(2) $$\begin{array}{c} \text{Inhibition }\left(\%\right)=\left(\frac{A_{c}–A_{s}}{A_{c}\;}\right)\times 100\%, \end{array}$$

where *A*_*c*_ is the absorbance of the control and *A*_*s*_ is the absorbance of the test sample.

### 2.7. *α*-Glucosidase Inhibitory Assay

According to the previously published method [28], the *α*-glucosidase inhibition was assayed using *p-*nitrophenyl-*α*-D-glucopyranoside (*p*-NPG) as the substrate, the release of *p*-nitrophenol was measured with a microplate reader at 405 nm. Acarbose was used as the positive control. The extract, fractions, and compounds were dissolved in 5% DMSO and then diluted with phosphate buffer. Briefly, 80 *μ*L PBS, 20 *μ*L of varying concentrations of sample solutions, and 20 *μ*L *α*-glucosidase solution (1.3 U/mL) were mixed and incubated in 96-well plates at 37°C for 5 min. The reaction started when 20 *μ*L *p*-nitrophenyl-*α*-D-glucopyranoside (2.5 mM) was added to the plate. The reaction mixture was incubated for 15 min at 37°C, and then, 80 *μ*L Na_2_CO_3_ (0.2 M) was added to stop the reaction. Their inhibitory effects were measured with a microplate reader at 405 nm. The percentage inhibition (%) for each sample was calculated as follows:

(3) $$\begin{array}{c} \text{Inhibition }\left(\%\right)=\left(1-\frac{A_{a}–A_{b}}{A_{c}–A_{d}\;}\right)\times 100\%, \end{array}$$

where *A*_*a*_ represents the absorbance of the sample group with the enzyme, *A*_*b*_ represents the absorbance of the sample control group without enzyme, *A*_*c*_ represents the absorbance of the control group without samples, and *A*_*d*_ represents the absorbance of the blank control group without samples and enzyme.

### 2.8. Methodologies for PTP1B Studies

#### 2.8.1. PTP1B Inhibitory Assay

The PTP1B inhibitory activity was determined by measuring the rate of hydrolysis of a substrate, disodium 4-nitrophenylphosphate (*p*-NPP), and oleanolic acid was used as a positive control, according to the previously published protocols [30]. PTP1B (10 *μ*g/mL stock solution) was dissolved in 10 mM citrate buffer (pH 6.0), 1 mM dithiothreitol (DTT), and 1 mM N, N, N′, N′-ethylenediaminetetraacetate (EDTA). 20.0 *μ*L PTP1B stock solution and 60 *μ*L citrate buffer were added to each well of a 96-well plastic plate. Each sample (20.0 *μ*L in DMSO) was added to each well to make a final concentration and incubated at 37°C. The series final concentrations of oleanolic acid were 6.25, 12.5, 25, 50, and 100 *μ*M. The reaction was initiated by the addition of *p*-NPP (20 *μ*L of 12 mM stock solution) in the citrate buffer, incubated at 37°C for 30 min, and terminated with the addition of 80 *μ*L of a stop solution (2.5 M NaOH). The optical density of each well was measured at 405 nm using an MTP-500 microplate reader. The PTP1B inhibitory activity was calculated by formula (2).

#### 2.8.2. Enzyme Inhibition Kinetic Study

Lineweaver−Burk double reciprocal plot and Dixon plot were used to study the kinetic behavior of active compounds against PTP1B and the corresponding inhibition constants (*K*_*i*_ values). Enzyme reactions were conducted at various concentrations of *p*-NPP substrate (0.3, 0.6, and 1.2 mM) with active compounds at different concentrations (0, 3.125, 6.25, and 12.5 *μ*M for compound 1, 0, 6.25, 12.5, and 25 *μ*M for compounds 44 and 6) and 100 nM PTP1B in 96-well plates. The plate reader recorded the absorbance of the reaction mixture every 3 mins. The enzymatic velocity of the enzyme reaction was calculated based on the time − Δabs plot [31]. The Lineweaver−Burk plot and Dixon plot were generated by GraphPad Prism 8 in order to determine the type of PTP1B inhibition and *K*_*i*_.

#### 2.8.3. Fluorescence Measurement

180 *μ*L of the buffer with 10 *μ*L of the PTP1B at the concentration used in the assays were added into the 96-well black plates followed by 10 *μ*L different concentrations (0, 6.25, 9.37, 12.5, 18.75, 25, and 50 *μ*M) of inhibitor. The excitation wavelength was set at 250 nm, and the fluorescence emission spectra were collected from 300-400 nm with an emission bandwidth of 2 nm [32]. The quenching parameters such as the Stern-Volmer constant (*K*_SV_), binding constant (*K*_A_), and the number of binding sites (*n*) were determined by the formula [33].

(4) $$\begin{array}{c} \frac{F_{0}}{F}=1+K_{\text{SV}}{\left[Q\right]}_{f},\\ \log\frac{Fo-F}{F}=\log K_{\text{A}}+n\log{\left[Q\right]}_{f}, \end{array}$$

where *F*_0_ and  *F* are the fluorescence intensities in the absence and presence of a quencher and [*Q*]_*f*_ is a concentration of compounds.

#### 2.8.4. Molecular Docking Analysis

Molecular docking can analyze the ability of a compound to bind to a target protein and predict the physiological activity of a candidate compound *in silico*. The docking protocol was conducted with the AutoDock Vina (version. 4.2.6) following the method previously described with modifications [34]. First, ChemBioDraw 3D was used to prepare 3D chemical structural formulas and energy minimizing for all the compounds and then saved results in MOL.2 format. Then, the crystal structures of candidate targets were downloaded from RCSB Protein Data Bank (http://www.rcsb.org/). Finally, they were decorated through Python (version. 2.5) and AutoDock Vina (version. 4.2.6), including removing the ligands, adding hydrogen, removing water, and optimizing and patching amino acids. The 2D structures of receptor-ligand interactions were performed by Discovery Studio 2020 Client software.

#### 2.8.5. Molecular Dynamics Simulations

Molecular dynamics simulations were carried out of PTP1B apoprotein- and PTP1B-compound complexes, respectively, following the method previously described with modifications [35]. In order to obtain a stable and low-energy protein conformation, the mode was optimized by using the Desmond (v3.8) module in the Schrödinger software for molecular dynamics simulation with optimized potentials for liquid simulations (OPLS) all-atom force field 2005. 50 ns molecular dynamics simulations were carried out of PTP1B apoprotein- and PTP1B-compound complexes, respectively. The system was solvated with simple point charge (SPC) water and neutralized by adding an appropriate amount of counterions in a 10 Å×10 Å×10 Å orthorhombic box, so as to form a buffer region between protein atoms and box sides. Additionally, the OPLS_2005 force field was used to minimize the energy of the complex system, and the maximum iterations during minimization were set to 5000, and the convergence threshold was kept at 1.0 kcal/mol/Å. Hybrid methods of the steepest descent and the limited memory Broyden Fletcher Goldfarb Shanno (LBFGS) algorithms with a maximum of 5000 steps were performed to minimize the system energy until a gradient threshold of 25 kcal/mol/Å was reached. Before molecular dynamics simulations, a 10 ns simulation was performed to relax the whole system, applying a normal pressure temperature (NPT) ensemble with a Nose-Hoover thermostat at 300 K and Martyna-Tobias-Klein barostat at 1.01325 bar pressure. At last, the molecular dynamics simulations were carried out Accepted Manuscript for 50 ns under the pressure at 1.01325 bar and the temperature at 300 K. The energy and trajectory atomic coordinate data were recorded at every 1.2 ps and 100 ps, respectively, and the obtained data were used for statistical analysis. Besides, root means square deviation (RMSD) of the protein-ligand complex was monitored during the whole simulation, and the obtained protein-ligand interaction histogram showed which amino acid residues interacted with the ligand and the proportion of each interaction.

### 2.9. Glucose Consumption by Insulin-Resistant HepG2 Cells

HepG2 cells were cultivated in DMEM (without phenol red) containing 10% fetal bovine serum at 37°C in a humidified 5% CO_2_ atmosphere. An insulin-resistant cell model was established in accordance with a previous method, with some modifications. Cells were seeded in a 96-well plate with 1 × 10^4^ cells per well. After incubation for 24 h, the medium was replaced with serum-free medium (without phenol red) containing 5 × 10^−6^ mol/L insulin and incubated for 24 h. The cell culture medium was then replaced with the samples at different concentrations. The compounds were first dissolved in DMSO and subsequently diluted with medium containing 1% FBS to achieve different concentrations. The final concentration of DMSO was no more than 0.1%. At the end of the 24 h incubation, glucose in the medium was measured using the glucose oxidase-peroxidase method kit (Nanjing Jiancheng Biological Co., Ltd.). The amount of glucose consumption was calculated as follows: (glucose of the DMEM − glucose concentration of each well) [36].

### 2.10. Statistical Analysis

All data were expressed as mean SD from at least three separate experiments and the level of significance was set at *P* < 0.05. Statistical analysis was performed using GraphPad Prism 8 for windows. The IC_50_ values were calculated by GraphPad Prism 8.

## 3. Results and Discussion

### 3.1. Structure Elucidation

**Figure 1:**
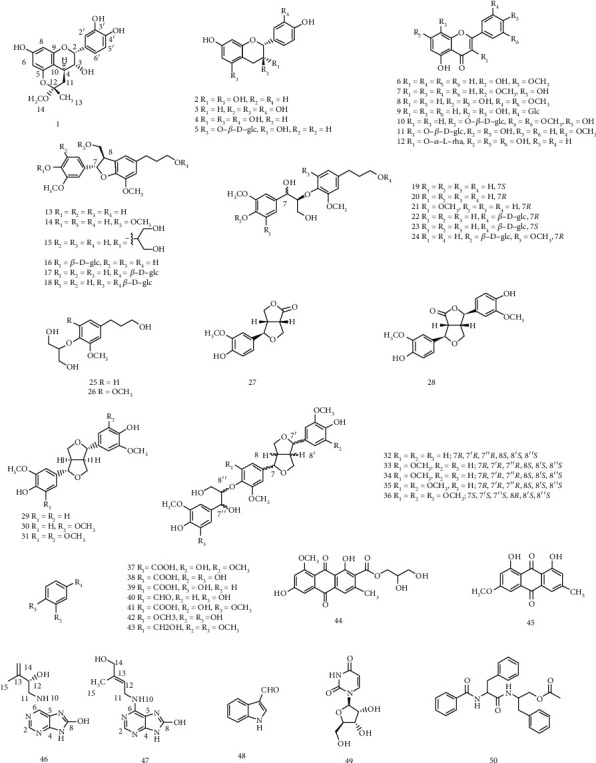
Chemical structures of compounds 1–50.

The chemical structures of the compounds isolated from FLC were shown in Figure 1.

**Figure 2:**
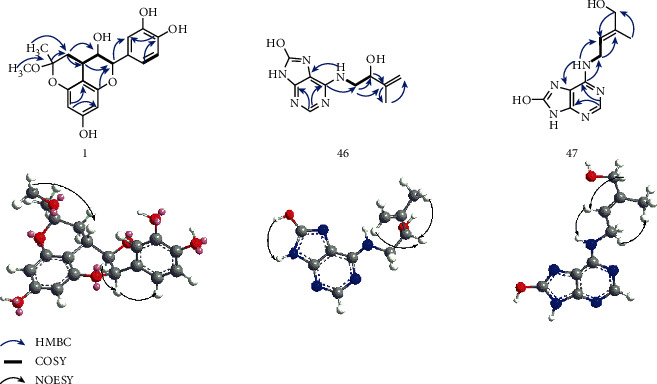
Key HMBC, ^1^H-^1^H COSY, and NOESY correlations for compounds 1, 46, and 47.

Compound 1 was obtained as an orange amorphous powder. Its molecular formula was determined to be C_19_H_20_O_7_ on the basis of its ^13^C NMR and HR-ESI-MS at *m*/*z* 359.1131 ([M-H]^−^, calculated for C_19_H_19_O_7_, 359.1131), suggesting 10 degrees of unsaturation. Its IR (KBr) spectrum showed absorptions of hydroxyl (3392 cm^−1^) and aromatics rings (1600, 1626 cm^−1^). In the ^1^H NMR (Table 1) spectrum, a pair of aromatic proton signals at *δ*_H_ 5.79 (d, *J* = 2.3 Hz) and *δ*_H_ 5.87 (d, *J* = 2.3 Hz) indicated the presence of two *meta*-coupling aromatic protons for a tetrasubstituted phenyl, and the aromatic proton signals at *δ*_H_ 6.69 (d, *J* = 2.1 Hz), *δ*_H_ 6.62 (d, *J* = 8.2 Hz), *δ*_H_ 6.51 (dd, *J* = 2.1, 8.2 Hz) suggested the presence of a 1, 3, 4-trisubstituted phenyl. The ^1^H NMR signals also displayed three methine protons at *δ*_H_ 5.12 (d, *J* = 5.1 Hz), *δ*_H_ 3.70 (dd, *J* = 11.1, 5.1 Hz), *δ*_H_ 2.54 (m), three methoxy protons at *δ*_H_ 3.17 (s), and three methyl protons at *δ*_H_ 1.45 (s). The ^13^C NMR (Table 1) and HSQC spectrum displayed 19 carbon resonances assignable to two methyls (one methoxy), one methylene, eight methines (five olefinic and two oxygenated), and eight quaternary carbons (seven olefinic). As shown in Figure 2, ^1^H-^1^H COSY data showed correlations between H-3/H-2, and H-2/H-4, indicating that the three methine protons were connected. The above data suggested that compound 1 was a flavanol derivative [37–40]. The ^1^H-^1^H COSY spectrum also displayed a correlation between H-4 and H-11, and the heteronuclear multiple-bond connectivity (HMBC) spectrum displayed correlations of H-4 (*δ*_H_ 2.54) with C-11 (*δ*_C_ 34.1), C-12 (*δ*_C_ 98.8) and H-11 (*δ*_H_ 1.29 and 2.13) with C-3 (*δ*_C_ 69.2), C-4 (*δ*_C_ 25.6). Therefore, a C-4-C-11 connection was confirmed. There were 10 degrees of unsaturation evident in the molecule of 1, of which nine were represented by flavanol and one unsaturation was left. The chemical shift of C-5 was shifted downfield by 5.4 ppm, compared with carbon atom in (-)-epicatechin [40], indicating that the ether was incorporated between C-5 and C-12 [37–39]. Moreover, the HMBC correlations of C-12 with H-11, H-13, and H-14, indicated that the methyl group and methoxy group were attached to C-12. Thus, the planar structure of compound 1 was showed as Figure 2.

The relative configuration of C-2 and C-3 in 1 was concluded to be of the epicatechin type from the characteristic feature of the H-2 resonance in the ^1^H-NMR spectrum: *δ*_H_ 5.12 (d, *J* = 5.1 Hz) [39, 41]. And the C-2 aryl substituent was suggested to be the equatorial orientation according to the thermodynamically favoured conformation existing in natural flavanones [42, 43]. The *J*_2, 3_ coupling constant of 5.1 Hz confirmed a 2, 3-*cis* configuration of 1, whereas the coupling constant (*J* = 11.1) between H-3 and H-4 indicated a 3, 4-*trans*-configuration. Due to the NOE correlation between H-4 and H-14, the relative configuration was determined and they were oriented to the same direction on the ring of inner ether. The CD spectrum showed a negative cotton effect at 240 nm, indicating the existence of 3*R* configuration [44, 45]. It was consistent with the CD data of 2,3-*cis*-3,4-*trans* (2*R*,3*R*,4*S*) flavanol [46]. On the basis of the above discussion and published same skeleton derivatives, the structure of 1 (shown in Figure 1) was finally assigned and named Livischinol.

Compound 46 was obtained as a light yellow amorphous powder, whose molecular formula was determined to be C_10_H_13_N_5_O_2_ based on its ^13^C NMR and HR-ESI-MS at *m*/*z* 236.1131 ([M + H]^+^, calculated for C_10_H_14_N_5_O_2_, 236.1147), suggesting 7 degrees of unsaturation. The IR (KBr) spectrum data at 3333, 1517, and 1712, 1641 cm^−1^, indicated the presence of amine groups and aromatics rings, respectively. The UV spectrum of 46 showed two absorption maxima at 213 and 276 nm. The ^1^H NMR (Table 1) signals displayed two geminal coupled olefinic protons (*δ*_H_ 4.97 (dt, *J* = 2.3, 1.1) and *δ*_H_ 4.82 (t, *J* = 2.3)), an oxymethines proton (*δ*_H_ 4.07 (dd, *J* =7.6, 4.2)), two methylene protons (*δ*_H_ 3.67 (ddd, *J* = 13.4, 6.4, 4.2) and *δ*_H_ 3.31 (m)), and three methyl protons (*δ*_H_ 1.72 (s)). The ^13^C NMR (Table 2) and HSQC spectrum displayed 10 carbon resonances assignable to one methyl, one methylene, seven olefinic carbons, and one oxygenated methine. The ^1^H and ^13^C NMR (Table 1) spectrums showed the same features as those of 8-hydroxyadenine [47]: one singlet at *δ*_H_ 8.02, three exchangeable protons at *δ*_H_ 11.22, 10.19, 6.55 and five aromatic carbon atoms at *δ*_C_ 104.73, 145.87, 147.37, 150.85, 152.90. Besides, the oxygen atom was attached to C-8 rather than C-2, which was finally deduced from the analysis of the HMBC spectroscopic data. Both methylene protons and the N_10_-H (*δ*_H_ 6.55) showed HMBC correlations with C-6 (*δ*_C_ 145.9) and a correlation a correlation between C-6 and *δ*_H_ 8.02; thus, the oxygen atom was present at C-8. Moreover, the NOE spectroscopy experiment showed a correlation between N_9_-H (*δ*_H_ 11.22) and O-H (*δ*_H_ 10.19), which strongly suggested the existence of this compound as an enol rather than an 8-keto tautomer. The ^1^H-^1^H COSY spectrum also displayed correlations of H-10 (*δ*_H_ 6.55) with H-11 (*δ*_H_ 3.67 and 3.31), H-11 with H-12 (*δ*_H_ 4.07) and the HMBC spectrum displayed correlations of H-11 with C-6 (*δ*_C_ 145.9), C-12 (*δ*_C_ 72.9). In addition, two methylene protons were at different chemical shifts due to the chiral carbon C-12. Thus, the connection of the branch was confirmed as shown in Figure 2, because  [*α*]_D_^20^ was -33 (c 0.23, MeOH), which was the same specific rotation with the known similar compound (*S*)-5-amino-pent-1-en-3-ol hydrochloride [48]. As reported, the specific rotation direction of the side-chain was the same with the side-chain attached to the purine ring [49, 50]. Thus, the absolute configuration of the chiral carbon C-12 was identified as *S*. The molecule was named livischinine A.

Compound 47 was obtained as a light yellow amorphous powder. Its molecular formula was determined to be C_10_H_13_N_5_O_2_ based on its ^13^C NMR and HR-ESI-MS at *m*/*z* 236.1131 ([M + H]^+^, calculated for C_10_H_14_N_5_O_2_, 236.1136), suggesting 7 degrees of unsaturation. Its IR (KBr) spectrum showed absorption bands at 3393, 1515, and 1706, 1640 cm^−1^, indicating the presence of amine groups and aromatics rings, respectively. The UV spectrum of 47 showed two absorption maxima at 213 and 275 nm. The ^1^H NMR (Table 1) signals indicated an olefinic proton at *δ*_H_ 5.50 (tp, *J* = 6.2, 1.5 Hz), four methylene protons at *δ*_H_ 4.03_(two)_ (t, *J* = 6.2 Hz) and *δ*_H_ 3.81_(two)_ (s), and three methyl protons at *δ*_H_ 1.63 (s). The ^13^C NMR data indicated signals for one methyl carbon (*δ*_C_ 14.1), one methylene carbon (*δ*_C_ 38.0), one methoxy carbons (*δ*_C_ 66.06), and seven *sp^2^* quaternary carbons. Compound 47 had the same 8-hydroxyadenine unit with compound 46, and the differences were the chemical shifts of the olefinic and two methylene protons. Based on the ^1^H-^1^H COSY correlations of H-12 (*δ*_H_ 5.50) with H-11 (*δ*_H_ 4.03), N_10_-H (*δ*_H_ 6.50), and the HMBC correlations of C-12 (*δ*_C_ 120.0) with H-11, N_10_-H, this indicated the methylene group was attached to the olefinic carbon. Besides, in the NOESY spectra, the correlations of H-12 (*δ*_H_ 5.50) and H-14 (*δ*_H_ 3.81) confirmed an *E* configuration. The spectroscopic data suggested the structure of this compound was the same with 8-hydroxy-zeatin [51]. The spectroscopic data was first described except for the mass spectra.

The 47 known compounds were identified as (-)-epiafzelechin (2) [52], (+)-catechin (3) [53], (-)-epicatechin (4) [40], (-)-epiafzelechin-5-*O*-*β*-_D_-glucoside (5) [54], wogonin (6) [55], genkwanin (7) [56], tricin (8) [57], vitexin (9) [58], tricin-7-O-*β*-_D_-glucoside (10) [59], isorhamnetin-3-*O*-*β*-_D_-glucoside (11) [60], astilbin (12) [61], (7*S*,8*R*)-dihydrodehydrodiconiferyl alcohol (13) [62], (7*S*,8*R*)-5-methoxydihydrodehydroconiferyl alcohol (14) [63], (7*S*,8*R*)-9,9′-dihydroxyl-3,3′-dimethoxyl-4-*O*-glycerol-7,8-dihydrobenzofuran-1′-propanolneoligan (15) [64], (7*S*,8*R*)-dihydrodehydrodiconiferyl alcohol-4-*O*-*β*-_D_-glucopyranoside (16) [65], (7*S*,8*R*)-dihydrodehydrodiconiferyl alcohol-9′-*O*-*β*-_D_-glucoside (17) [66], (7*S*,8*R*)-3,3′-dimethoxy-4-9-9′-trihydroxy-4′,7-epoxy-5,8′-lignan-4,9-bis-*O*-*β*-_D_-glucopyranoside (18) [67], *threo*-(7*S*,8*S*)-guaiacyl-glycerol-*β*-*O*-4′-dihydroconiferyl ether (19) [68], *erythro*-(7*R*,8*S*)-guaiacyl-glycerol-*β*-*O*-4′-dihydroconiferyl ether (20) [69], *erythro*-(7*R*,8*S*)-4,7,9,9′-tetrahydroxy-3,5,2′-trimethoxy-8-*O*-4′-neolignan (21) [69], *erythro*-(7*R*,8*S*)-4,7,9,9′-tetrahydroxy-3,3′-dimethoxy-8-*O*-4′-neolignan-9′-*O*-*β*-_D_-glucopyranoside (22) [70], *threo*-(7*R*,8*R*)-4,7,9,9′-tetrahydroxy-3,3′-dimethoxy-8-*O*-4′-neolignan-9′-*O*-*β*-_D_-glucopyranoside (23) [70], *erythro*-(7*R*,8*S*)-7,9,9-trihydroxy-3,3,5-trimethoxy-8-*O*-4-neolignan-4-*O*-*β*-_D_-glucopyranoside (24) [71], 2-[4-(3-hydroxypropyl)-2-methoxyphenoxy]propane-1,3-diol (25) [72], 2-[4-(3-hydroxy-propyl)-2,6-dimethoxyphenoxy]-propane-1,3-diol (26) [73], salicifoliol (27) [74], 4-ketopinoresinol (28) [75], (+)-pinoresinol (29) [76], (+)-medioresinol (30) [77], (+)-syringaresinol (31) [78], (7*R*,7′*R*,7^″^*R*,8*S*,8′*S*,8^″^*S*)-4′,4^″^-dihydroxy-3,3′,3^″^-triethoxy-7,9′:7′,9-diepoxy-4,8^″^-oxy-8,8′-sesquineolignan-7^″^,9^″^-diol (32) [78], (7*S*,7′*S*,7^″^*S*,8*R*,8′*R*,8^″^*R*)-4′,4^″^-dihydroxy-3,3′,3^″^,5-tetramethoxy-7,9′:7′,9-diepoxy-4,8^″^-oxy-8,8′-sesquineolignan-7^″^,9^″^-diol (33) [78], (7*R*,7′*R*,7^″^*R*,8*S*,8′*S*,8^″^*S*)-4′,4^″^-dihydroxy-3,3′,3^″^,5-tetramethoxy-7,9′:7′,9-diepoxy-4,8^″^-oxy-8,8′-sesquineolignan-7^″^,9^″^-diol (34) [78], (7*R*,7′*R*,7^″^*R*,8*S*,8′*S*,8^″^*S*)-4′,4^″^-dihydroxy-3,3′,3^″^,5,5′-pentamethoxy-7,9′:7′,9-diepoxy-4,8^″^-oxy-8,8′-sesquineolignan-7^″^,9^″^-diol (35) [79], (7*S*,7′*S*,7^″^*S*,8*R*,8′*R*,8^″^*R*)-4′,4^″^-dihydroxy-3,3′,3^″^,5,5′,5^″^-hexamethoxy-7,9′:7′,9-diepoxy-4,8^″^-oxy-8,8′-sesquineolignan-7^″^,9^″^-diol (36) [80], vanillic acid (37) [80], protocatechuic acid (38) [81], 4-hydroxybenzoic acid (39) [82], *p*-hydroxybenzaldehyde (40) [83], isovanillic acid (41) [84], protocatechuic acid methyl ester (42) [85], 3,4-dimethoxybenzyl alcohol (43) [86], variecolorquinones A (44) [87], physcion (45) [88], 1*H*-indole-3-carbaldehyde (48) [89], uridine (49) [90], and aurantiamide acetate (50) [91] by comparing their spectroscopic data w-ith data reported in the literatures (*Supplementary Materials*).

### 3.2. Potential Antidiabetic Effect Predicted by Network Pharmacology Analysis

**Figure 3:**
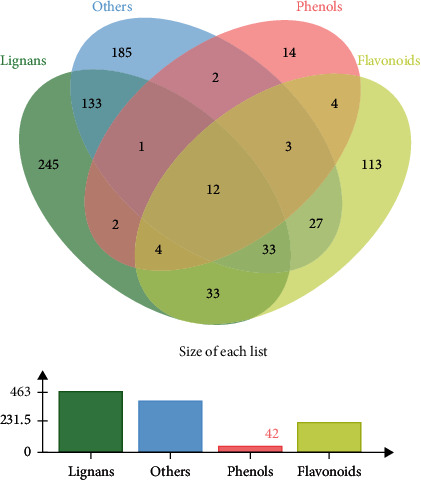
The relationship between the number of candidate targets and the types of compounds of FLC. The green represents the lignans in FLC, the yellow represents the flavonoids in FLC, the red represents the phenols in FLC, and the blue represents the other compounds in FLC.

**Figure 4:**
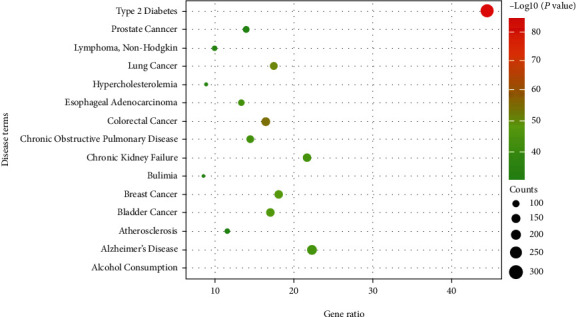
The disease annotation analysis of the candidate targets for FLC. The size represents the counts of the targets, the color represents −log10 (*P* value).

In our previous study, we summarized 65 compounds that had been isolated from FLC through literature research [8]. In order to build the chemical ingredient database of FLC, we collected 105 compounds in all, consisting of the reported ingredients and those which were isolated in our laboratory. Additionally, the database mainly included flavonoids, ligands, phenols, fatty acids, and alkaloids. And then, a total of 811 candidate targets of ingredients were searched from PubChem, SwissTargetPrediction, and SuperPred databases. The relationship between the number of targets and the types of compounds was displayed in Figure 3. To investigate the diseases related to the candidate targets, the targets were mapped to DAVID to systematically analyze potential related diseases. The result shown in Figure 4 illustrated that FLC mainly had a potential effect against type 2 diabetes, cancer (such as prostate cancer, lung cancer, esophageal adenocarcinoma, breast cancer, and bladder cancer), and so on. Type 2 diabetes ranked top one with the smallest *P* value (1.19 × 10^−84^), the largest count (324) and the highest gene ratio (44.57%). It had been reported that the extracts of FLC showed antiproliferative activities *in vitro* (such as lung cancer A549, human liver cancer HepG2, and breast cancer MCF-7) and *in vivo* [8, 10, 12]. However, the anti-diabetic function of FLC has not been reported to date. Typical habits of modern societies push individuals toward a nutritional overload lifestyle. This disturbing reality is evidenced by the exponential rise in the prevalence of type 2 diabetes. And research showed that diabetes patients had an increased risk of developing tumors, and diabetic patients with tumors had a poor prognosis and a high risk of death, because chemotherapy drugs could reduce insulin secretion, aggravate glucose metabolism disorders, and increase the incidence of acute complications such as hypoglycemia, ketoacidosis, and even death [92]. Thus, FLC possessed the potential to be developed as functional food for diabetic patients with tumors.

The therapeutic targets for type 2 diabetes were collected from Comparative Toxicogenomic Database, Therapeutic Target Database and DrugBank database. Six hundred and eighty-one overlapped targets were both related to FLC and type 2 diabetes. The overlapped targets were mapped to DAVID to systematically analyze their biological processes [27]. According to the result of GO enrichment analysis (Figure S121), the biological process of FLC against diabetes might involve in the regulation of inflammatory response, oxidation-reduction process, protein phosphorylation, insulin secretion, glucose metabolic process, and so on. To understand the potential mechanism of FLC against diabetes, KEGG pathway enrichment analysis was applied. The results (Figure S122) of the analysis demonstrated that the pathways of FLC against diabetes including PI3K-Akt, MAPK, RAS, insulin, insulin resistance, TNF, FoxO, and cancer-related signaling pathways. According to the analysis of the ingredient-target network, 15 key targets of FLC against diabetes identified with network analyzer were listed in Table S1. Among the key targets, PTGS1, PTGS2, and ALOX5 were closely related to inflammation. GAA was the gene encoded *α*-glucosidase in lysosome, and PTPN1 was the gene encoded PTP1B protein.

### 3.3. Antioxidant Capacity

The scavenging capacities of the extract, fractions, and all isolates (1–50) were investigated against two free radical DPPH and ABTS·+ in concentration-dependent manners as shown in Tables 2 and 3. Ascorbic acid was used as a reference compound to validate the inhibition results. On the results of the DPPH and ABTS assays, the most active fraction was the butyl alcohol fraction (IC_50_ = 4.23 *μ*g/mL (for DPPH), IC_50_ = 8.70 *μ*g/mL (for ABTS)). The alkaloids isolated from FLC were inactive in free radical scavenging; especially, the purine derivatives were cytokinins [45]. It was noteworthy that the antioxidant activities of compounds 1, 3, 4, and 12 (IC_50_ values ranged from 26.38 to 46.80 *μ*M for DPPH and from 11.29 to 43.10 *μ*M for ABTS) were comparable to that of the positive control ascorbic acid (IC_50_ = 35.11 *μ*M for DPPH, IC_50_ = 50.90 *μ*M for ABTS). The dihydroxy (3′,4′-diOH, catechol) in the B ring was beneficial for free radical scavenging, which conferred high stability to the flavonoid phenoxyl radicals via hydrogen bonding or by expanded electron delocalization [93]. Compound 13 (IC_50_ = 24.62 *μ*M for ABTS), which was different from compounds 15 and 16 (>100 *μ*M) by the position of the 4-OH group, displayed superior scavenging capacity against ABTS·+. This implied that the phenolic hydroxyl group (4-OH) of benzofuran neolignans in FLC was substituted, and the antioxidant capacity was radically decreased. Interestingly, 8-O-4′ neolignans (compound 19-23), sesquineolignans (compounds 32-36), and furofuran lignans (compounds 28-31) were all with at least one phenolic hydroxyl group, and they possessed significant scavenging capacities, especially against ABTS·+. Therefore, the flavonoids with a catechol unit and the lignans with a phenolic hydroxyl group isolated from FLC could be an indelible factor for the antioxidant capacity.

### 3.4. Inhibitory Effects on the NO Release

To evaluate their anti-inflammatory activities, the effects of all the compounds isolated from FLC on the LPS-stimulated NO release were determined in RAW 264.7 cells. The IC_50_ values of active compounds and fractions with no significant cytotoxicity were displayed in Tables 2 and 3. The most active fraction was the dichloromethane fraction (IC_50_ = 59.19 *μ*g/mL), followed by the ethyl acetate fraction (IC_50_ = 62.87 *μ*g/mL). The lignans in FLC showed superior anti-inflammatory activity (IC_50_ < 100 *μ*M, except compounds 18 and 24) compared with the flavanols (inactive) and the phenylpropanoids (inactive). Substitution at the 4-OH position of benzofuran neolignans (compounds 15 and 16) in FLC caused an increase of the anti-inflammatory activity, compared with compound 13. Glucose substitution at the 9-OH position (compound 18) was inactive, compared with compound 17. It can be concluded that the hydroxyl positions of benzofuran neolignans in FLC could affect the anti-inflammatory activity. Compound 28 (IC_50_ = 14.78 *μ*M) exhibited higher inhibitory effect than compound 27 (IC_50_ = 66.86 *μ*M), suggested that furofuranone lignans with double aromatic rings were more active than that with a single aromatic ring. Among the lignans, compounds 15, 17, and 28 showed the strongest anti-inflammatory effect with IC_50_ from 13.18 *μ*M to 17.29 *μ*M, while IC_50_ of the positive control indomethacin was 22.39 *μ*M. Above all, the lignans in FLC play an important role in inhibiting LPS-stimulated NO release.

### 3.5. *α*-Glucosidase Inhibitory Activity

Inhibition of *α*-glucosidase or PTP1B enzymes was an effective strategy to treat metabolic diseases such as diabetes. All isolated compounds and fractions were assayed *in vitro* for their *α*-glucosidase and PTP1B inhibitory activities. The results were presented in Tables 2 and 3. The results showed that the butyl alcohol fraction had the highest inhibitory effect of *α*-glucosidase with an IC_50_ value of 8.27 *μ*g/mL. The substitution of glucose at the 9′-OH position of 8-*O*-4′ neolignans (compounds 22 and 23) caused a decrease of *α*-glucosidase inhibition compared with those with no substitution (compounds 19 and 20). In addition, the IC_50_ values of compounds 1, 4, 29-36, and 44 ranged from 5.71 to 52.07 *μ*M, and the IC_50_ value of acarbose was 61.34 *μ*M. This revealed that flavanols, furofuran lignans, and sesquineolignans in FLC mainly possessed significant inhibitory activity of *α*-glucosidase.

### 3.6. PTP1B Inhibitory Activity

The results showed that the butyl alcohol fraction had the highest inhibitory effect of PTP1B with an IC_50_ value of 0.017 *μ*g/mL. The sesquineolignans in FLC performed stronger inhibitory activity of *α*-glucosidase than that of PTP1B. Besides, the catechol derivatives (compounds 1, 4, 42) displayed appropriate inhibition activities against PTP1B and antioxidant. It had been reported that some catechol derivatives were successfully synthesized as PTP1B inhibitors, implying that the catechol moiety played a key role in the PTP1B inhibitory activity [94]. The compounds with catechol moiety were main flavonoids in FLC. Evidence from studies in experimental animals suggested that dietary (-)-epicatechin (compound 4) could mitigate insulin resistance through the modulation of redox-regulated mechanisms in a rat model of metabolic syndrome [95]. Compounds 1, 6, and 44 exhibited the most remarkable activity with IC_50_ values ranging from 9.41 to 22.19 *μ*M, while that of the positive control oleanolic acid was 28.58 *μ*M. They were potential PTP1B inhibitors; therefore, they were used to explore the inhibitory mechanism.

#### 3.6.1. Inhibitory Kinetics and Binding Affinities to PTP1B

**Figure 5:**
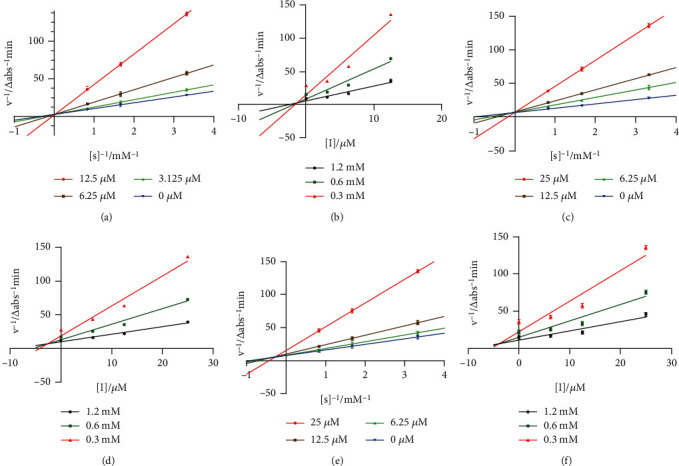
Lineweaver-Burk plots for PTP1B inhibition of compounds 1 (a), 44 (c), and 6 (e). Dixon plots for PTP1B inhibition of compounds 1 (b), 44 (d), and 6 (f). Each value was presented as mean ± SD, *n* = 3.

To study the kinetic behavior of the most active compounds (compounds 1, 6, and 44) against PTP1B, Lineweaver−Burk double reciprocal plot and Dixon plot were further applied. As displayed in Figures 5(a) and 5(c), compounds 1 and 44 were identified as competitive inhibitors, the regression lines for each compound intersected at the *y*-axis. And compound 6 was educed as a mixed-type inhibitor for the reason that increasing concentration of inhibitor results in the family of lines shared a common intercept on the left of the *y*-axis and above the *x*-axis. Based on the results of Dixon plots (Figures 5(b), 5(d), and 5(f)), the inhibition constant (*K*_*i*_) values (Table 4) of compounds 1, 44, and 6 were 1.49, 2.80, and 4.18 *μ*M, respectively.

**Figure 6:**
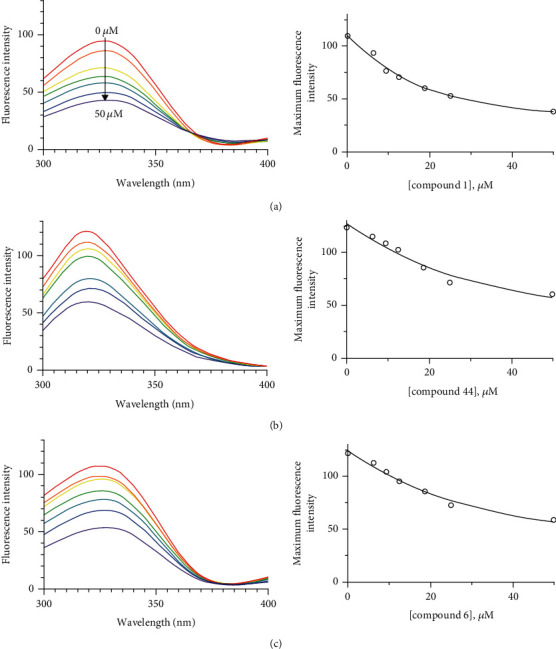
The fluorescence emission spectra of PTP1B at different concentrations (0, 6.25, 9.37, 12.5, 18.75, 25, and 50 *μ*M) of compounds 1 (a), 44 (b), and 6 (c). Normalized intensities of fluorescence for PTP1B are shown.

The PTP1B enzyme has a large number of intrinsic fluorescence residues consisting of 19 phenylalanines, 13 tyrosines, and 18 tryptophans. Therefore, the intrinsic fluorescence might be changed by the function of inhibitor affinity [96]. Then, we investigated the interactions between PTP1B and the potential inhibitors (compounds 1, 6, and 44) that had an obvious difference in inhibitory potencies at different concentrations. There was no significant emission from any assay mixture (including the inhibitors) under our measurement conditions.  Figure 6 showed the fluorescence emission spectra of PTP1B at different concentrations (0, 6.25, 9.37, 12.5, 18.75, 25, and 50 *μ*M) of the potential inhibitors. The dose-dependent lowering of the fluorescence intensity was observed on the increase of the concentrations of inhibitors. Importantly, the decreasing tendency of fluorescence quenching was highly correlated with inhibitory potencies (IC_50_). The binding affinity constant (*K*_SV_) was calculated using Stern-Volmer equation (Table 4). The *K*_SV_ values were ranked in order of inhibitory potencies as follows: compound 1 (IC_50_ = 9.41 *μ*M, *K*_SV_ = 0.37 × 10^5^ L/mol)>compound 44 (IC_50_ = 15.85 *μ*M, *K*_SV_ = 0.23 × 10^5^ L/mol)>compound 6 (IC_50_ = 22.19 *μ*M, *K*_SV_ = 0.22 × 10^5^ L/mol). The binding affinities (*K*_SV_) had a high correlation (*R*^2^ = 0.94) with inhibitory potencies (IC_50_) (Figure S123).

#### 3.6.2. Molecular Docking Analysis

**Figure 7:**
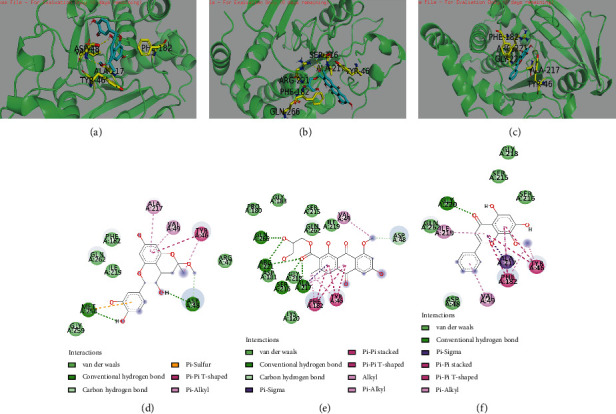
3D molecular docking model for the ligand interactions of compounds 1 (a), 44 (b), and 6 (c); yellow dashed lines indicate H-bonds; the residues of PTP1B are yellow sticks; the compounds are blue sticks. 2D ligand interaction diagrams of compounds 1 (d), 44 (e), and 6 (f) in the PTP1B enzyme.

To further study the binding modes of these potent PTP1B inhibitors, the interactions of PTP1B with the inhibitors (compounds 1, 6, and 44) were studied *in silico*. The compounds with the smallest binding energy of PTP1B (PDB ID: 1AAX) and the optimized binding pose were displayed in Figure 7 through molecular docking study, respectively. The 3D and 2D binding mode as shown in Figures 7(a) and 7(d) of compound 1 in the best site of PTP1B with the smallest binding energy (-4.7 kcal/mol). Compound 1 formed two hydrogen bonds, including the 3-OH with residues Asp48 and the phenolic hydroxyl group on the B ring with residues Met258. And the *π*-*π* and *π*-alkyl hydrophobic interactions were connected with residues Tyr46, Ala217, and Val49. The analysis also indicated that compound 1 was surrounded by Arg47, Phe182, Ile219, Gly259, and Gln262. These suggested that compound 1 was located in the secondary phosphate-binding active site, and it could affect the recognition and binding affinity of the substrate [97]. Figures 7(b) and 7(e) showed the pose of compound 44 in PTP1B with the binding energy (-6.1 kcal/mol), surrounded by Lys120, Ile219, and Gln62. Compound 44 formed hydrogen bonds with residues Ser216, Ala217, Arg221, and Gln266. It also formed *π*-*σ* interaction with residues Ala217, *π*-*π* interactions with Tyr46 and Phe182, and *π*-alkyl interaction with residues Val49. The result directed that compound 44 bonded to the catalytic site (Arg221) and then blocked the dephosphorylation of the substrate [98]. The pose (Figure 7(c) and 7(f)) of compound 6 molecule in the PTP1B crystal structure with the smallest binding energy (-6.2 kcal/mol), which was surrounded by Asp48, Ser216, and Gly218. It formed *π*-*σ* interaction with residues Ala217, *π*-*π* interactions with Tyr46 and Phe182, and *π*-alkyl interaction with residues Val49 and Ile219. Based on the molecular docking analysis, the potential PTP1B inhibitors were all located in the active sites. However, according to kinetic mode, compounds 1 and 44 were competitive inhibitors; only compound 44 interacted with the catalytic residues. It was deduced that the issue might be caused by the limitation of molecular docking; thus, molecular dynamics simulations were further applied.

#### 3.6.3. Molecular Dynamics Simulations

**Figure 8:**
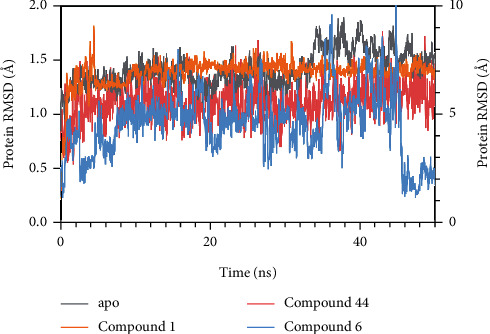
The RMSD plots of PTP1B apo protein and PTP1B-the compound complex monitored during the whole molecular dynamics simulations. RMSD plots of PTP1B apo protein (grey, plot data on left *Y* axis), PTP1B-compound 1 complex (orange, plot data on right *Y* axis), PTP1B-compound 44 complex (red, plot data on left *Y* axis), and PTP1B-compound 6 complex (blue, plot data on right *Y* axis).

In fact, proteins are dynamic macromolecules under physiological conditions, and the ligand-receptor interactions derived from molecular docking may be less persuasive compared with molecular dynamics simulations. To predict the stability and flexibility of the binding modes, the protein of PTP1B- and PTP1B-compound complexes were submitted to molecular dynamics simulations, respectively. Additionally, RMSD could reflect the changes of protein structural conformation and suggest whether the molecular dynamics simulations had reached the equilibrium. As shown in Figure 8, during the whole molecular dynamics simulations, the RMSD values of protein-compound complexes were monitored. The PTP1B-compound 1 complex reached equilibrium after 11.4 ns, where the protein RMSD value fluctuated between 6.71 Å and 7.82 Å. The PTP1B-compound 44 complex reached equilibrium after 3.9 ns, and the protein RMSD value fluctuated between 0.60 Å and 1.75 Å. The PTP1B-compound 6 complex reached equilibrium after 45 ns, where the protein RMSD value fluctuated between 1.5 Å and 3.0 Å (Figure 8(c)). The above results indicated that the PTP1B-compound 1 complex and PTP1B-compound 44 complex were more stable than PTP1B-compound 6 complex after the systems reached equilibrium, which indicated that compound 1 and compound 44 had a better binding affinity. And the RMSD value also suggested that compound 1 might deviate molecular docking position.

**Figure 9:**
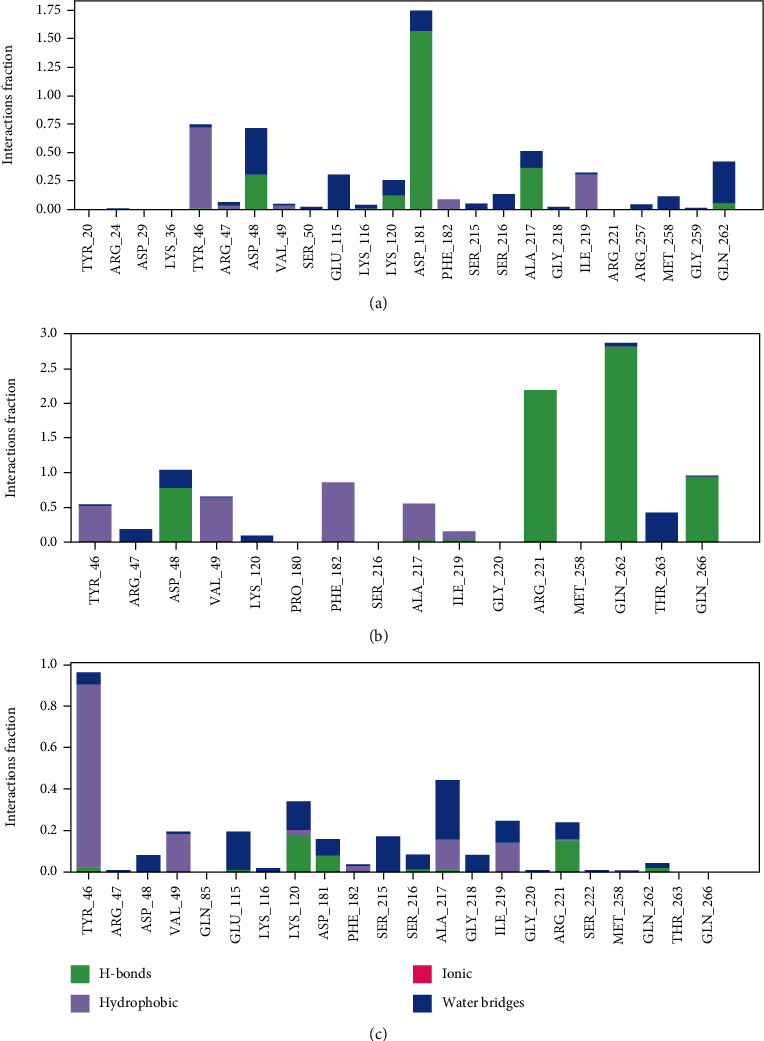
The histogram of PTP1B-the compound interactions monitored during the whole molecular dynamics simulations. (a) Interaction fraction of PTP1B-compound 1 complex. (b) Interaction fraction of PTP1B-compound 44 complex. (c) Interaction fraction of PTP1B-compound 6 complex.

The histograms (Figure 9) showed the type and proportion of interactions between receptor and ligand. In Figure 9(a), compound 1 formed strong hydrogen bonds with catalytic active site (Asp181), which accounted for 152% of the simulation time. It also formed hydrogen bonds and water bridges with residues Asp48, Lys120, and catalytic active residue Gln262. Meanwhile, residues of Tyr46, Ala217, and Ile219 formed hydrophobic contacts with compound 1, accounting for 73%, 38%, and 35% of the simulation time, respectively. In conclusion, hydrogen bonds and hydrophobic contacts were the main interactions between PTP1B and compound 1. In Figure 9(b), compound 44 formed hydrogen bonds with catalytic active sites Arg221 and Gln262, which accounted for 280% and 220% of the simulation time, respectively. In addition, compound 44 formed hydrophobic contacts with residues of Phe182, Val49, Ala217, and Tyr46, accounting for 90%, 60%, 54%, and 51% of the simulation time, respectively. As shown in Figure 9(c), compound 6 formed hydrophobic contacts with residues Tyr46 and Val49, accounting for 90% and 19% of the simulation time, respectively. At the same time, it also bounded to residues Lys120 and Arg221 with hydrogen bonds.

The results obtained from the molecular dynamics simulations indicated that compounds 1 and 44 formed better bind affinity, especially with catalytic active sites (Asp181, Arg221, and Gln262), compared with compound 6. It also explained that compounds 1 and 44 were potential competitive inhibitors of PTP1B with better biological activity, which was consistent with kinetic mode and made up the shortage of molecular docking analysis [94]. The results above all further deepen the understanding of the inhibitory mechanism of potential competitive PTP1B inhibitors.

### 3.7. Glucose Uptake Stimulations

**Figure 10:**
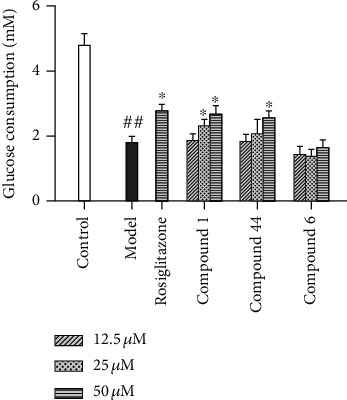
Effects on glucose consumption of the insulin-resistant HepG2 cells. Each value was presented as mean ± SD (*n* = 5). ^∗^*P* < 0.05 vs. the model group, ^##^*P* < 0.01 vs. the control group.

Encouraged by the result of the PTP1B inhibition assay, compounds 1, 44, and 6 were tested for their effects on glucose uptake in insulin-resistant HepG2 cells. All tested compounds showed no cytotoxicity against HepG2 cells in the concentration range from 12.5 to 50 *μ*M. At the dose of 50 *μ*M, compounds 1 and 44 increased glucose consumption by 48.9% and 42.8%, respectively, which were similar to that of the positive control of rosiglitazone (46.7%) in an insulin resistance model of HepG2 cells as shown in Figure 10. Thus, compounds 1 and 44 were also potential insulin sensitizers. However, compound 6 displayed little effect on glucose uptake.

## 4. Conclusions

In conclusion, we studied the novel potential antidiabetic function predicted by chemical profile and network pharmacology. This provided a new strategy to identify the potential pharmacological activity. The bioactive compounds responsible for the antioxidant, anti-inflammatory, and antidiabetic activities were identified. In addition, the competitive inhibitors of PTP1B (compounds 1 and 44) formed strong binding affinity with catalytic active sites and they were also potential insulin sensitizers. With the attained results, it could be speculated that FLC could be developed as nutraceuticals for the prevention and treatment of diabetes.

## Figures and Tables

**Table 1:** ^1^H (600 MHz) and ^13^C NMR (150 MHz) data of compounds 1, 46, and 47 in DMSO-*d*_6_.

Position	Compound 1		Compound 46		Compound 47	
	*δ*_C_	*δ*_H_ mult. (*J* in Hz)	*δ*_C_	*δ*_H_ mult. (*J* in Hz)	*δ*_C_	*δ*_H_ mult. (*J* in Hz)
2	78.2	5.12, d (5.1)	150.9	8.02, s	151.3	8.02, s
3	69.2	3.7, dd (11.1, 5.1)				
4	25.6	2.54, m	147.4		147.5	
5	152.6		104.7		104.9	
6	95.0	5.79, d (2.3)	145.9		146.0	
7	157.6					
8	94.5	5.87, d (2.3)	152.9	10.19, brs	153.3	10.06, brs
9	153.4			11.22, brs		11.20, brs
10	98.2			6.55, t (5.8)		6.51, t (5.3)
11	34.1	1.29, t (12.8) 2.13, dd (12.8, 4.9)	44.6	3.67, ddd (13.4, 6.4, 4.2) 3.31, m	38.0	4.03, t (6.2)
12	98.8		72.9	4.07, dd (7.6, 4.2)	120.0	5.50, tp (6.2, 1.5)
13	23.4	1.45, s	146.3		139.0	
14	48.7	3.17, s	111.2	4.97, dt (2.3, 1.1) 4.82, t (2.3)	66.1	3.81, s
15			18.5	1.72, s	14.1	1.63, s
1′	130.6					
2′	114.7	6.69, d (2.1)				
3′	144.4					
4′	144.4					
5′	114.7	6.62, d (8.2)				
6′	118.1	6.51, dd (8.2, 2.1)				

**Table 2:** Antioxidant, anti-inflammatory, *α*-glucosidase, and PTP1B inhibitory activities of ethanol extract and fractions from the fruits of *Livistona chinensis*.

Extract and fractions	IC_50_ (*μ*g/mL)^a^				
	DPPH scavenging activity	ABTS scavenging activity	NO production anti-inflammatory effect	*α*-Glucosidase inhibitory effect	PTP1B inhibitory effect
Ethanol extract	47.00 ± 0.47	36.45 ± 1.31	111.63 ± 1.40	175.30 ± 2.59	10.18 ± 1.10
Petroleum ether fraction	>300	>300	—	—	—
Dichloromethane fractions	146.47 ± 1.42	18.96 ± 0.28	59.19 ± 0.93	133.70 ± 4.82	42.98 ± 0.42
Ethyl acetate fraction	51.52 ± 0.92	16.29 ± 0.99	62.87 ± 0.75	167.10 ± 3.70	24.10 ± 1.30
Butyl alcohol fraction	4.23 ± 0.11	8.70 ± 0.50	—	8.27 ± 0.88	0.017 ± 0.003
Aqueous fraction	160.03 ± 1.70	73.80 ± 2.12	>300	>300	198.90 ± 1.64
Ascorbic acid^b^	6.18 ± 0.18	8.96 ± 0.22	—	—	—
Indomethacin^c^	—	—	8.01 ± 0.08	—	—
Acarbose^d^	—	—	—	39.60 ± 0.66	—
Oleanolic acid^e^	—	—	—	—	13.06 ± 0.28

^a^These data are expressed as the mean value ± SD of triplicate experiments (*n* = 3). ^b^Positive control (DPPH and ABTS scavenging activities). ^c^Positive control (anti-inflammatory effect). ^d^Positive control (*α*-glucosidase inhibitory effect). ^e^Positive control (PTP1B inhibitory effect).

**Table 3:** Antioxidant, anti-inflammatory, *α*-glucosidase, and PTP1B inhibitory activities of compounds 1-50 from the fruits of *Livistona chinensis*.

Compound	IC_50_ (*μ*M)^a^				
	DPPH scavenging activity	ABTS scavenging activity	NO production anti-inflammatory effect	*α*-Glucosidase inhibitory effect	PTP1B inhibitory effect
1	46.80 ± 1.53	34.91 ± 0.07	—	37.27 ± 0.74	9.41 ± 0.08
2	43.42 ± 0.46	15.00 ± 0.11	—	62.49 ± 1.92	39.25 ± 0.14
3	26.38 ± 0.39	11.29 ± 0.09	—	89.00 ± 1.95	91.78 ± 0.68
4	32.01 ± 0.37	13.46 ± 0.09	—	24.03 ± 0.38	38.66 ± 0.21
5	63.99 ± 0.67	53.56 ± 0.26	—	>100	>100
6	99.36 ± 0.44	>100	49.40 ± 1.85	>100	22.19 ± 0.58
7	78.76 ± 0.61	63.11 ± 0.24	70.79 ± 0.67	>100	30.21 ± 0.45
8	61.25 ± 0.31	24.09 ± 0.32	—	>100	>100
9	92.26 ± 0.32	85.04 ± 0.24	—	98.60 ± 1.20	30.64 ± 0.48
10	>100	91.08 ± 1.10	65.83 ± 1.47	>100	—
11	21.78 ± 0.26	36.71 ± 0.33	—	>100	90.21 ± 0.37
12	46.46 ± 0.40	43.10 ± 0.29	—	>100	>100
13	87.78 ± 0.74	24.62 ± 0.31	76.69 ± 0.51	88.62 ± 0.49	>100
14	60.3 ± 0.16	45.13 ± 0.41	69.98 ± 5.33	62.17 ± 1.03	>100
15	>100	>100	13.18 ± 0.36	>100	>100
16	>100	>100	36.71 ± 0.80	>100	>100
17	>100	33.49 ± 0.31	17.29 ± 0.60	>100	>100
18	>100	23.25 ± 0.11	—	>100	>100
19	>100	54.67 ± 0.34	43.65 ± 0.9	58.37 ± 0.44	95.63 ± 0.47
20	>100	60.92 ± 0.46	53.16 ± 0.24	52.59 ± 1.33	93.56 ± 1.07
21	>100	93.00 ± 0.54	55.54 ± 0.41	61.75 ± 0.76	66.99 ± 1.20
22	>100	26.97 ± 0.21	83.16 ± 0.24	73.07 ± 2.33	92.39 ± 0.71
23	>100	68.17 ± 0.76	73.16 ± 0.34	82.12 ± 0.56	90.06 ± 0.47
24	>100	>100	—	—	>100
26	>100	>100	—	56.52 ± 0.45	—
27	>100	41.98 ± 0.33	66.86 ± 0.51	>100	—
28	>100	20.37 ± 0.23	14.78 ± 1.20	>100	—
29	77.36 ± 2.98	18.49 ± 0.09	69.94 ± 0.31	52.07 ± 0.93	90.19 ± 1.87
30	95.67 ± 2.44	25.58 ± 0.12	55.54 ± 1.85	34.38 ± 0.50	26.75 ± 0.21
31	65.44 ± 1.48	23.73 ± 0.25	48.98 ± 0.39	49.05 ± 1.10	—
32	96.38 ± 2.26	40.63 ± 0.23	46.18 ± 0.67	5.71 ± 0.17	81.93 ± 0.78
33	66.67 ± 0.52	33.43 ± 0.45	47.35 ± 0.85	50.88 ± 1.78	62.13 ± 0.73
34	68.71 ± 2.19	37.98 ± 0.43	31.20 ± 0.22	32.83 ± 1.45	80.93 ± 0.55
35	65.94 ± 1.52	31.18 ± 0.24	26.20 ± 0.19	19.65 ± 0.21	63.16 ± 0.30
36	62.44 ± 0.42	31.10 ± 0.44	79.69 ± 1.17	22.73 ± 1.07	63.19 ± 0.33
37	>100	88.32 ± 0.12	—	—	—
38	33.07 ± 0.79	63.25 ± 0.51	—	—	44.75 ± 0.12
41	>100	76.06 ± 0.48	—	—	91.10 ± 0.38
42	63.32 ± 0.41	83.21 ± 0.53	—	—	62.96 ± 0.65
44	23.05 ± 0.27	12.96 ± 0.27	—	29.61 ± 0.40	15.85 ± 0.29
45	>100	>100	29.98 ± 0.57	—	24.75 ± 0.18
Ascorbic acid^b^	35.11 ± 1.02	50.90 ± 1.25	—	—	—
Indomethacin^c^	—	—	22.39 ± 0.22	—	—
Acarbose^d^	—	—	—	61.34 ± 1.02	—
Oleanolic acid^e^	—	—	—	—	28.58 ± 0.62

^a^These data are expressed as the mean value ± SD of triplicate experiments (*n* = 3). ^b^Positive control (DPPH and ABTS scavenging activities). ^c^Positive control (anti-inflammatory effect). ^d^Positive control (*α*-glucosidase inhibitory effect). ^e^Positive control (PTP1B inhibitory effect).

**Table 4:** Fluorescence quenching effect and inhibitory effect of compounds 1, 44, and 6 on PTP1B.

Compounds	*K*_SV_ (×10^5^L/mol)	*R* ^a^	*K*_A_ (×10^5^L/mol)	*R* ^b^	*n*	Inhibitory mode^c^	*K*_*i*_ (*μ*M)^d^
1	0.37	0.99	0.95	0.98	1.08	Competitive	1.49
44	0.23	0.97	0.58	0.95	0.97	Competitive	2.80
6	0.22	0.98	0.13	0.97	1.12	Mixed-type	4.18

^a^The correlation coefficient for the *K*_SV_ values. ^b^The correlation coefficient for the *K*_A_ values. ^c^Determined by Lineweaver-Burk plots. ^d^Determined by Dixon plots.

## Data Availability

The data used to support the findings of this study are available from the corresponding author upon request.

## Supplementary Materials

Supplementary Materials Figures S1–S28: the HR-ESI-MS, ^1^H, ^13^C-NMR, HSQC, HMBC, ^1^H-^1^H COSY, NOESY, UV, CD, and IR spectrums of compounds 1, 46, and 47. Figures S29–S120: the ^1^H, ^13^C-NMR spectrums and HR-ESI-MS of known compounds. Figures S121 and S122: GO and KEGG enrichment analyses of the candidate targets, respectively. Figure S123: the correlation between PTP1B IC_50_ values and Stern-Volmer constants (*K*_sv_) of compounds 1, 6, 30, 44, and 45. Table S1: fifteen key targets of the fruits of *Livistona chinensis* against diabetes identified with a network analyzer. The identification methodologies of known compounds were indicated in Supplementary Materials.
