# Supplementary material for: Antioxidant, Anti-Inflammatory, and Antidiabetic Activities of Bioactive Compounds from the Fruits of Livistona chinensis Based on Network Pharmacology Prediction
Source: Oxid Med Cell Longev. 2021 Oct 18;2021:7807046. doi: 10.1155/2021/7807046 (PMC8545574; doi:10.1155/2021/7807046)
Supplement: Supplementary Materials — Figures S1–S28: the HR-ESI-MS, 1H, 13C-NMR, HSQC, HMBC, 1H-1H COSY, NOESY, UV, CD, and IR spectrums of compounds 1, 46, and 47. Figures S29–S120: the 1H, 13C-NMR spectrums and HR-ESI-MS of known compounds. Figures S121 and S122: GO and KEGG enrichment analyses of the candidate targets, respectively. Figure S123: the correlation between PTP1B IC50 values and Stern-Volmer constants (Ksv) of compounds 1, 6, 30, 44, and 45. Table S1: fifteen key targets of the fruits of Livistona chinensis against diabetes identified with a network analyzer. The identification methodologies of known compounds were indicated in Supplementary Materials. [file 7807046.f1.docx]

**Supplementary Materials**

**Antioxidant, Anti-inflammatory and Antidiabetic Activities of Bioactive Compounds from the Fruits of *Livistona chinensis* Based on Network Pharmacology Prediction**

**Yuwei Wang, Jianxiu Zhai, Dan Yang, Na Han, Zhe Liu, Zhihui Liu, Sikai Li, and Jun Yin***

*Department of Pharmacognosy and Utilization Key Laboratory of Northeast Plant Materials, School of Traditional Chinese Medicine, Shenyang Pharmaceutical University, Shenyang, 110016, China*

Correspondence should be addressed to Jun Yin; yinjun826@sina.com

Index

[Figure S1. HR-ESI-MS spectrum of compound **1** 5](#_Toc82601231)

[Figure S2. ^1^H NMR spectrum of compound **1** in DMSO-*d*_6_ 5](#_Toc82601232)

[Figure S3. ^13^C NMR spectrum of compound **1** in DMSO-*d*_6_ 6](#_Toc82601233)

[Figure S4. HSQC spectrum of compound **1** in DMSO-*d*_6_ 6](#_Toc82601234)

[Figure S5. HMBC spectrum of compound **1** in DMSO-*d*_6_ 7](#_Toc82601235)

[Figure S6. ^1^H – ^1^H COSY spectrum of compound **1** in DMSO-*d*_6_ 7](#_Toc82601236)

[Figure S7. NOESY spectrum of compound **1** in DMSO-*d*_6_ 8](#_Toc82601237)

[Figure S8. UV spectrum of compound **1** 8](#_Toc82601238)

[Figure S9. CD spectrum of compound **1** 9](#_Toc82601239)

[Figure S10. IR spectrum of compound **1** 9](#_Toc82601240)

[Figure S11. HR-ESI-MS spectrum of compound **46** 10](#_Toc82601241)

[Figure S12. ^1^H NMR spectrum of compound **46** in DMSO-*d*_6_ 10](#_Toc82601242)

[Figure S13. ^13^C NMR spectrum of compound **46** in DMSO-*d*_6_ 11](#_Toc82601243)

[Figure S14. HSQC spectrum of compound **46** in DMSO-*d*_6_ 11](#_Toc82601244)

[Figure S15. HMBC spectrum of compound **46** in DMSO-*d*_6_ 12](#_Toc82601245)

[Figure S16. ^1^H – ^1^H COSY spectrum of compound **46** in DMSO-*d*_6_ 12](#_Toc82601246)

[Figure S17. NOESY spectrum of compound **46** in DMSO-*d*_6_ 13](#_Toc82601247)

[Figure S18. IR spectrum of compound **46** 13](#_Toc82601248)

[Figure S19. UV spectrum of compound **46** 14](#_Toc82601249)

[Figure S20. HR-ESI-MS spectrum of compound **47** 14](#_Toc82601250)

[Figure S21. ^1^H NMR spectrum of compound **47** in DMSO-*d*_6_ 15](#_Toc82601251)

[Figure S22. ^13^C NMR spectrum of compound **47** in DMSO-*d*_6_ 15](#_Toc82601252)

[Figure S23. HSQC spectrum of compound **47** in DMSO-*d*_6_ 16](#_Toc82601253)

[Figure S24. HMBC spectrum of compound **47** in DMSO-*d*_6_ 16](#_Toc82601254)

[Figure S25. ^1^H - ^1^H COSY spectrum of compound **47** in DMSO-*d*_6_ 17](#_Toc82601255)

[Figure S26. NOESY spectrum of compound **47** in DMSO-*d*_6_ 17](#_Toc82601256)

[Figure S27. IR spectrum of compound **47** 18](#_Toc82601257)

[Figure S28. UV spectrum of compound **47** 18](#_Toc82601258)

[Figure S29. HR-ESI-MS of compound **6** 19](#_Toc82601259)

[Figure S30. ^1^H NMR spectrum of compound **6** in Methanol-*d_4_* 19](#_Toc82601260)

[Figure S31. ^13^C NMR spectrum of compound **6** in Methanol-*d_4_* 20](#_Toc82601261)

[Figure S32. HR-ESI-MS of compound **44** 20](#_Toc82601262)

[Figure S33. ^1^H NMR spectrum of compound **44** in DMSO-*d_6_* 21](#_Toc82601263)

[Figure S34. ^13^C NMR spectrum of compound **44** in DMSO-*d_6_* 21](#_Toc82601264)

[Figure S35. ^1^H NMR spectrum of compound **2** in DMSO-*d_6_* 22](#_Toc82601265)

[Figure S36. ^1^H NMR spectrum of compound **3** in Methanol-*d*_4_ 22](#_Toc82601266)

[Figure S37. ^13^C NMR spectrum of compound **3** in Methanol-*d*_4_ 23](#_Toc82601267)

[Figure S38. 1H NMR spectrum of compound 3 in Methanol-*d*_4_ 23](#_Toc82601268)

[Figure S39. ^13^C NMR spectrum of compound **4** in Methanol-*d*_4_ 24](#_Toc82601269)

[Figure S40. ^13^C NMR spectrum of compound **5** in DMSO-*d_6_* 24](#_Toc82601270)

[Figure S41. ^1^H NMR spectrum of compound **7** in DMSO-*d*_6_ 25](#_Toc82601271)

[Figure S42. ^13^C NMR spectrum of compound **7** in DMSO-*d*_6_ 25](#_Toc82601272)

[Figure S43. ^1^H NMR spectrum of compound **8** in DMSO-*d*_6_ 26](#_Toc82601273)

[Figure S44. ^1^H NMR spectrum of compound **9** in DMSO-*d*_6_ 26](#_Toc82601274)

[Figure S45. ^13^C NMR spectrum of compound **9** in DMSO-*d*_6_ 27](#_Toc82601275)

[Figure S46. ^1^H NMR spectrum of compound **10** in DMSO-*d*_6_ 27](#_Toc82601276)

[Figure S47. ^13^C NMR spectrum of compound **10** in DMSO-*d*_6_ 28](#_Toc82601277)

[Figure S48. ^1^H NMR spectrum of compound **11** in DMSO-*d*_6_ 28](#_Toc82601278)

[Figure S49. ^13^C NMR spectrum of compound **11** in DMSO-*d*_6_ 29](#_Toc82601279)

[Figure S50. ^1^H NMR spectrum of compound **12** in DMSO-*d*_6_ 29](#_Toc82601280)

[Figure S51. ^13^C NMR spectrum of compound **12** in DMSO-*d*_6_ 30](#_Toc82601281)

[Figure S52. ^1^H NMR spectrum of compound **13** in DMSO-*d*_6_ 30](#_Toc82601282)

[Figure S53. ^13^C NMR spectrum of compound **13** in DMSO-*d*_6_ 31](#_Toc82601283)

[Figure S54. ^1^H NMR spectrum of compound **14** in DMSO-*d*_6_ 31](#_Toc82601284)

[Figure S55. ^13^C NMR spectrum of compound **14** in DMSO-*d*_6_ 32](#_Toc82601285)

[Figure S56. ^1^H NMR spectrum of compound **15** in DMSO-*d*_6_ 32](#_Toc82601286)

[Figure S57. ^13^C NMR spectrum of compound **15** in DMSO-*d*_6_ 33](#_Toc82601287)

[Figure S58. ^1^H NMR spectrum of compound **16** in DMSO-*d*_6_ 33](#_Toc82601288)

[Figure S59. ^13^C NMR spectrum of compound **16** in DMSO-*d*_6_ 34](#_Toc82601289)

[Figure S60. ^1^H NMR spectrum of compound **17** in DMSO-*d*_6_ 34](#_Toc82601290)

[Figure S61. ^13^C NMR spectrum of compound **17** in DMSO-*d*_6_ 35](#_Toc82601291)

[Figure S62. ^1^H NMR spectrum of compound **18** in DMSO-*d*_6_ 35](#_Toc82601292)

[Figure S63. ^13^C NMR spectrum of compound **18** in DMSO-*d*_6_ 36](#_Toc82601293)

[Figure S64. ^1^H NMR spectrum of compound **19** in DMSO-*d*_6_ 36](#_Toc82601294)

[Figure S65. ^13^C NMR spectrum of compound **19** in DMSO-*d*_6_ 37](#_Toc82601295)

[Figure S66. ^1^H NMR spectrum of compound **20** in DMSO-*d*_6_ 37](#_Toc82601296)

[Figure S67. ^13^C NMR spectrum of compound **20** in DMSO-*d*_6_ 38](#_Toc82601297)

[Figure S68. ^1^H NMR spectrum of compound **21** in DMSO-*d*_6_ 38](#_Toc82601298)

[Figure S69. ^13^C NMR spectrum of compound **21** in DMSO-*d*_6_ 39](#_Toc82601299)

[Figure S70. ^1^H NMR spectrum of compound **22** in DMSO-*d*_6_ 39](#_Toc82601300)

[Figure S71. ^13^C NMR spectrum of compound **22** in DMSO-*d*_6_ 40](#_Toc82601301)

[Figure S72. ^1^H NMR spectrum of compound **23** in DMSO-*d*_6_ 40](#_Toc82601302)

[Figure S73. ^13^C NMR spectrum of compound **23** in DMSO-*d*_6_ 41](#_Toc82601303)

[Figure S74. ^1^H NMR spectrum of compound **24** in DMSO-*d*_6_ 41](#_Toc82601304)

[Figure S75. ^13^C NMR spectrum of compound **24** in DMSO-*d*_6_ 42](#_Toc82601305)

[Figure S76. ^1^H NMR spectrum of compound **25** in DMSO-*d*_6_ 42](#_Toc82601306)

[Figure S77. ^13^C NMR spectrum of compound **25** in DMSO-*d*_6_ 43](#_Toc82601307)

[Figure S78. ^1^H NMR spectrum of compound **26** in DMSO-*d*_6_ 43](#_Toc82601308)

[Figure S79. ^13^C NMR spectrum of compound **26** in DMSO-*d*_6_ 44](#_Toc82601309)

[Figure S80. ^1^H NMR spectrum of compound **27** in DMSO-*d*_6_ 44](#_Toc82601310)

[Figure S81. ^13^C NMR spectrum of compound **27** in DMSO-*d*_6_ 45](#_Toc82601311)

[Figure S82. ^1^H NMR spectrum of compound **28** in DMSO-*d*_6_ 45](#_Toc82601312)

[Figure S83. ^13^C NMR spectrum of compound **28** in DMSO-*d*_6_ 46](#_Toc82601313)

[Figure S84. ^1^H NMR spectrum of compound **29** in DMSO-*d*_6_ 46](#_Toc82601314)

[Figure S85. ^13^C NMR spectrum of compound **29** in DMSO-*d*_6_ 47](#_Toc82601315)

[Figure S86. ^1^H NMR spectrum of compound **30** in DMSO-*d*_6_ 47](#_Toc82601316)

[Figure S87. ^13^C NMR spectrum of compound **30** in DMSO-*d*_6_ 48](#_Toc82601317)

[Figure S88. ^1^H NMR spectrum of compound **31** in DMSO-*d*_6_ 48](#_Toc82601318)

[Figure S89. ^13^C NMR spectrum of compound **31** in DMSO-*d*_6_ 49](#_Toc82601319)

[Figure S90. ^1^H NMR spectrum of compound **32** in DMSO-*d*_6_ 49](#_Toc82601320)

[Figure S91. ^13^C NMR spectrum of compound **32** in DMSO-*d*_6_ 50](#_Toc82601321)

[Figure S92. ^1^H NMR spectrum of compound **33** in DMSO-*d*_6_ 50](#_Toc82601322)

[Figure S93. ^13^C NMR spectrum of compound **33** in DMSO-*d*_6_ 51](#_Toc82601323)

[Figure S94. ^1^H NMR spectrum of compound **34** in DMSO-*d*_6_ 51](#_Toc82601324)

[Figure S95. ^13^C NMR spectrum of compound **34** in DMSO-*d*_6_ 52](#_Toc82601325)

[Figure S96. ^1^H NMR spectrum of compound **35** in DMSO-*d*_6_ 52](#_Toc82601326)

[Figure S97. ^13^C NMR spectrum of compound **35** in DMSO-*d*_6_ 53](#_Toc82601327)

[Figure S98. ^1^H NMR spectrum of compound **36** in DMSO-*d*_6_ 53](#_Toc82601328)

[Figure S99. ^13^C NMR spectrum of compound **36** in DMSO-*d*_6_ 54](#_Toc82601329)

[Figure S100. ^1^H NMR spectrum of compound **37** in DMSO-*d*_6_ 54](#_Toc82601330)

[Figure S101. ^13^C NMR spectrum of compound **37** in DMSO-*d*_6_ 55](#_Toc82601331)

[Figure S102. ^1^H NMR spectrum of compound **38** in Methanol-*d*_4_ 55](#_Toc82601332)

[Figure S103. ^13^C NMR spectrum of compound **38** in Methanol-*d*_4_ 56](#_Toc82601333)

[Figure S104. ^1^H NMR spectrum of compound **39** in Methanol-*d*_4_ 56](#_Toc82601334)

[Figure S105. ^1^H NMR spectrum of compound **40** in Methanol-*d*_4_ 57](#_Toc82601335)

[Figure S106. ^13^C NMR spectrum of compound **40** in Methanol-*d*_4_ 57](#_Toc82601336)

[Figure S107. ^1^H NMR spectrum of compound **41** in Methanol-*d*_4_ 58](#_Toc82601337)

[Figure S108. ^13^C NMR spectrum of compound **41** in Methanol-*d*_4_ 58](#_Toc82601338)

[Figure S109. ^1^H NMR spectrum of compound **42** in DMSO-*d*_6_ 59](#_Toc82601339)

[Figure S110. ^13^C NMR spectrum of compound **42** in DMSO-*d*_6_ 59](#_Toc82601340)

[Figure S111. ^1^H NMR spectrum of compound **43** in DMSO-*d*_6_ 60](#_Toc82601341)

[Figure S112. ^13^C NMR spectrum of compound **43** in DMSO-*d*_6_ 60](#_Toc82601342)

[Figure S113. ^1^H NMR spectrum of compound **45** in DMSO-*d*_6_ 61](#_Toc82601343)

[Figure S114. ^13^C NMR spectrum of compound **45** in DMSO-*d*_6_ 61](#_Toc82601344)

[Figure S115. ^1^H NMR spectrum of compound **48** in DMSO-*d*_6_ 62](#_Toc82601345)

[Figure S116. ^13^C NMR spectrum of compound **48** in DMSO-*d*_6_ 62](#_Toc82601346)

[Figure S117. ^1^H NMR spectrum of compound **49** in DMSO-*d*_6_ 63](#_Toc82601347)

[Figure S118. ^13^C NMR spectrum of compound **49** in DMSO-*d*_6_ 63](#_Toc82601348)

[Figure S119. ^1^H NMR spectrum of compound **50** in Methanol-*d*_4_ 64](#_Toc82601349)

[Figure S120. ^13^C NMR spectrum of compound **50** in Methanol-*d*_4_ 64](#_Toc82601350)

[Figure S121. GO enrichment analysis of the candidate targets 65](#_Toc82601351)

[Figure S122. KEGG pathway enrichment analysis of the candidate targets 65](#_Toc82601352)

[Figure S123. The correlation between PTP1B IC_50_ values and Stern-Volmer constants (K_sv_) of compounds **1**, **6**, **30**, **44** and **45** 66](#_Toc82601353)

[Table S1. Fifteen key targets of the fruits of Livistona chinensis against diabetes identified with network analyzer 66](#_Toc82601354)

[**UPLC-Q-TOF Parameters** 66](#_Toc82601355)

[**The identification methodologies of known compounds** 67](#_Toc82601356)


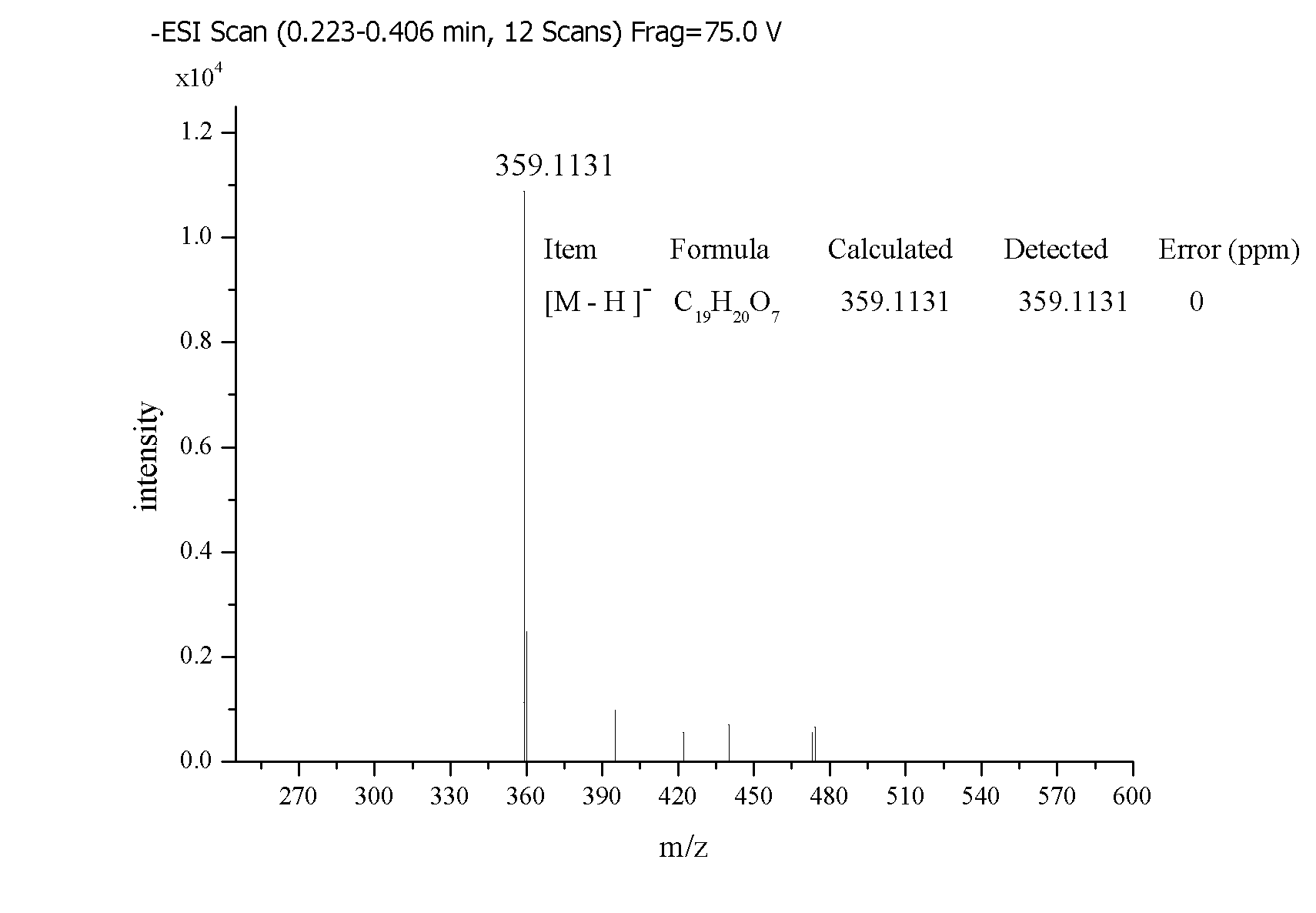


# Figure S1. HR-ESI-MS spectrum of compound **1**


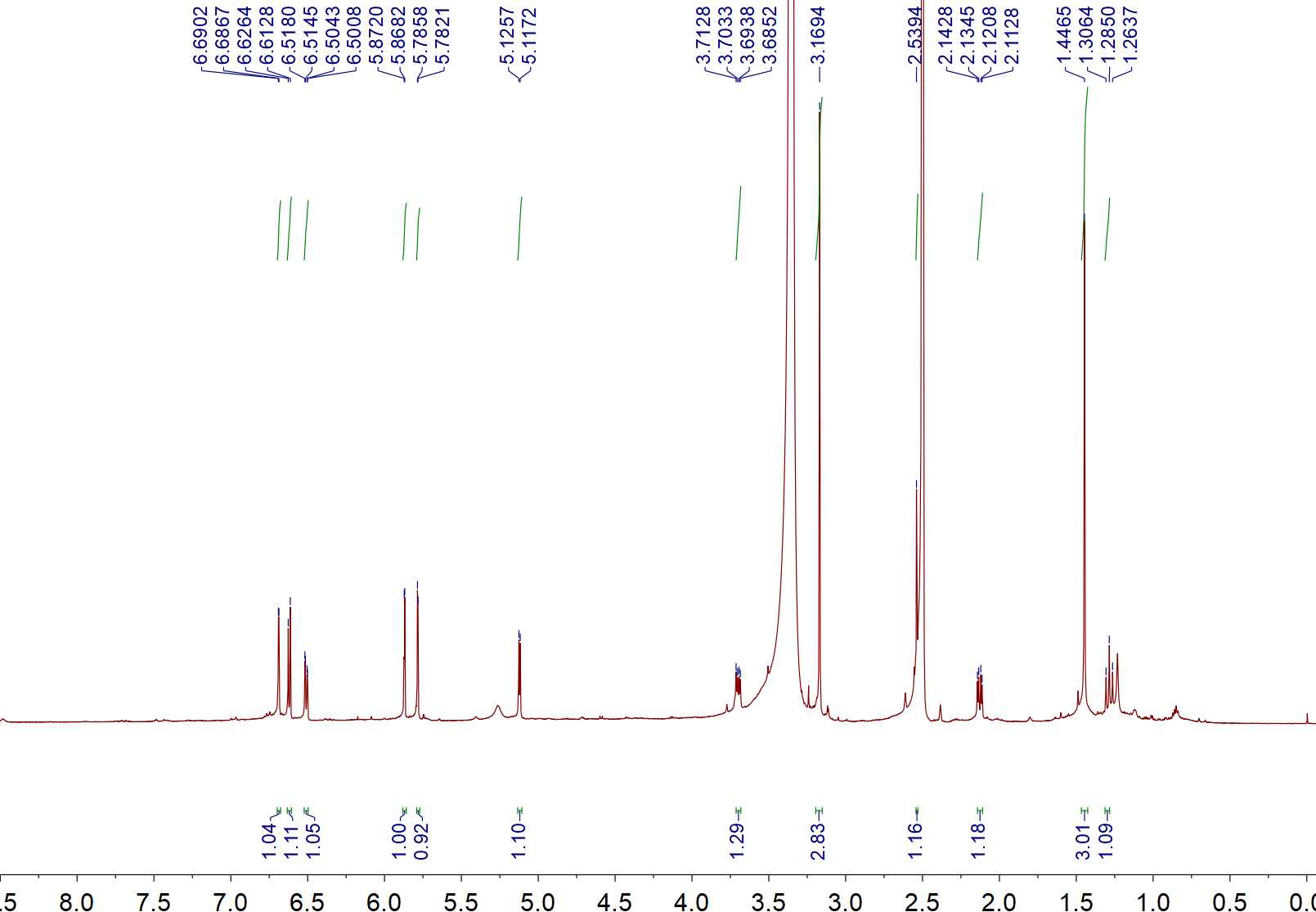


# Figure S2. ^1^H NMR spectrum of compound **1** in DMSO-*d*_6_


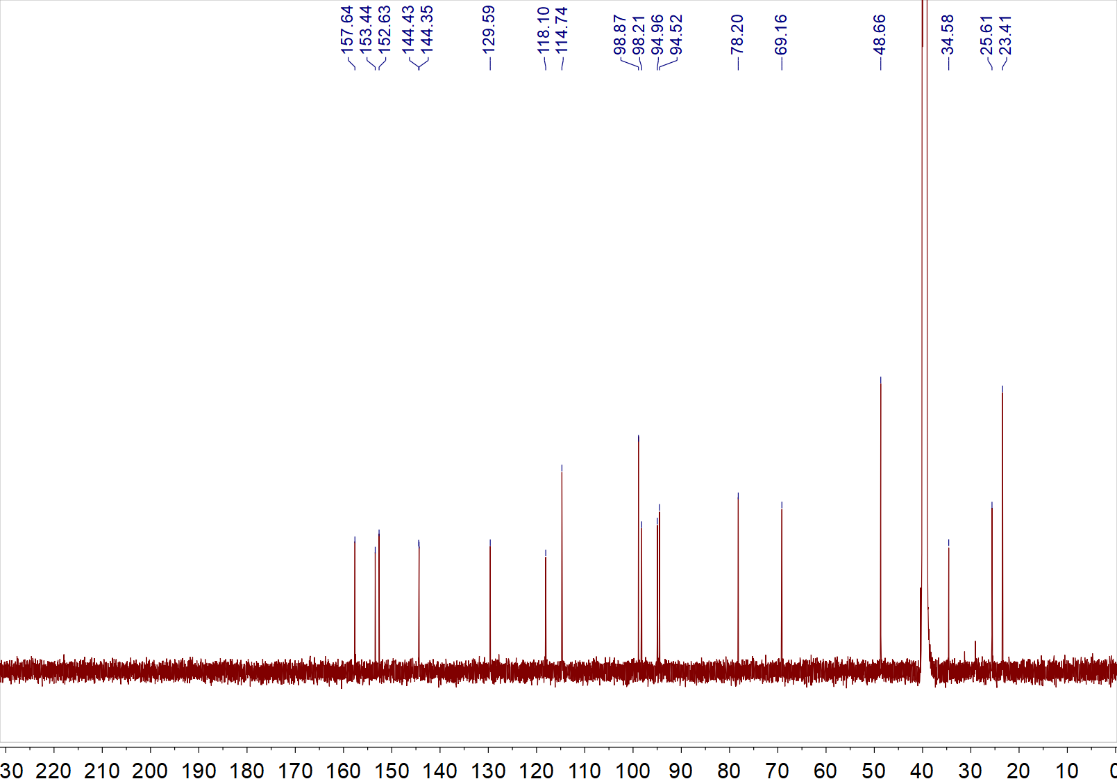


# Figure S3. ^13^C NMR spectrum of compound **1** in DMSO-*d*_6_


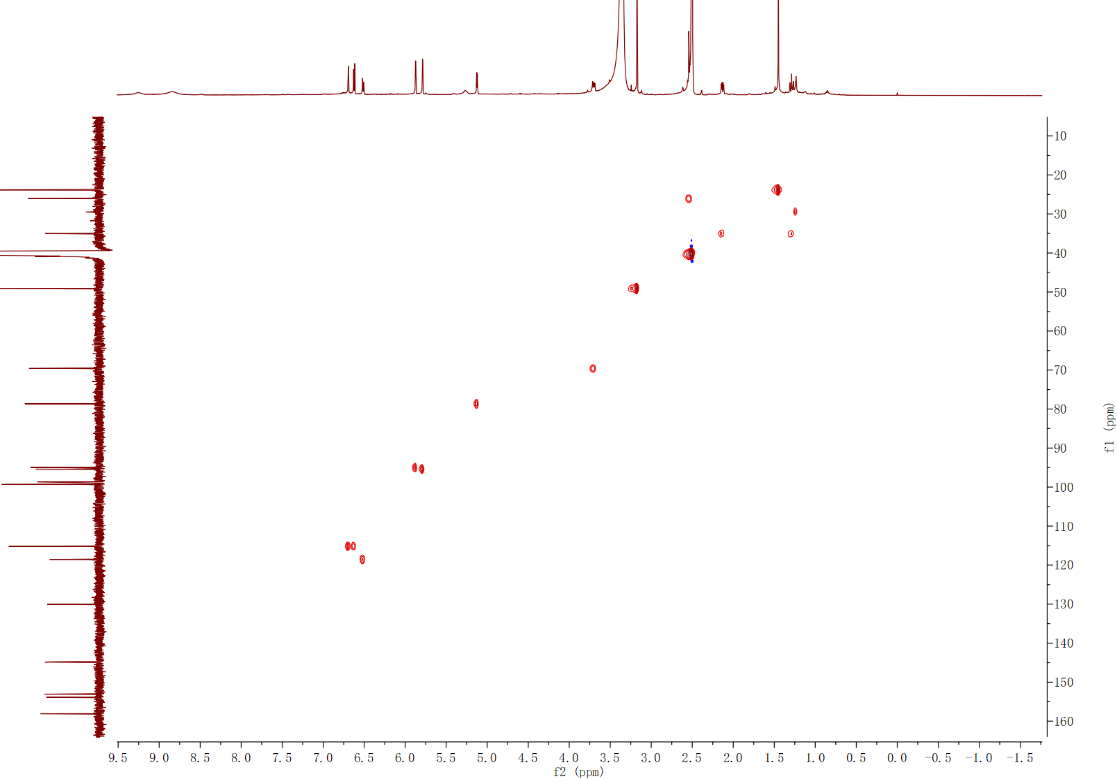


# Figure S4. HSQC spectrum of compound **1** in DMSO-*d*_6_


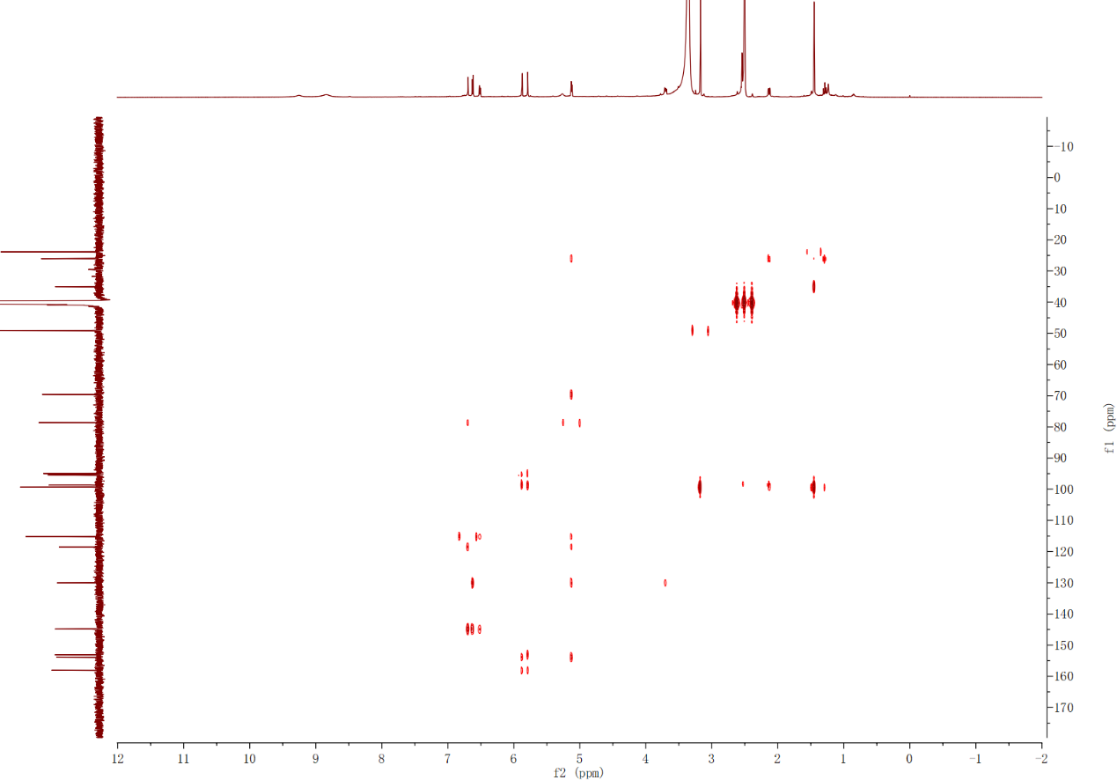


# Figure S5. HMBC spectrum of compound **1** in DMSO-*d*_6_


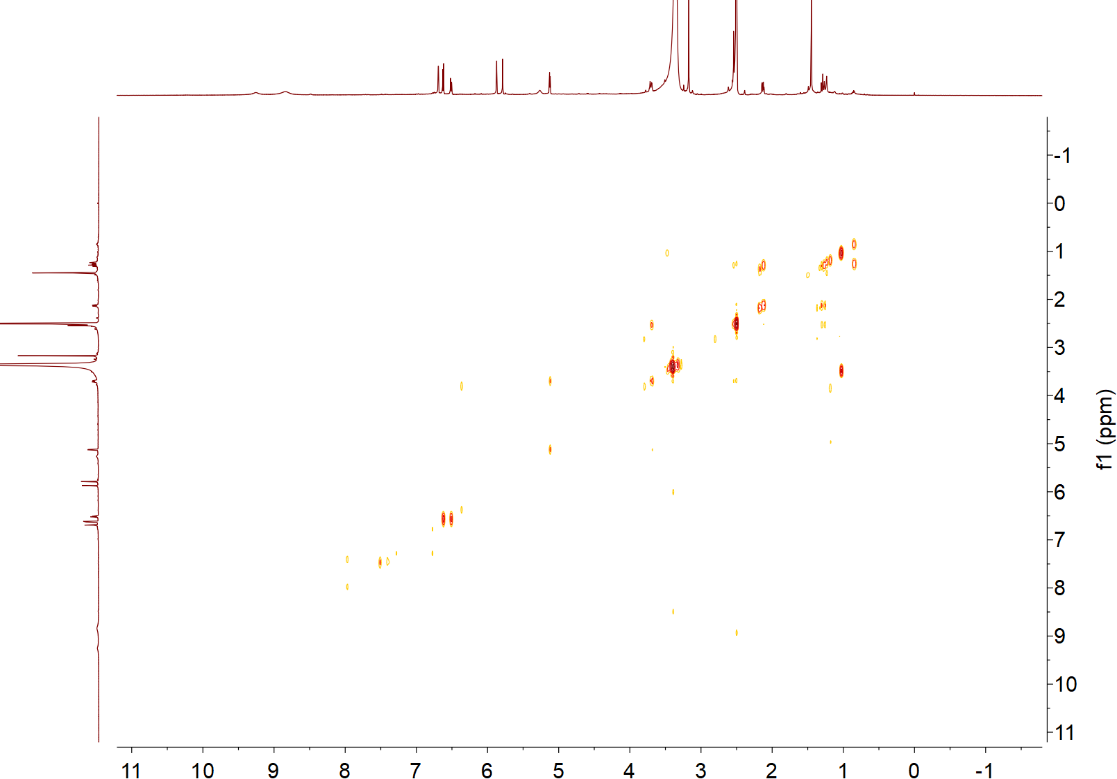


# Figure S6. ^1^H – ^1^H COSY spectrum of compound **1** in DMSO-*d*_6_


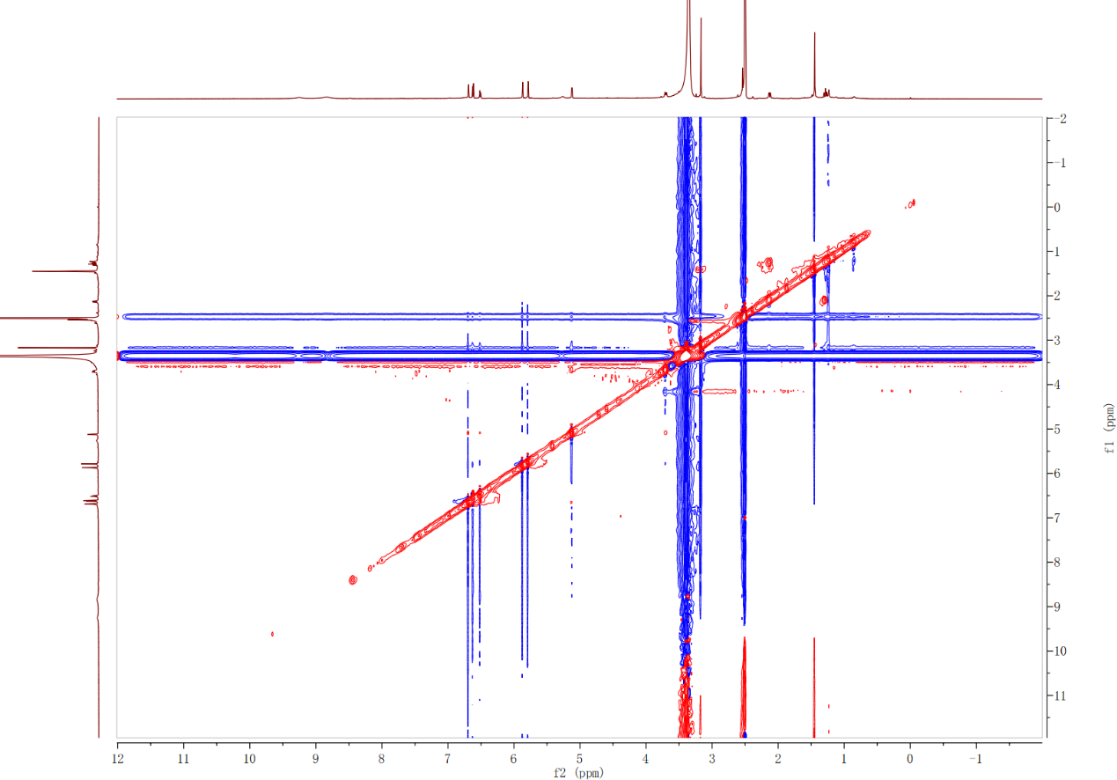


# Figure S7. NOESY spectrum of compound **1** in DMSO-*d*_6_


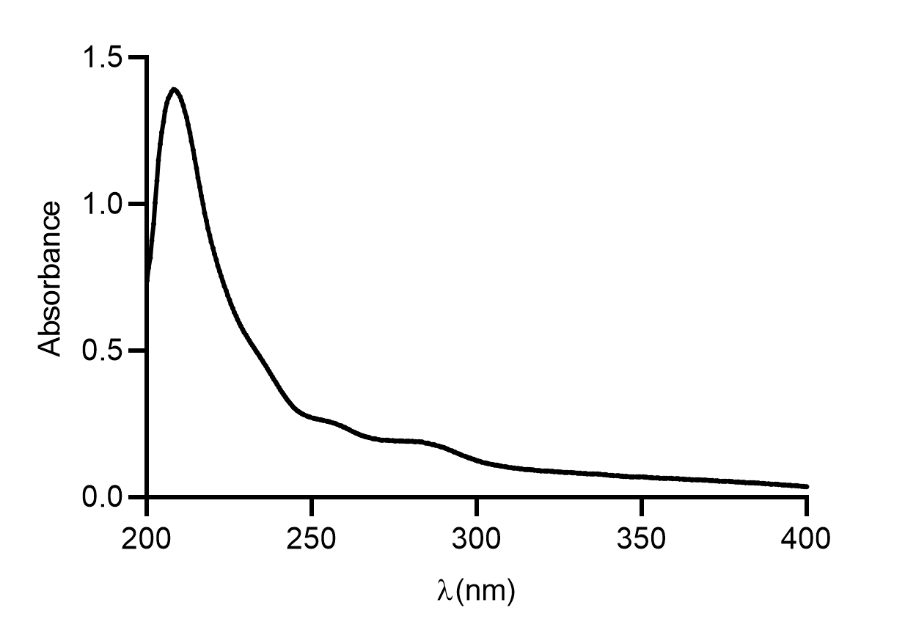


# Figure S8. UV spectrum of compound **1**


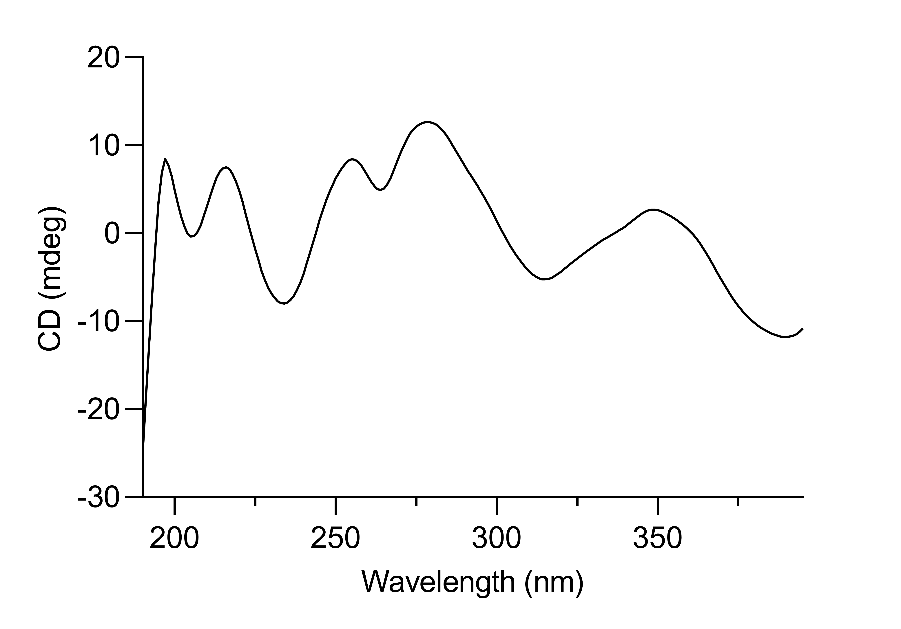


# Figure S9. CD spectrum of compound **1**


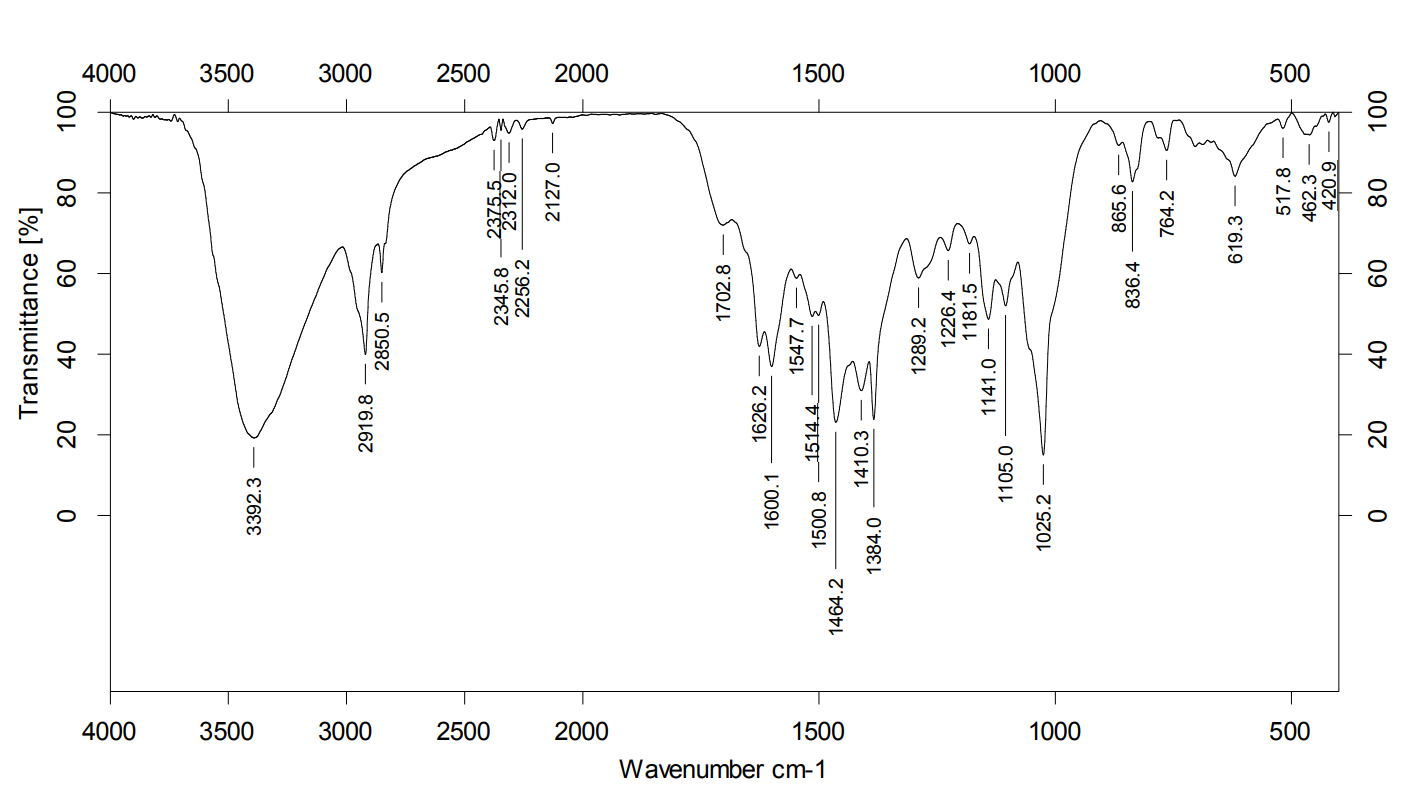


# Figure S10. IR spectrum of compound **1**


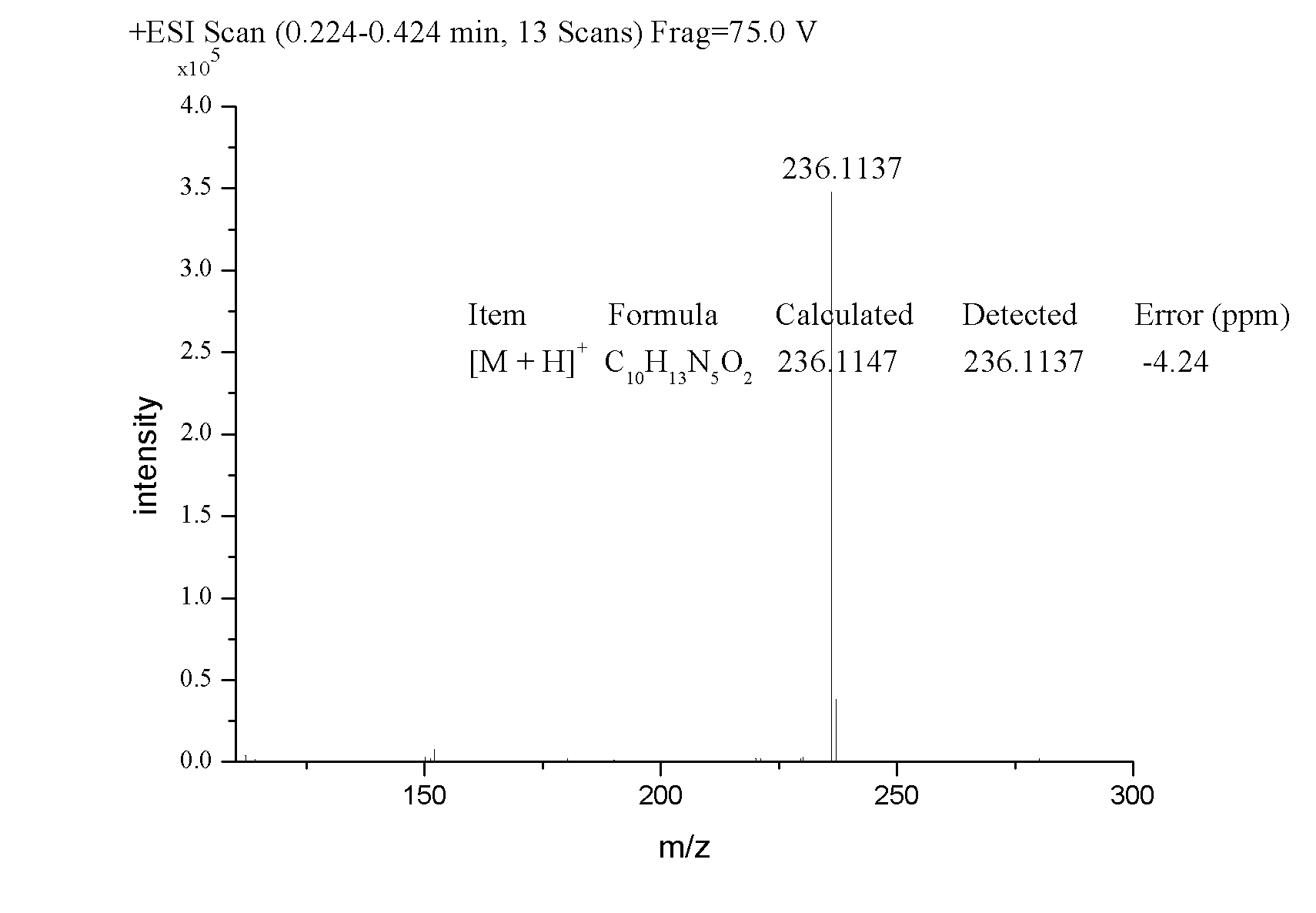


# Figure S11. HR-ESI-MS spectrum of compound **46**


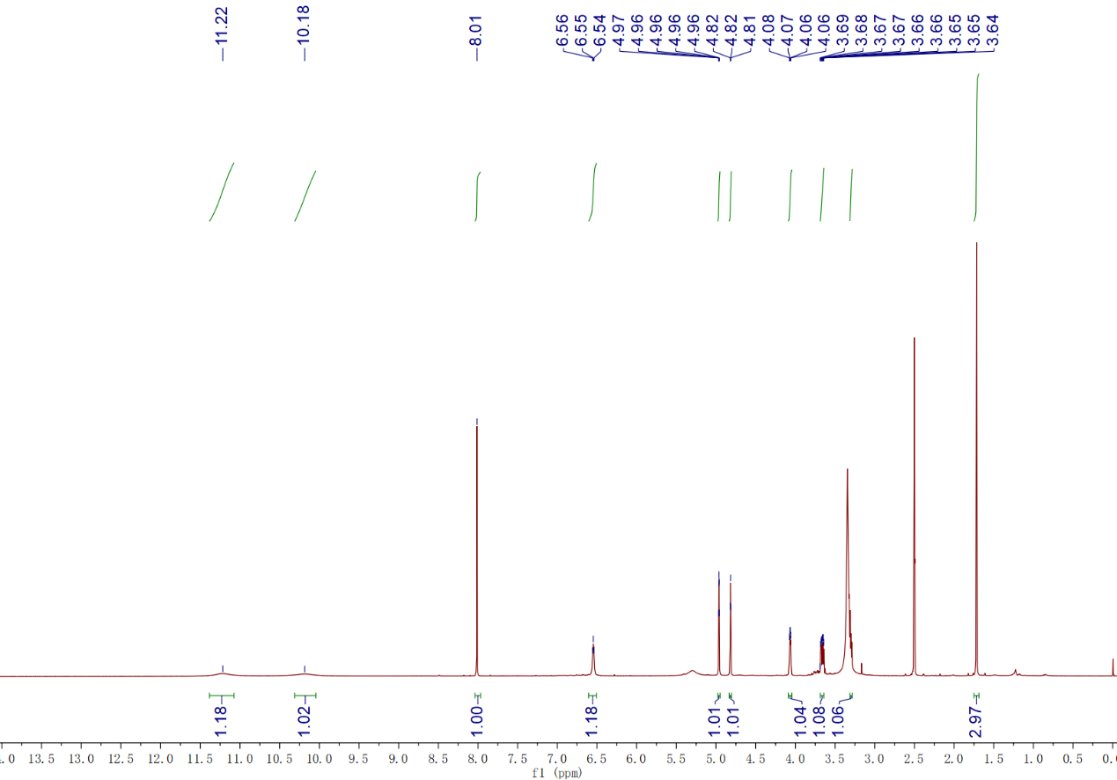


# Figure S12. ^1^H NMR spectrum of compound **46** in DMSO-*d*_6_


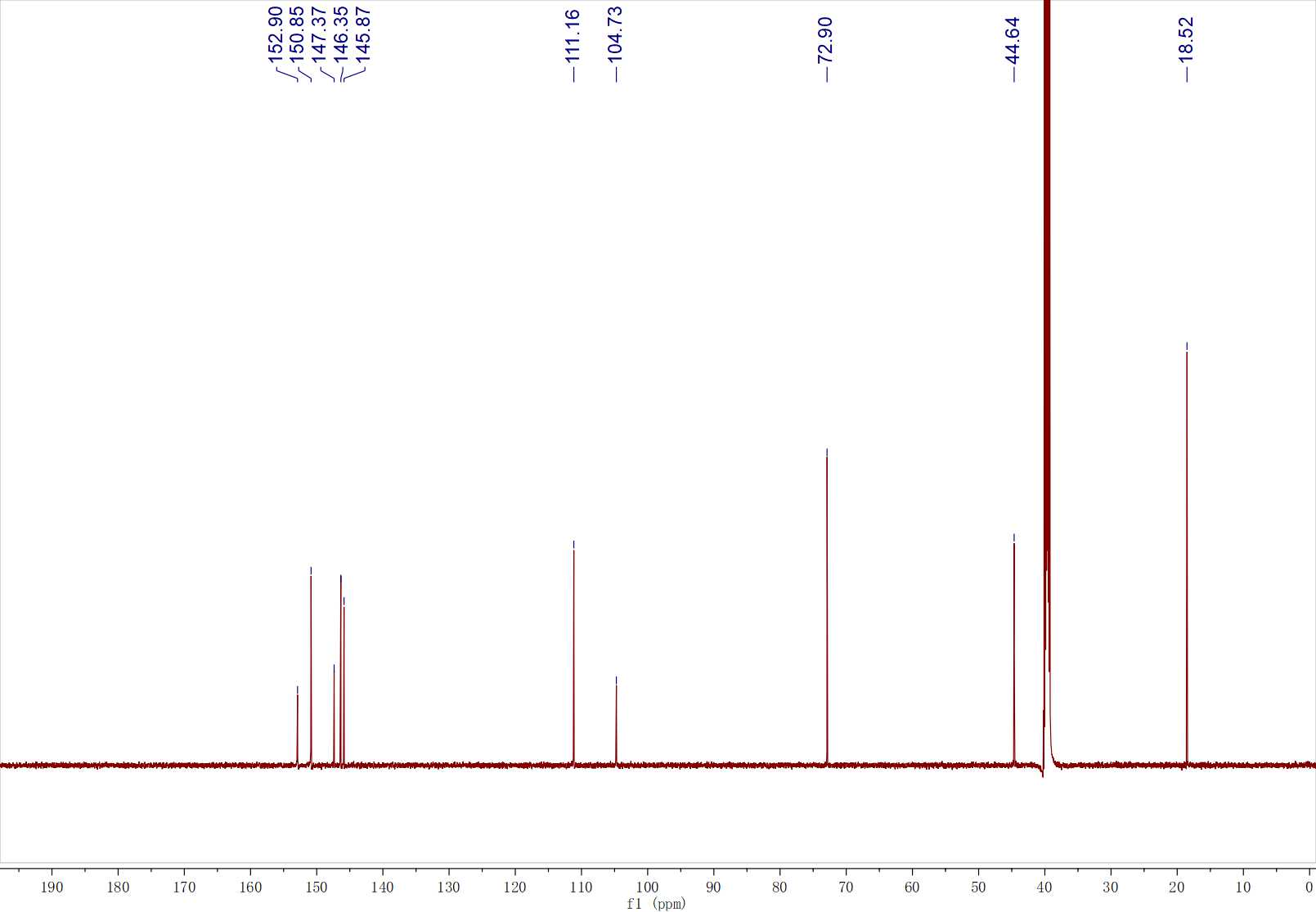


# Figure S13. ^13^C NMR spectrum of compound **46** in DMSO-*d*_6_


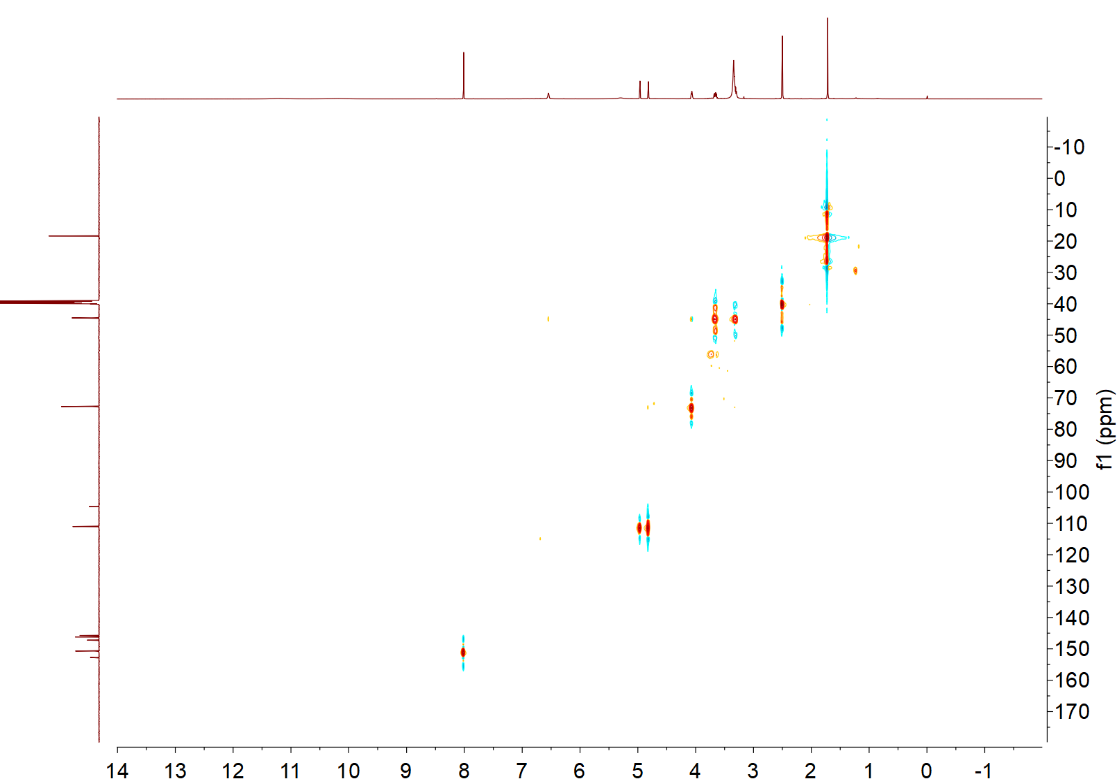


# Figure S14. HSQC spectrum of compound **46** in DMSO-*d*_6_


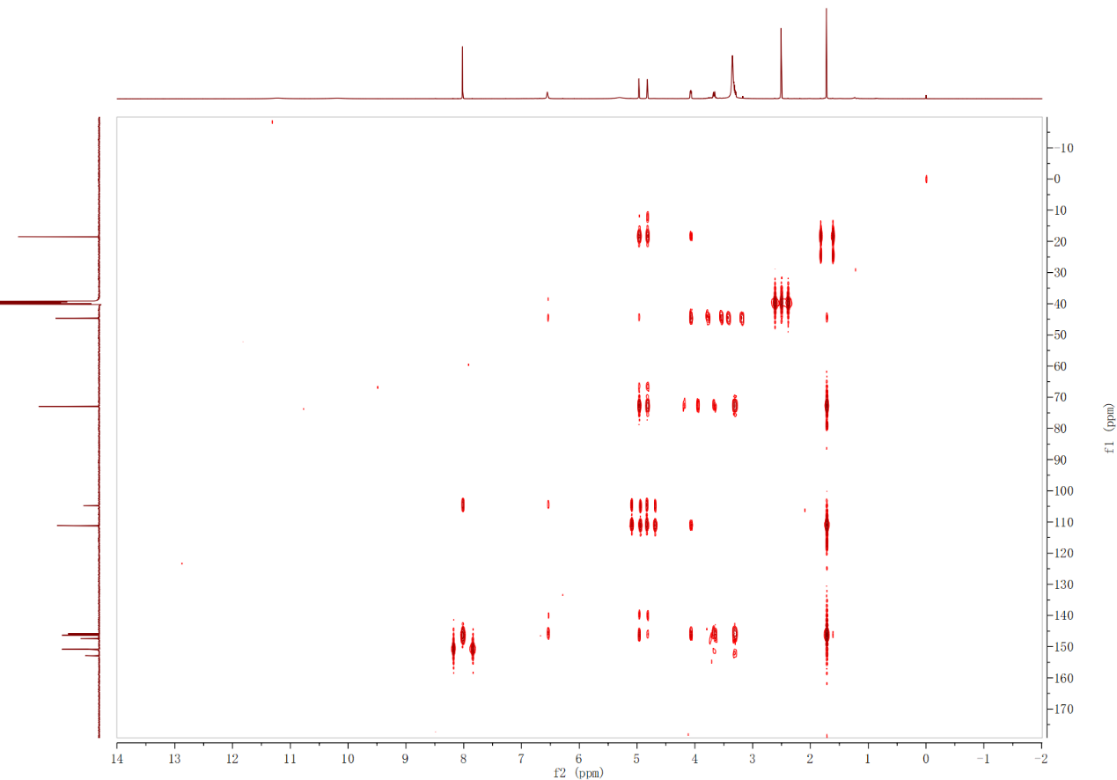


# Figure S15. HMBC spectrum of compound **46** in DMSO-*d*_6_


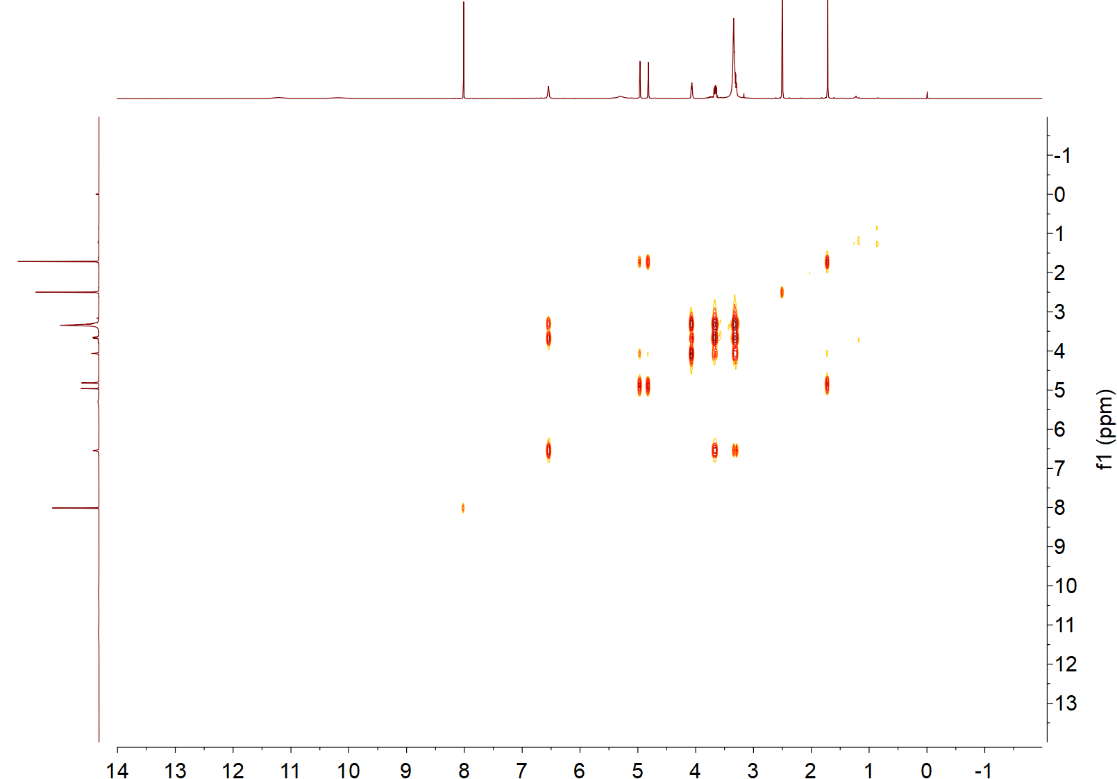


# Figure S16. ^1^H – ^1^H COSY spectrum of compound **46** in DMSO-*d*_6_


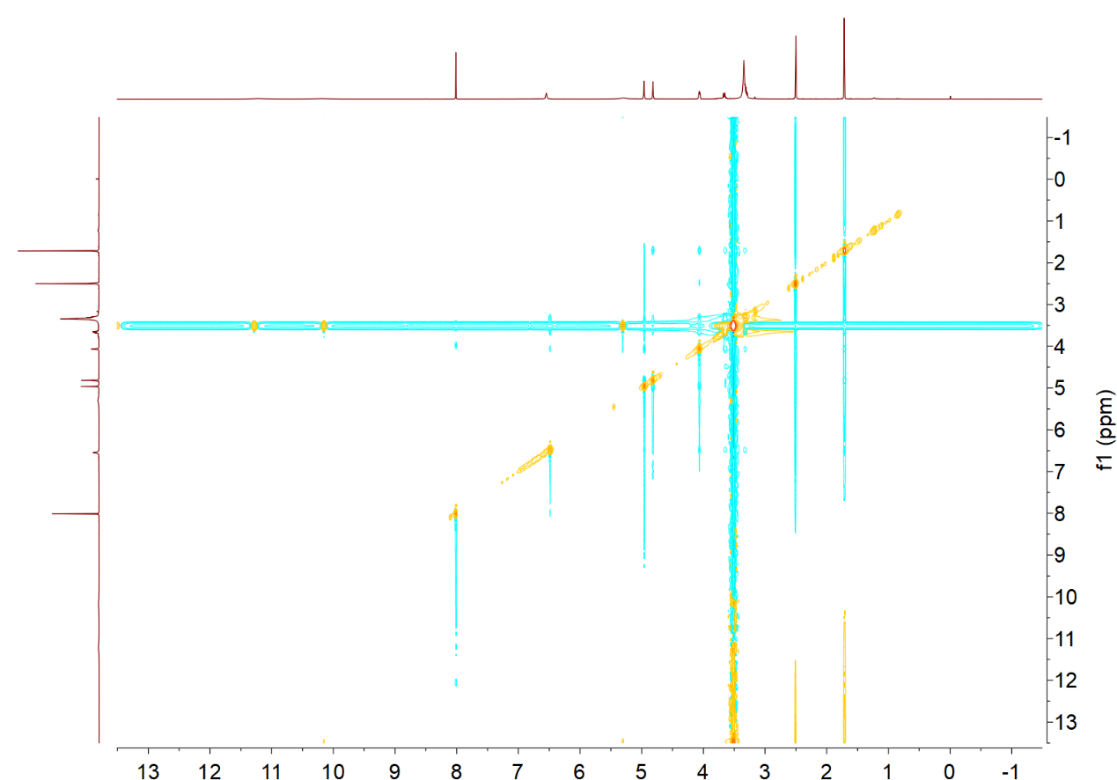


# Figure S17. NOESY spectrum of compound **46** in DMSO-*d*_6_


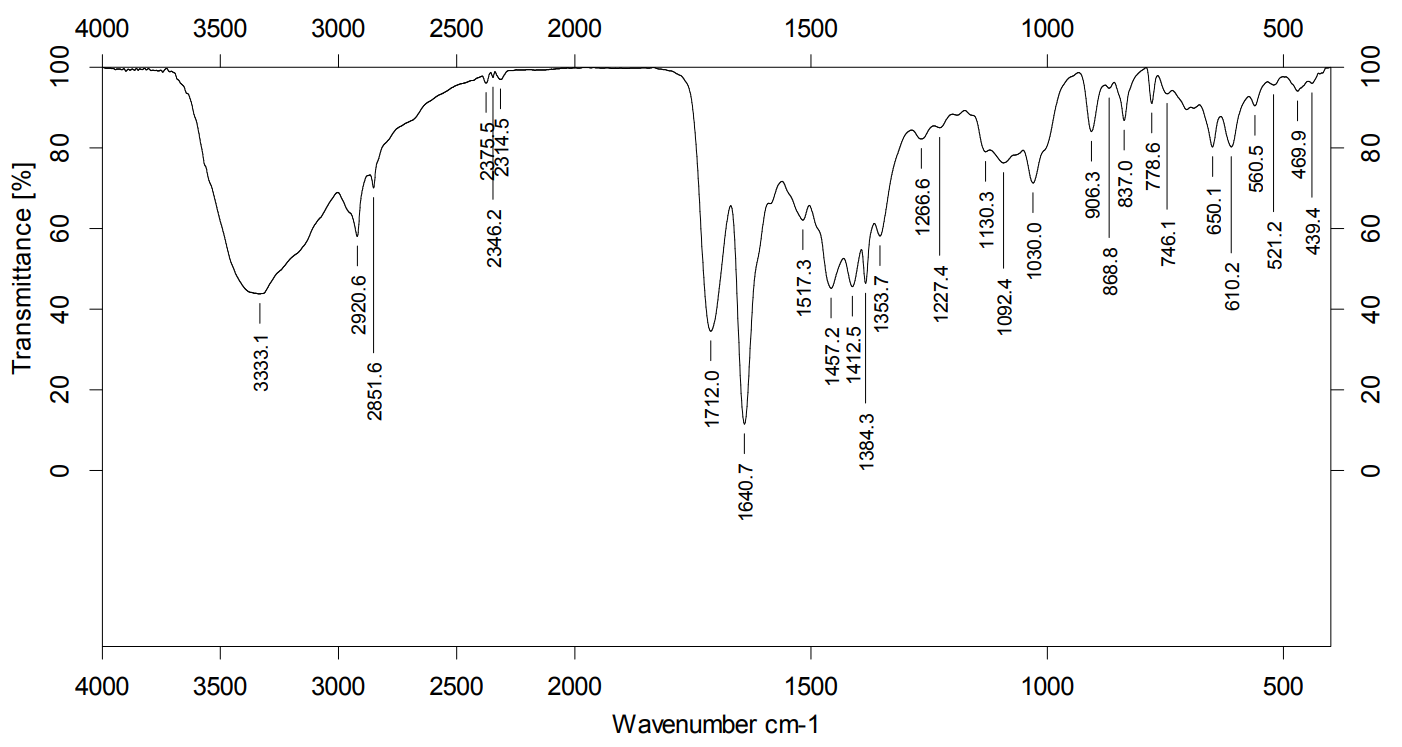


# Figure S18. IR spectrum of compound **46**


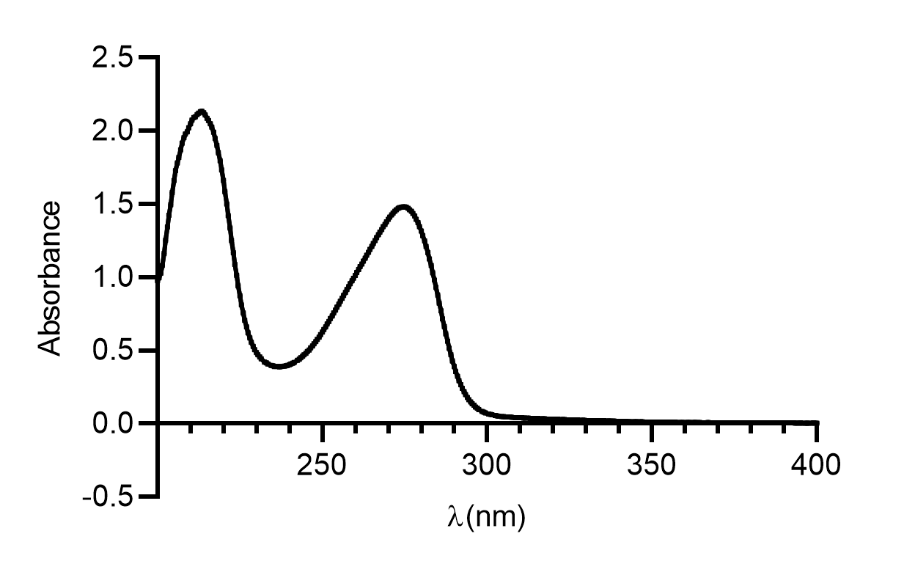


# Figure S19. UV spectrum of compound **46**


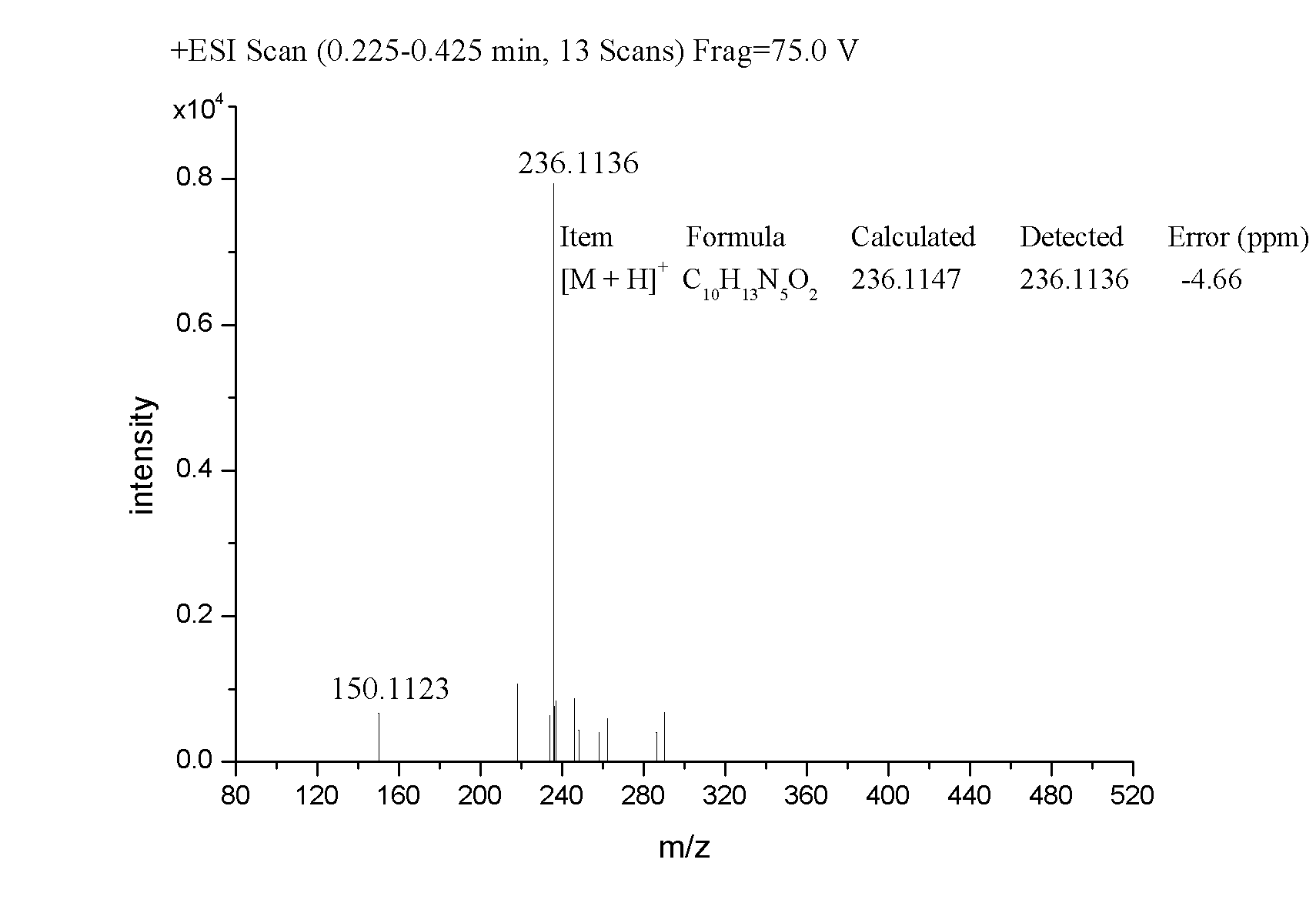


# Figure S20. HR-ESI-MS spectrum of compound **47**


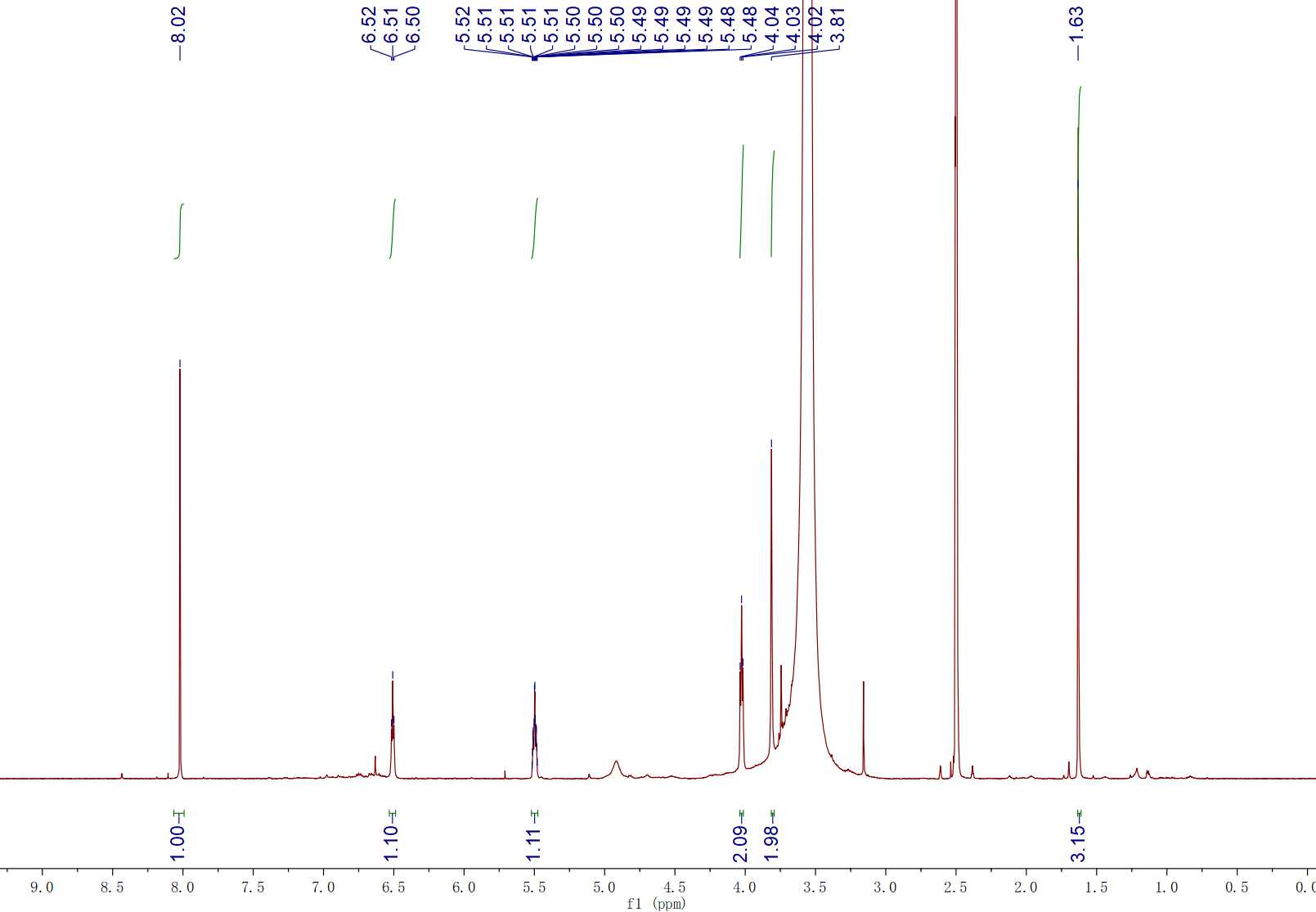


# Figure S21. ^1^H NMR spectrum of compound **47** in DMSO-*d*_6_


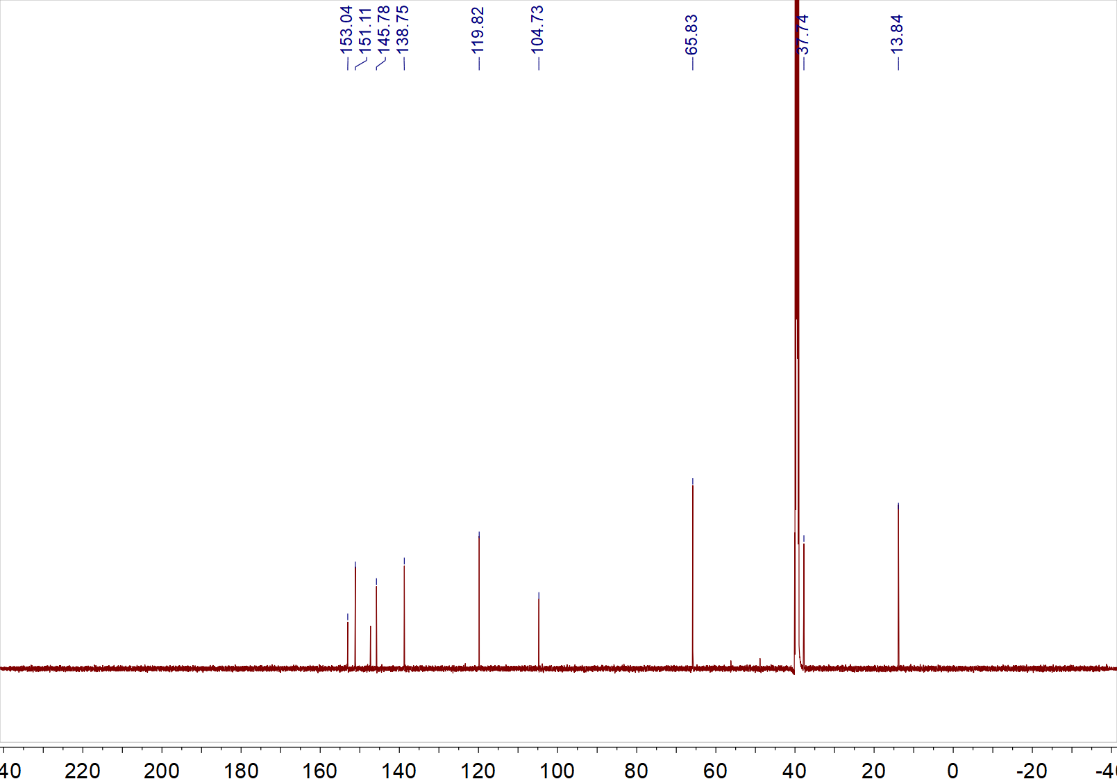


# Figure S22. ^13^C NMR spectrum of compound **47** in DMSO-*d*_6_


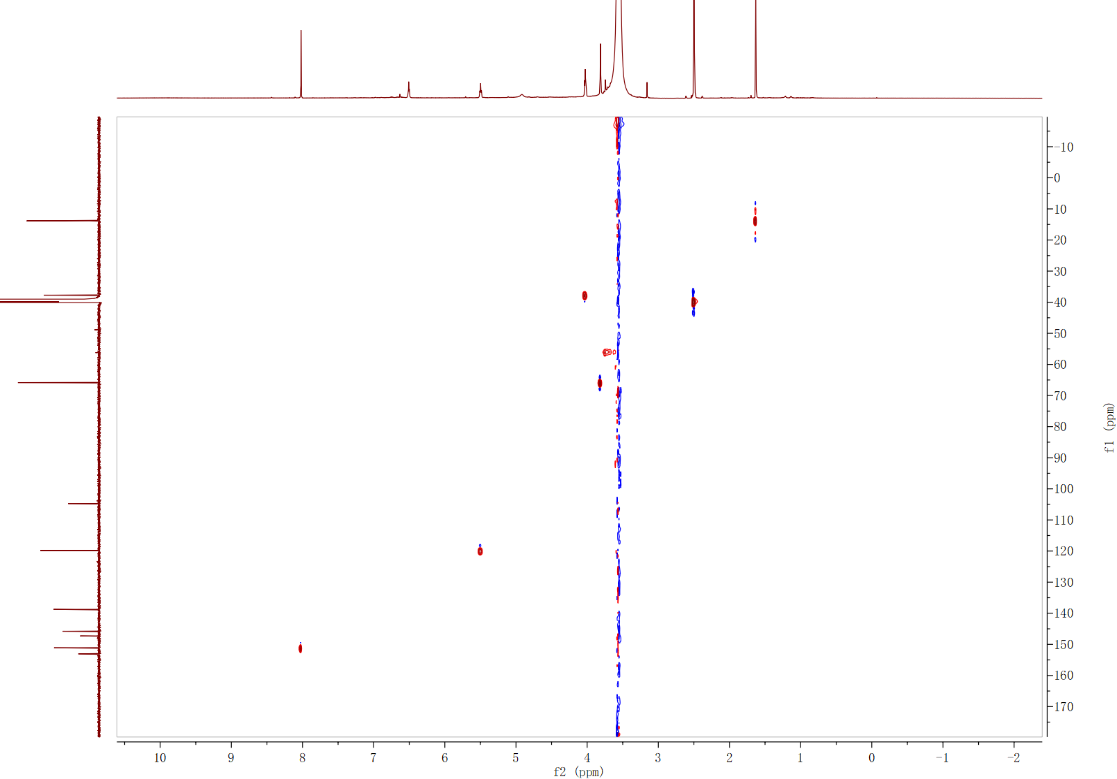


# Figure S23. HSQC spectrum of compound **47** in DMSO-*d*_6_


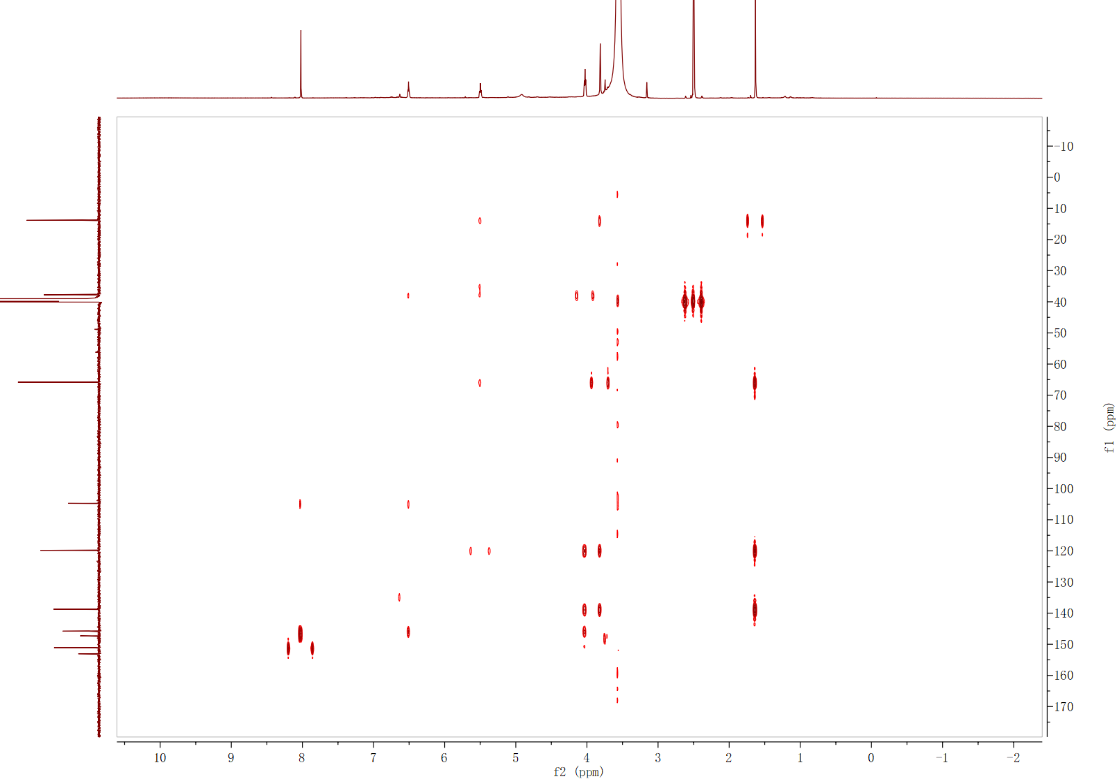


# Figure S24. HMBC spectrum of compound **47** in DMSO-*d*_6_


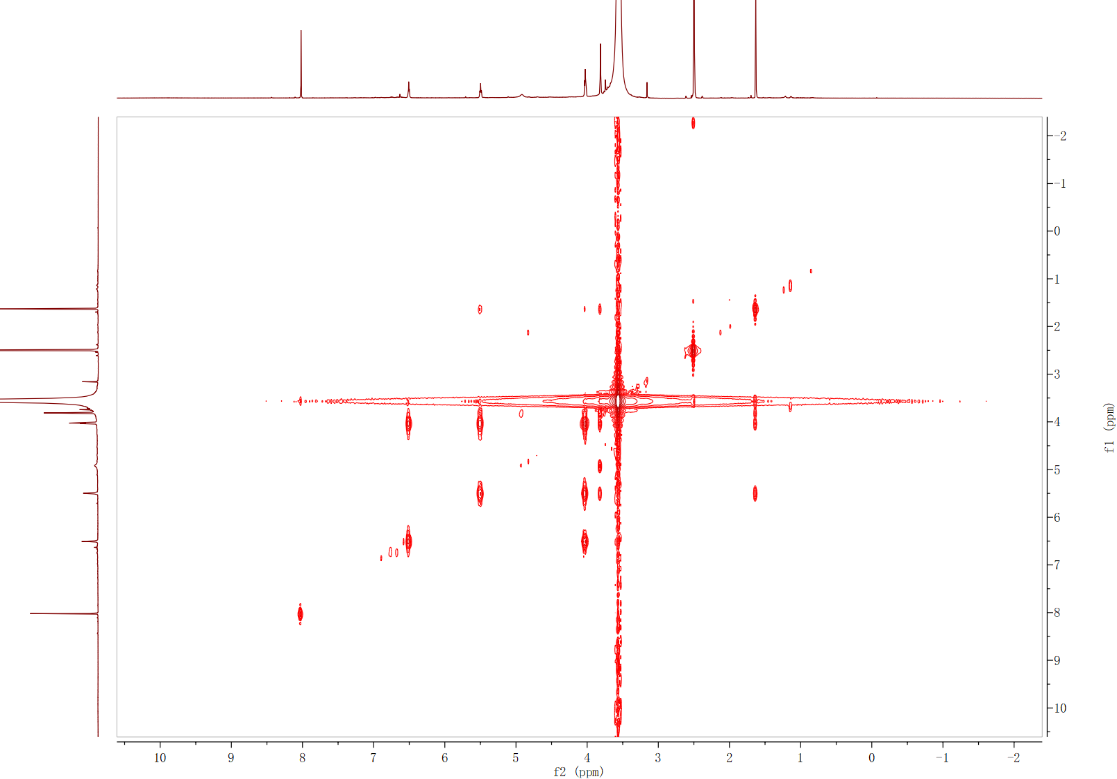


# Figure S25. ^1^H - ^1^H COSY spectrum of compound **47** in DMSO-*d*_6_


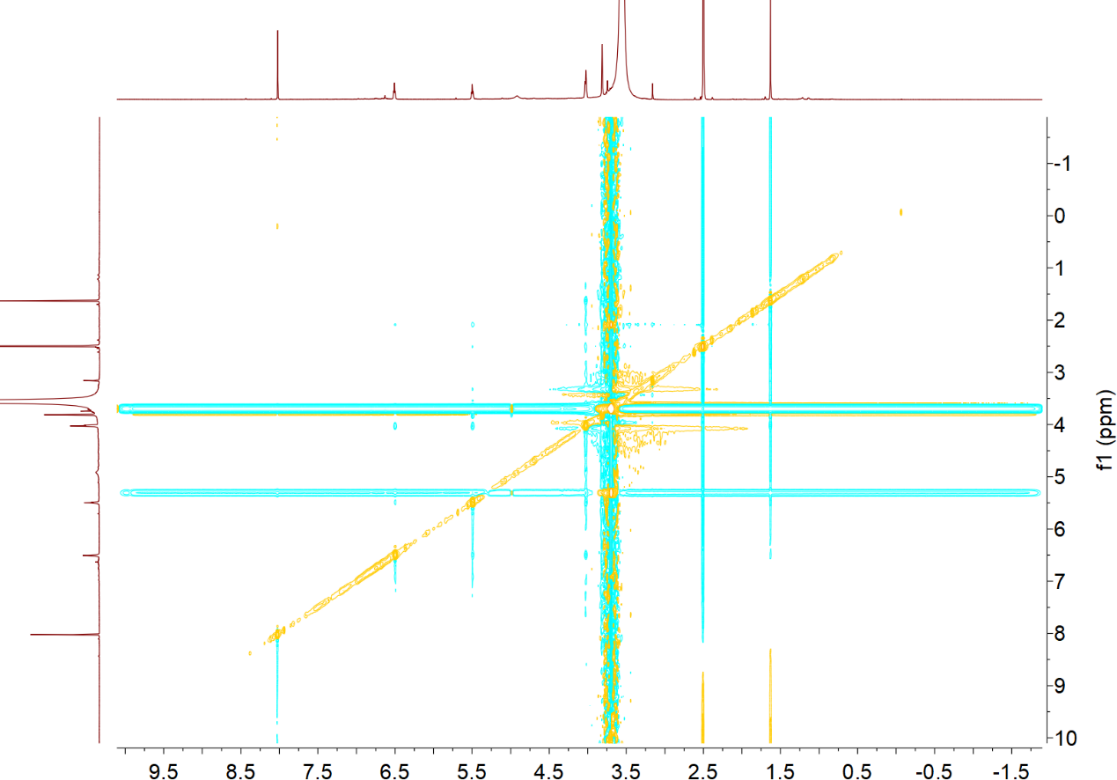


# Figure S26. NOESY spectrum of compound **47** in DMSO-*d*_6_


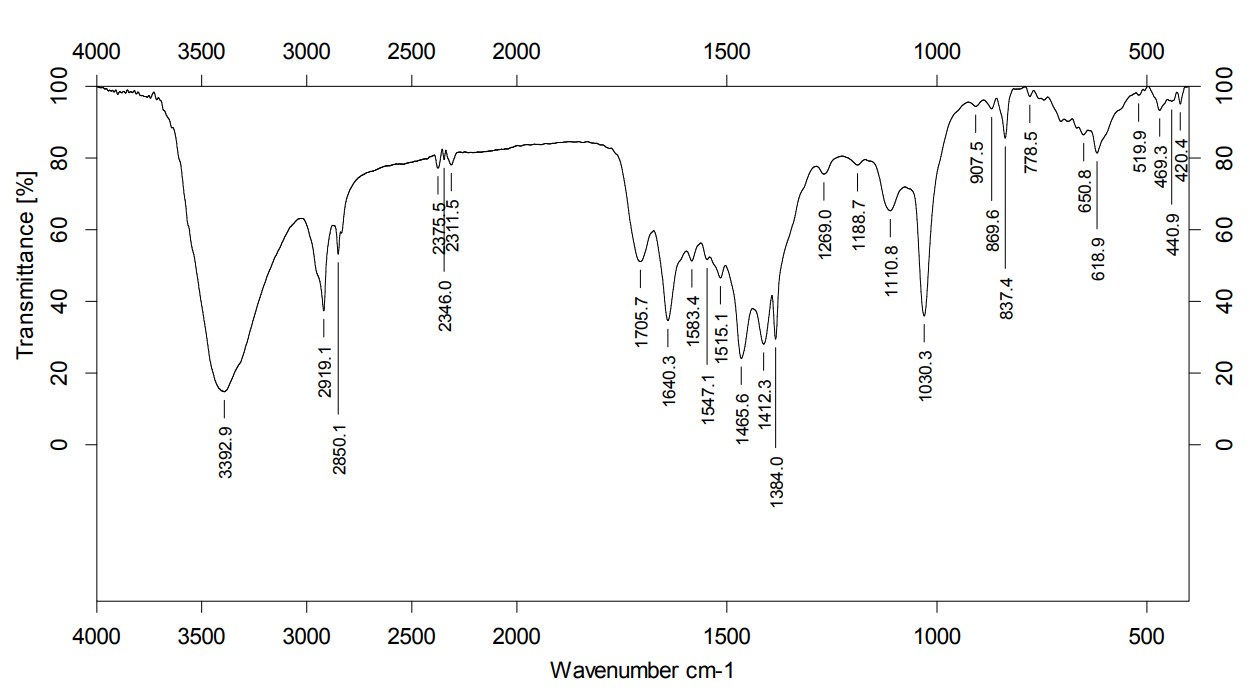


# Figure S27. IR spectrum of compound **47**


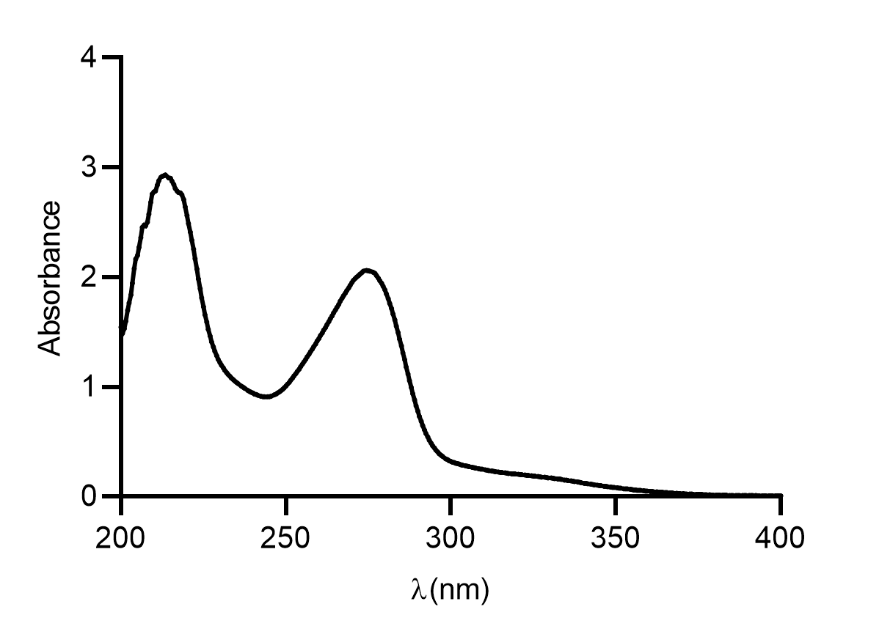


# Figure S28. UV spectrum of compound **47**

8.5


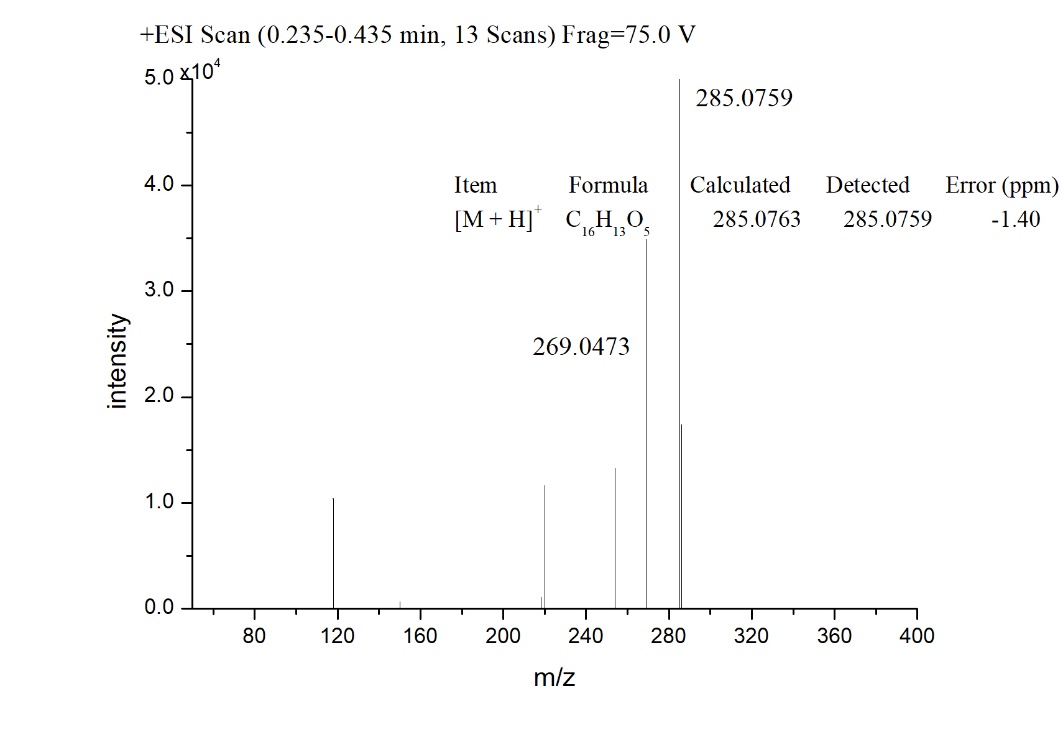


# Figure S29. HR-ESI-MS of compound **6**


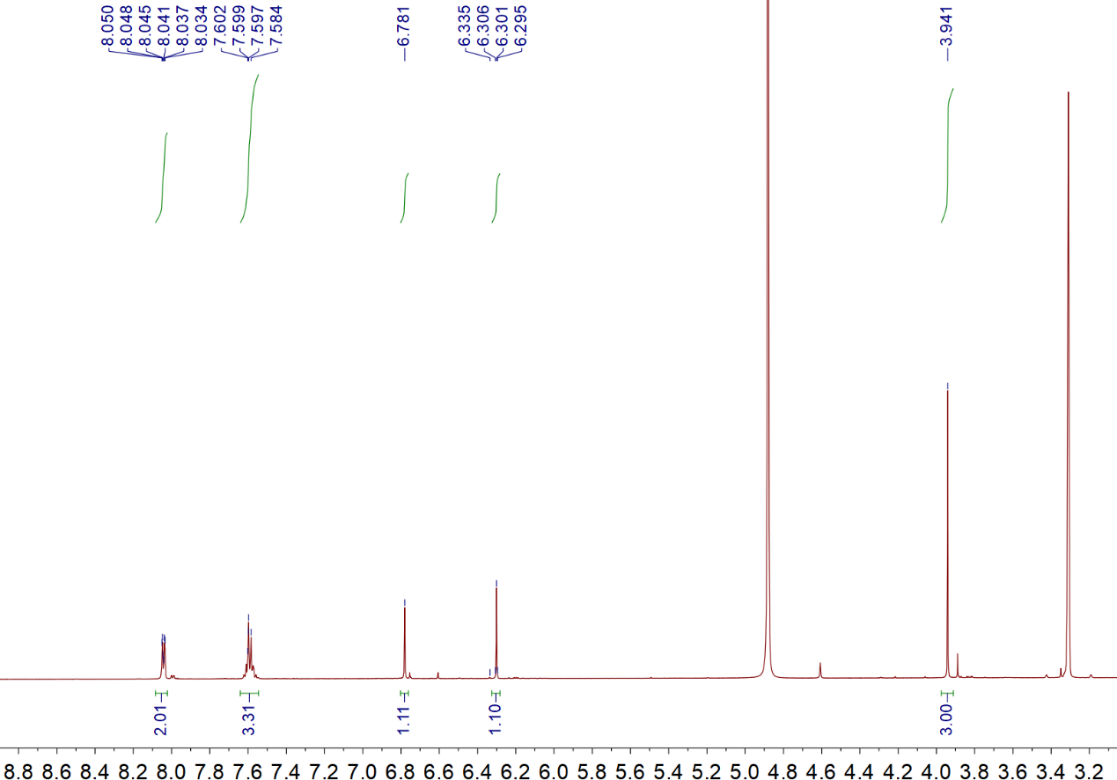


# Figure S30. ^1^H NMR spectrum of compound **6** in Methanol-*d_4_*


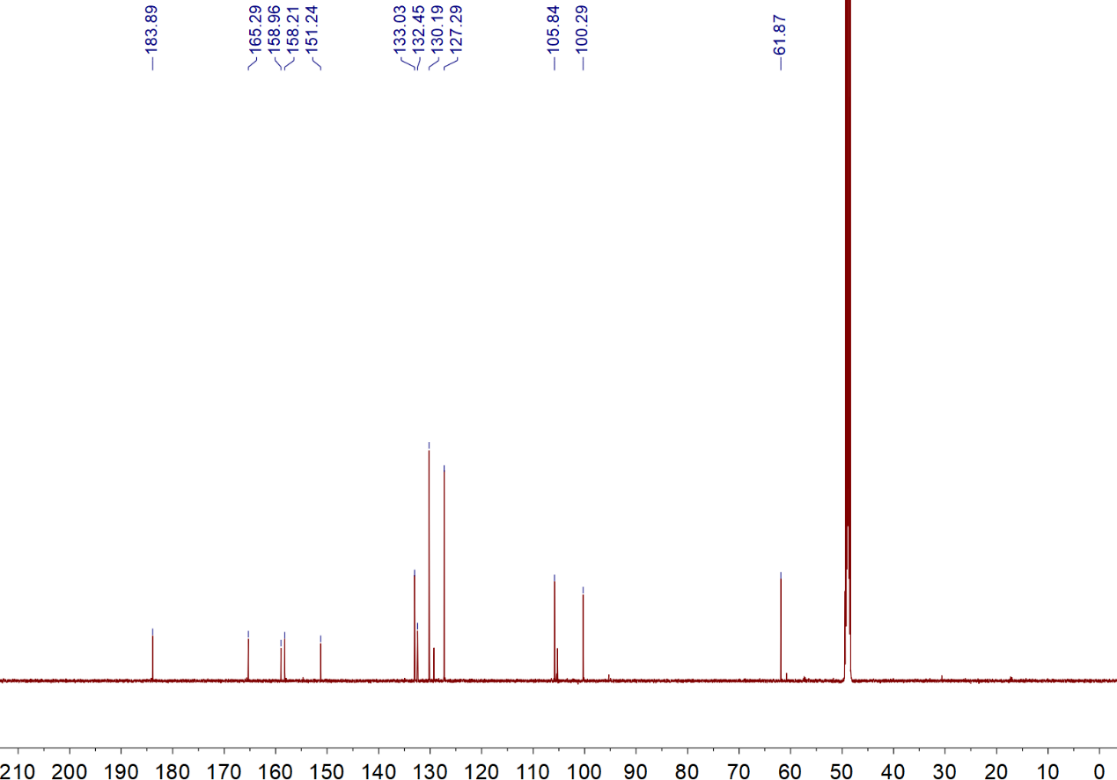


# Figure S31. ^13^C NMR spectrum of compound **6** in Methanol-*d_4_*


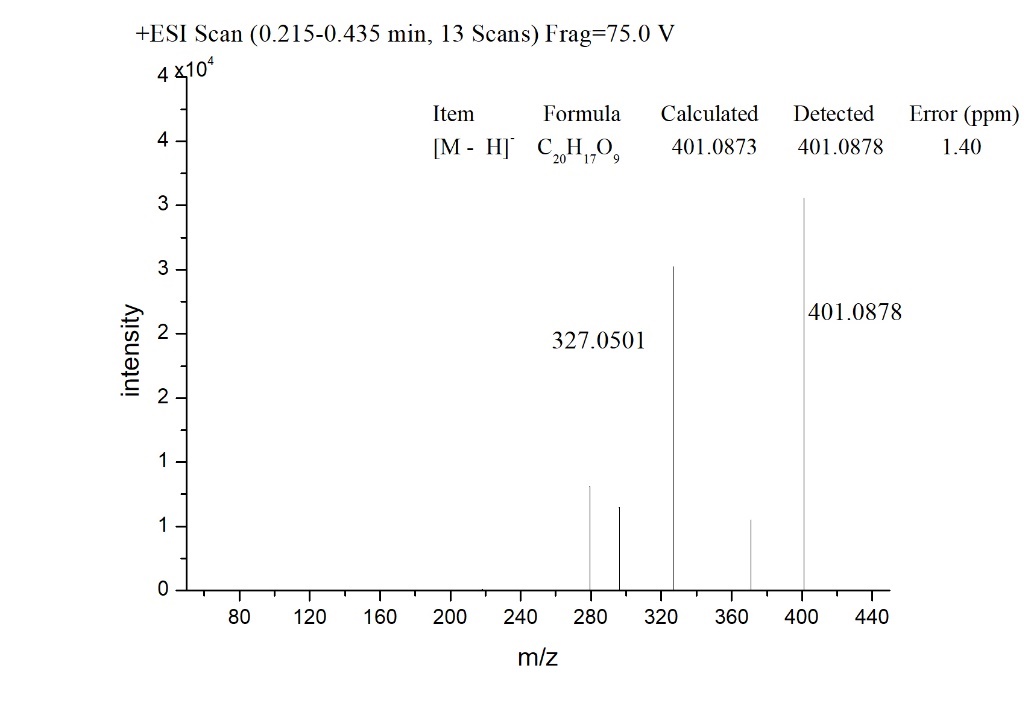


# Figure S32. HR-ESI-MS of compound **44**


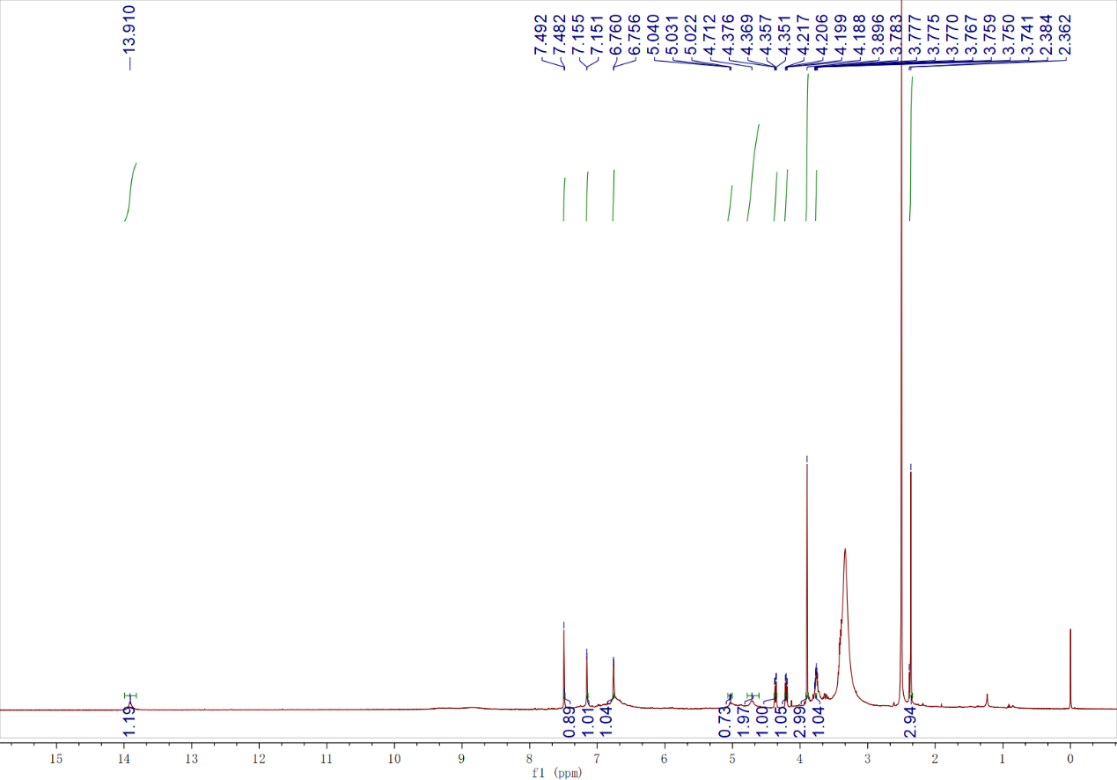


# Figure S33. ^1^H NMR spectrum of compound **44** in DMSO-*d_6_*


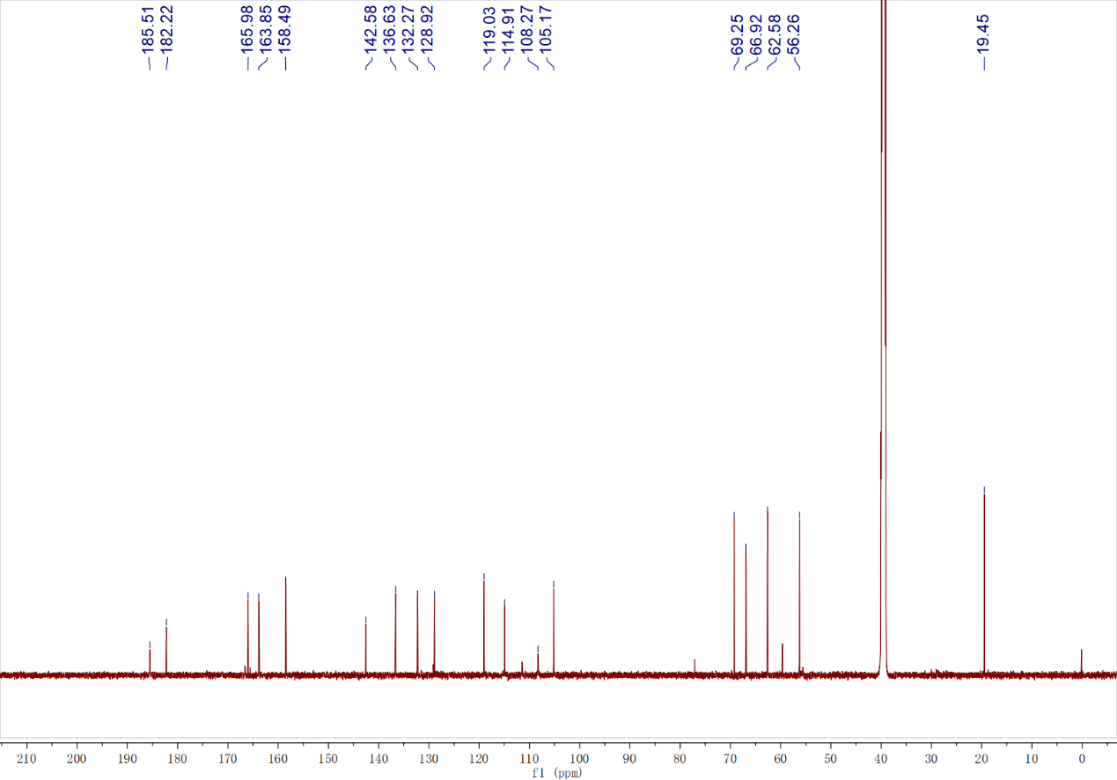


# Figure S34. ^13^C NMR spectrum of compound **44** in DMSO-*d_6_*


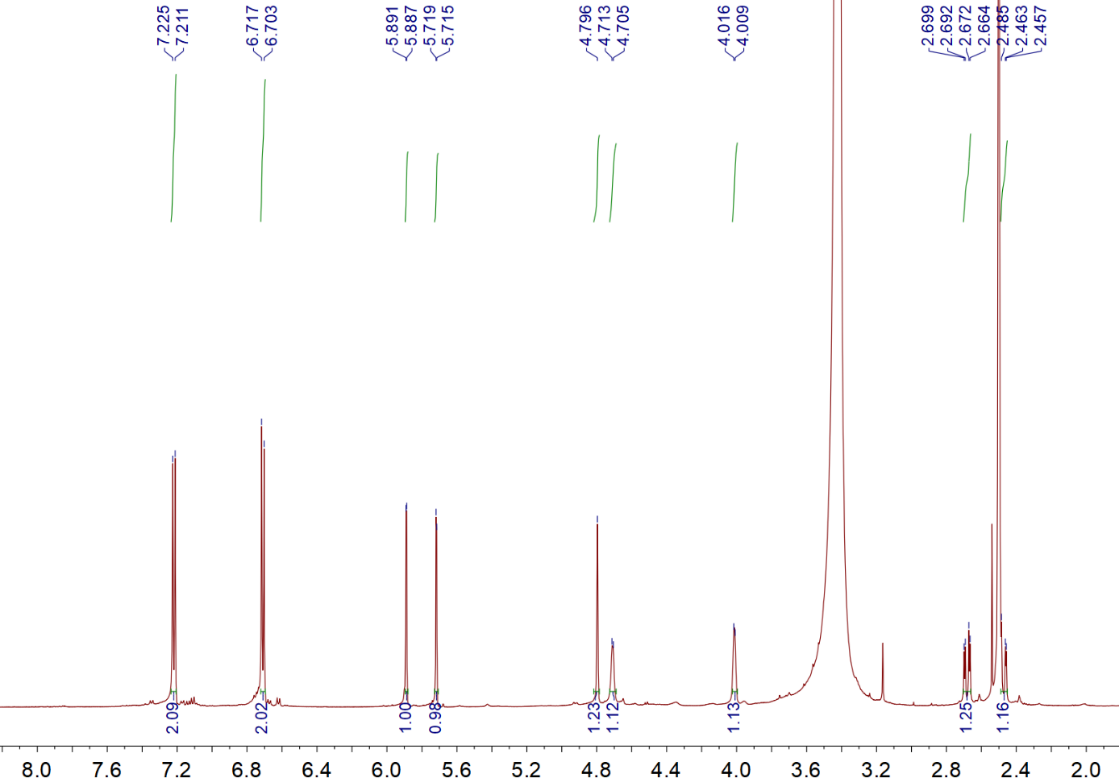


# Figure S35. ^1^H NMR spectrum of compound **2** in DMSO-*d_6_*


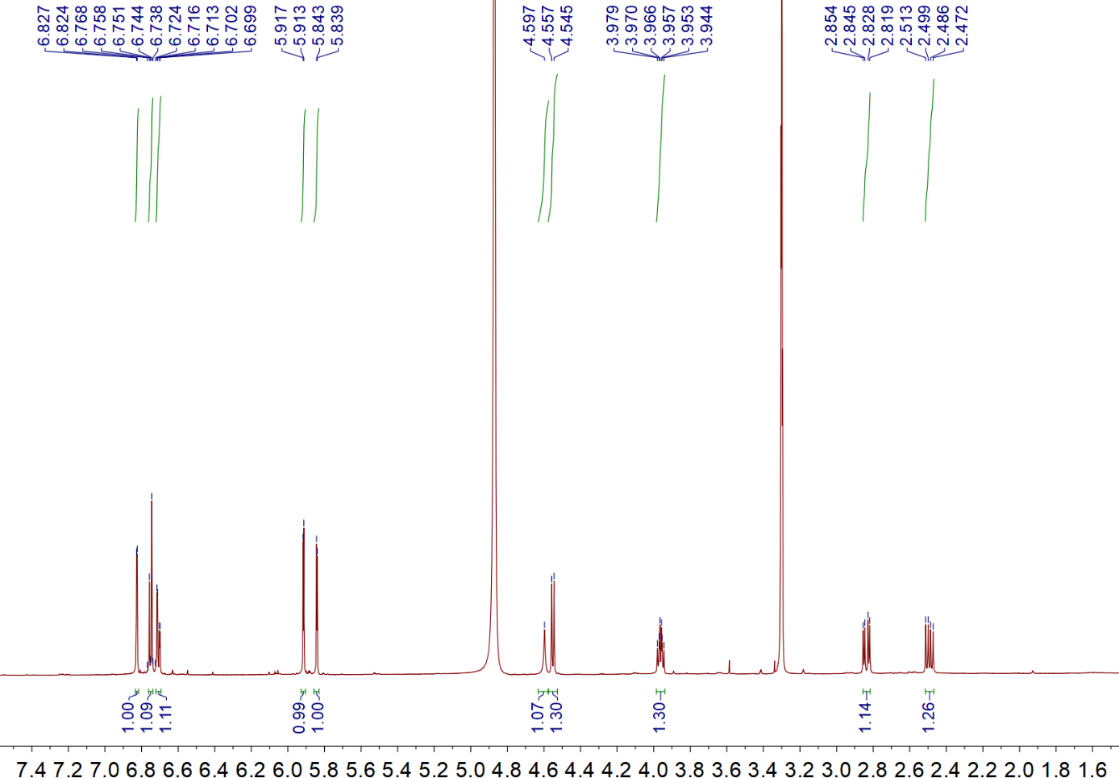


# Figure S36. ^1^H NMR spectrum of compound **3** in Methanol-*d*_4_


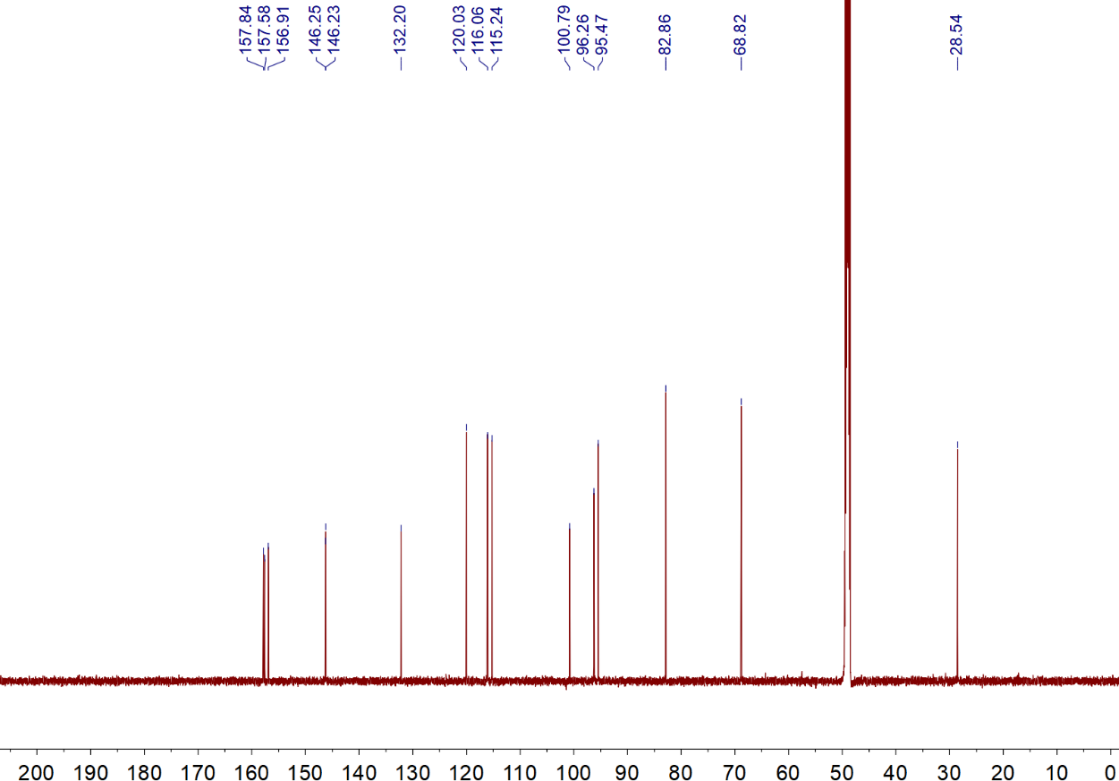


# Figure S37. ^13^C NMR spectrum of compound **3** in Methanol-*d*_4_


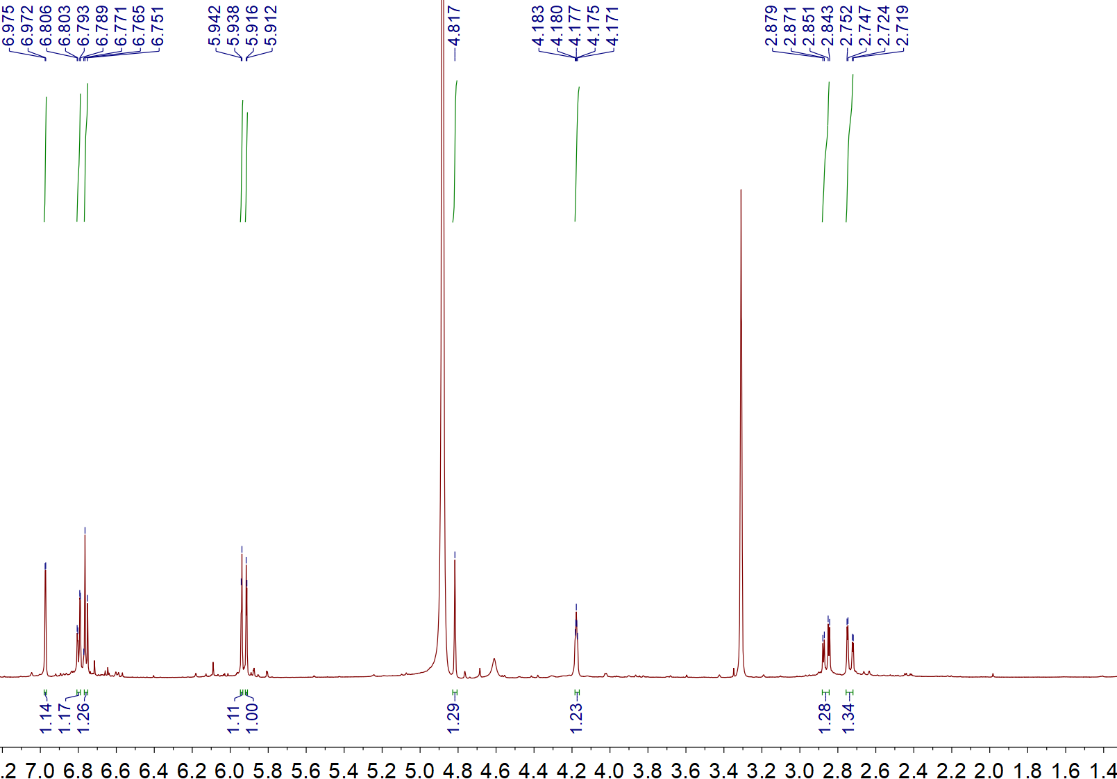


# Figure S38. 1H NMR spectrum of compound 3 in Methanol-*d*_4_


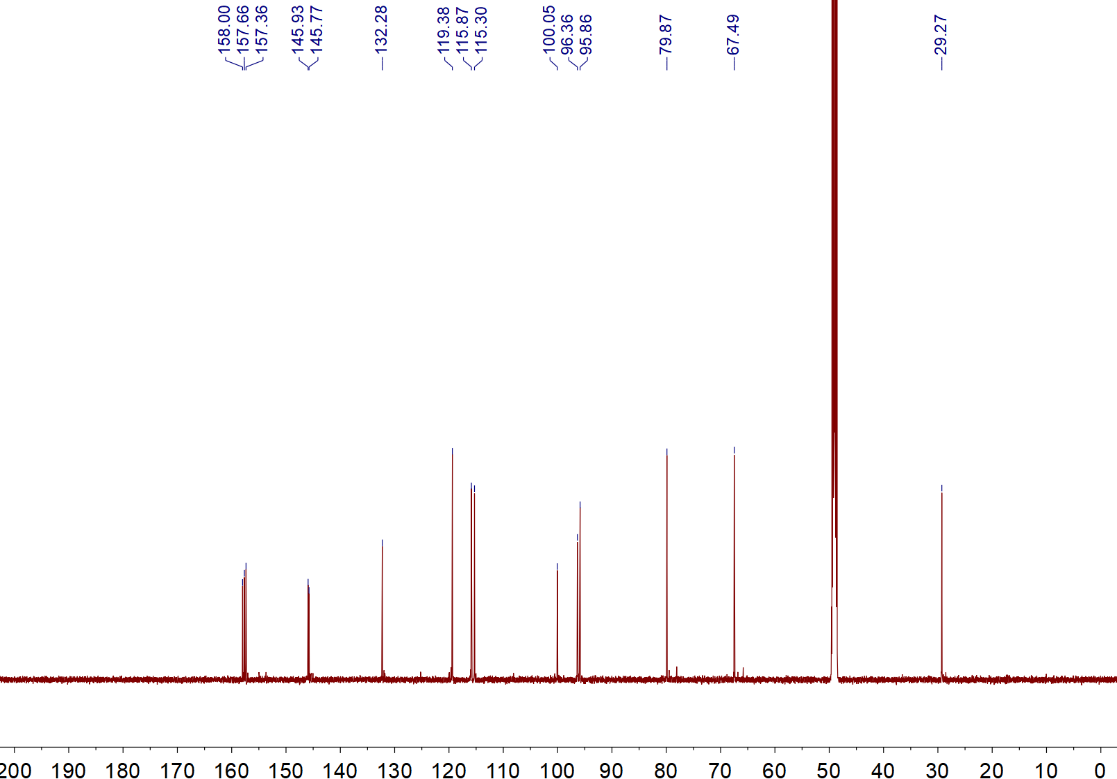


# Figure S39. ^13^C NMR spectrum of compound **4** in Methanol-*d*_4_


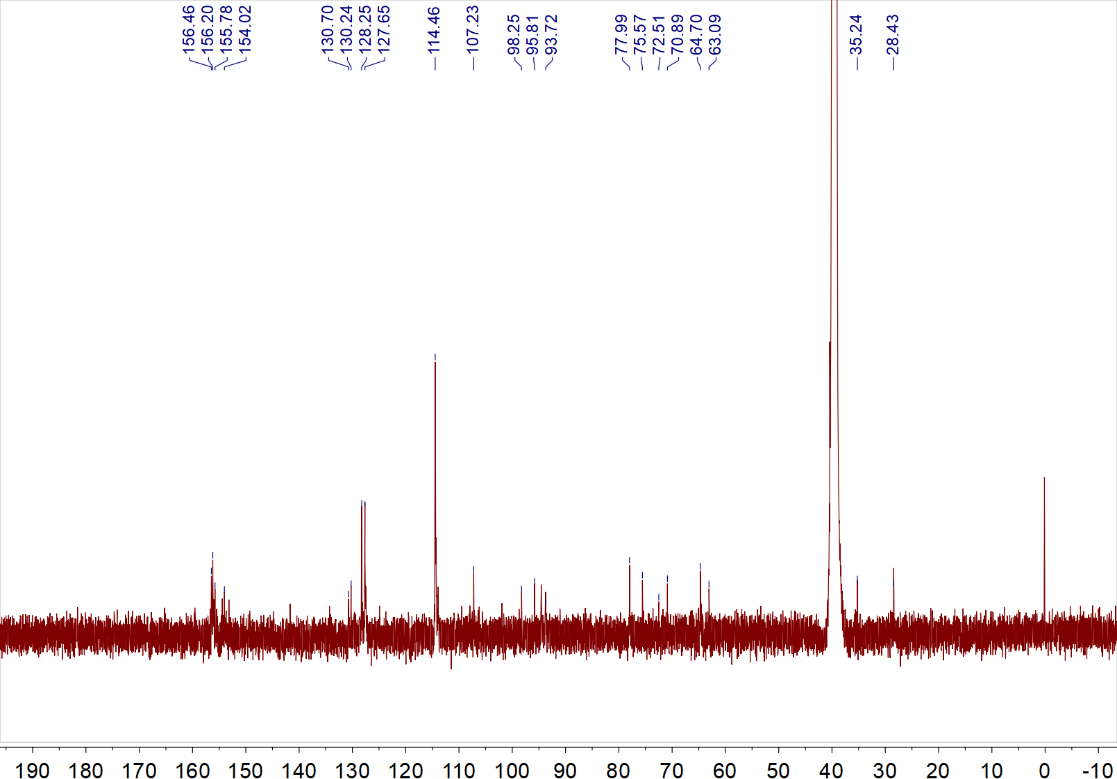


# Figure S40. ^13^C NMR spectrum of compound **5** in DMSO-*d_6_*


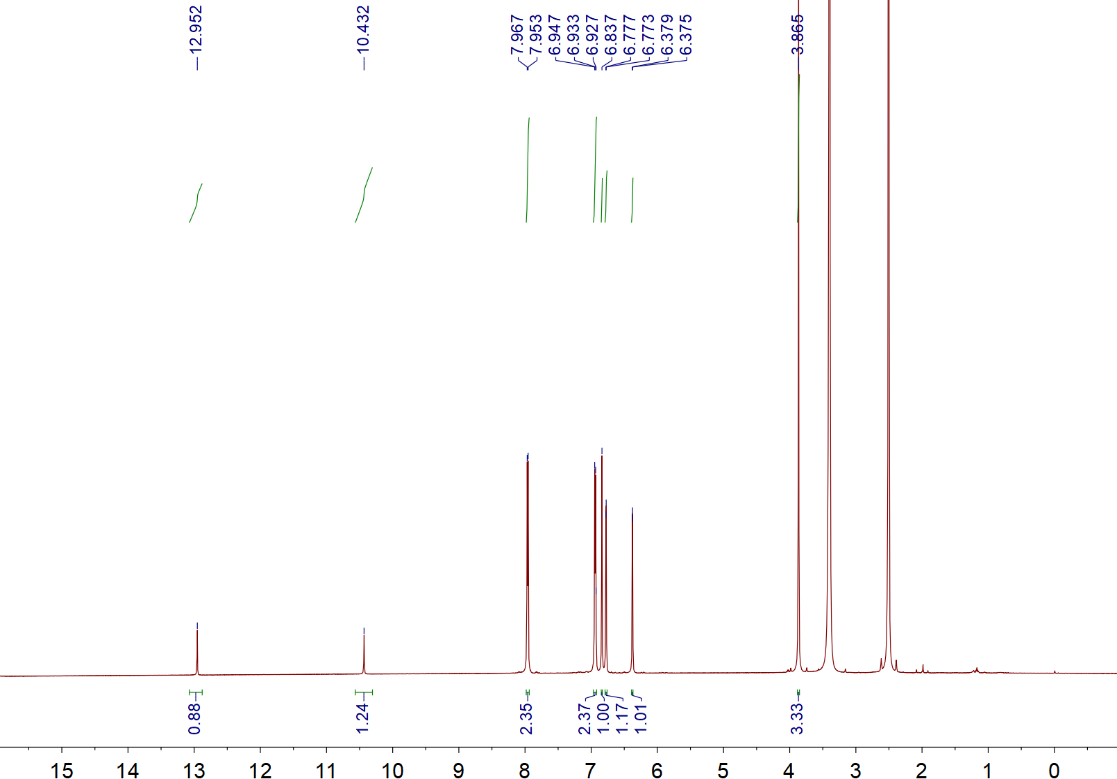


# Figure S41. ^1^H NMR spectrum of compound **7** in DMSO-*d*_6_


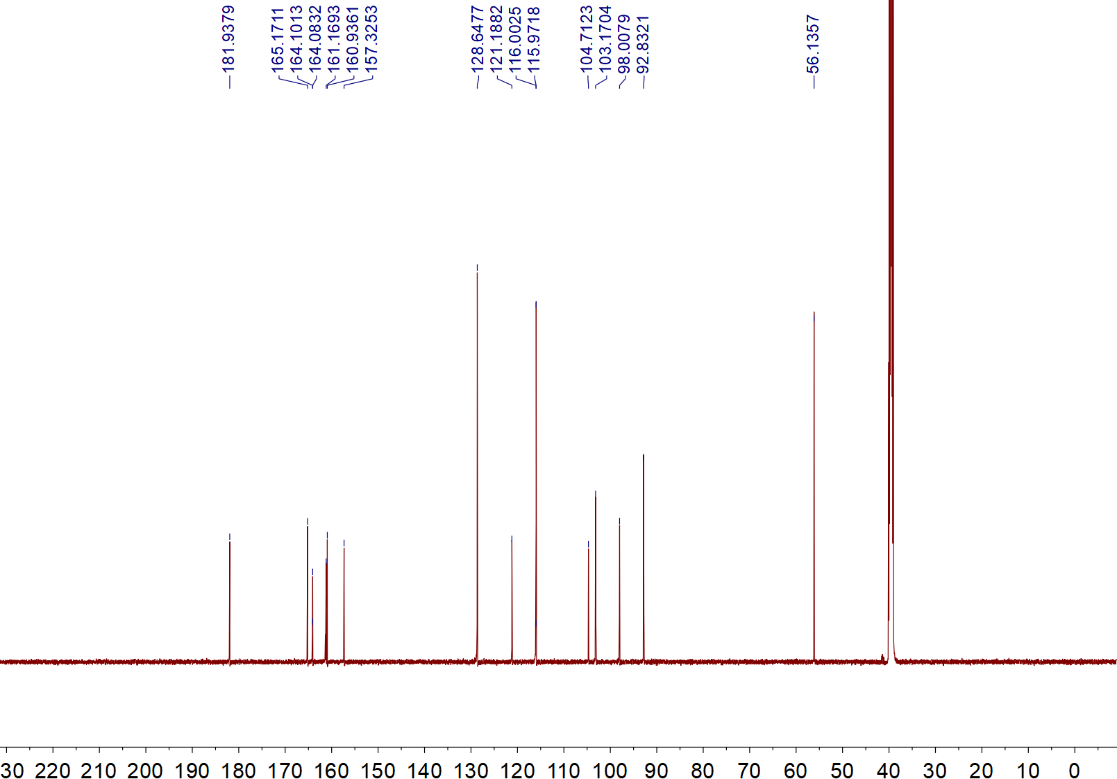


# Figure S42. ^13^C NMR spectrum of compound **7** in DMSO-*d*_6_


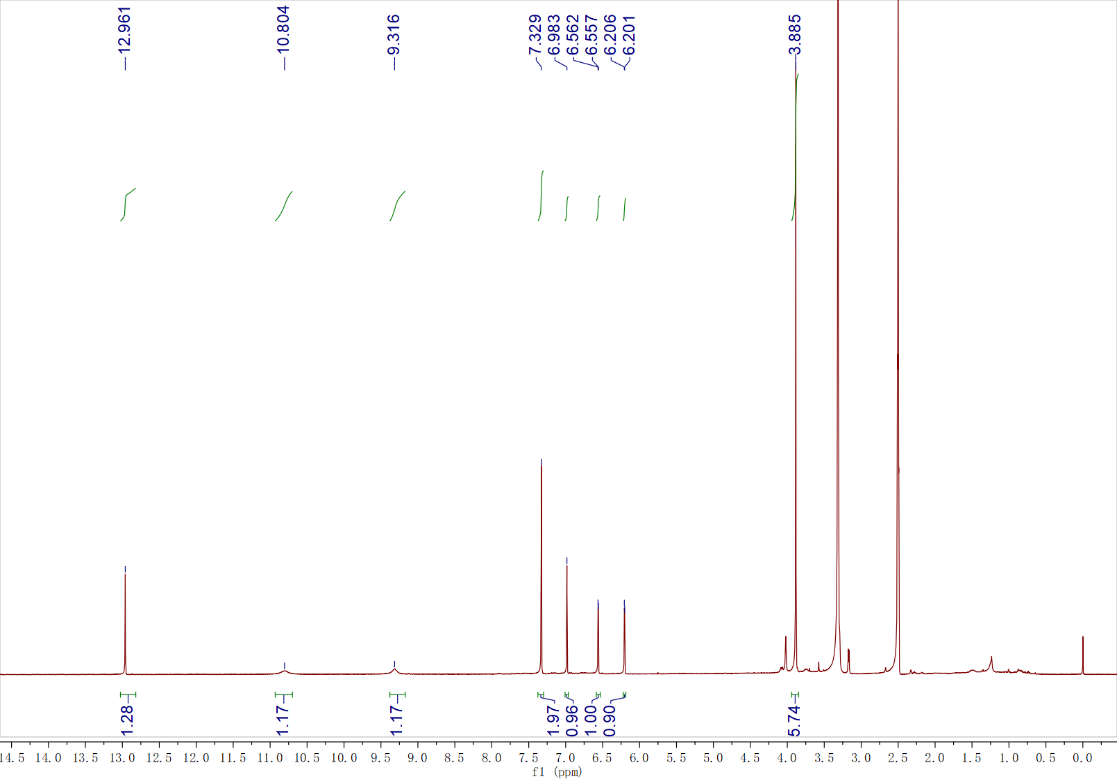


# Figure S43. ^1^H NMR spectrum of compound **8** in DMSO-*d*_6_


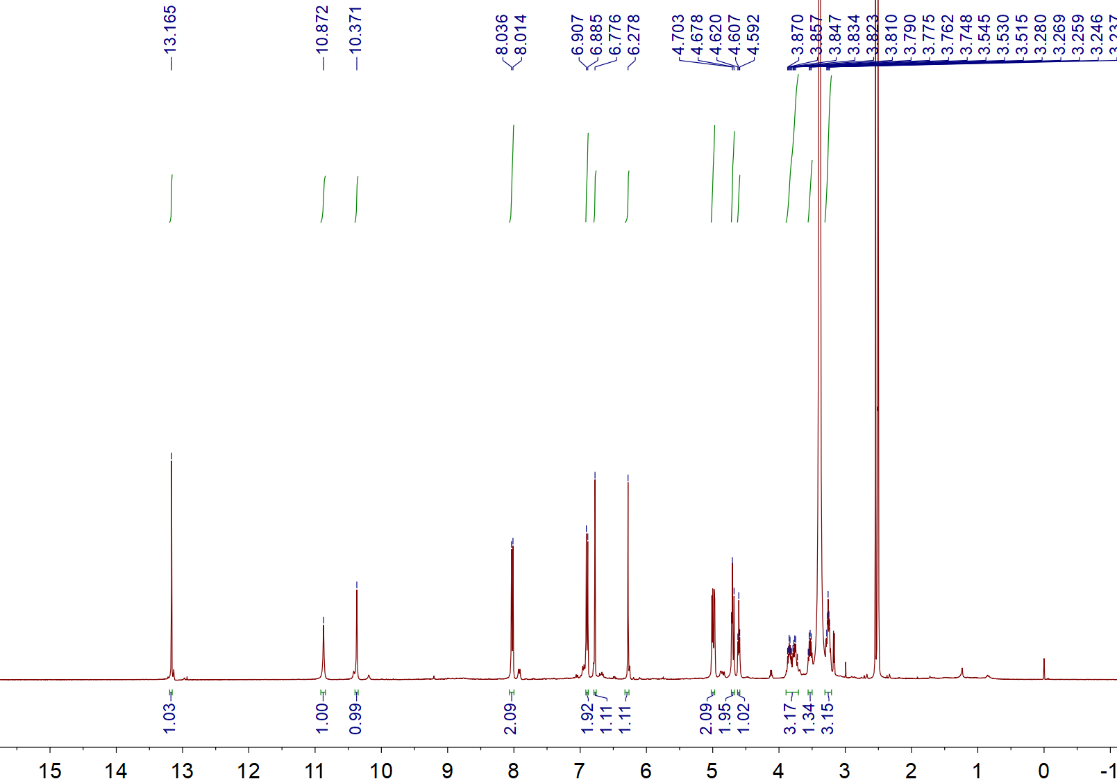


# Figure S44. ^1^H NMR spectrum of compound **9** in DMSO-*d*_6_


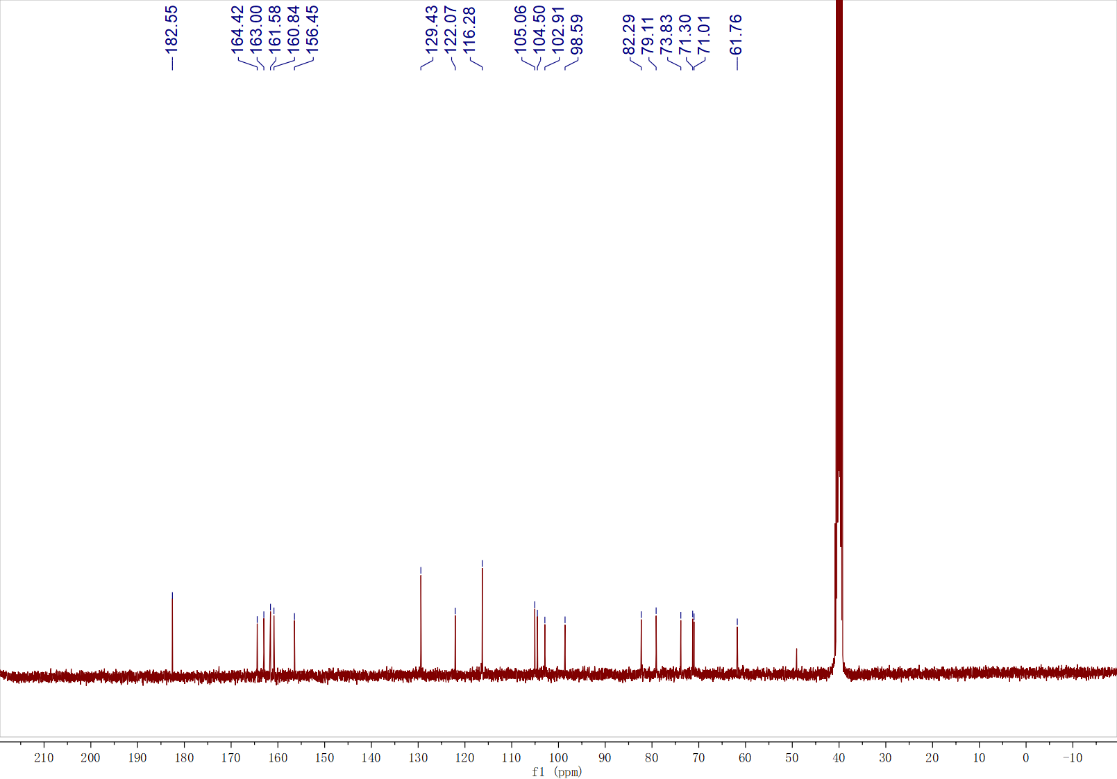


# Figure S45. ^13^C NMR spectrum of compound **9** in DMSO-*d*_6_


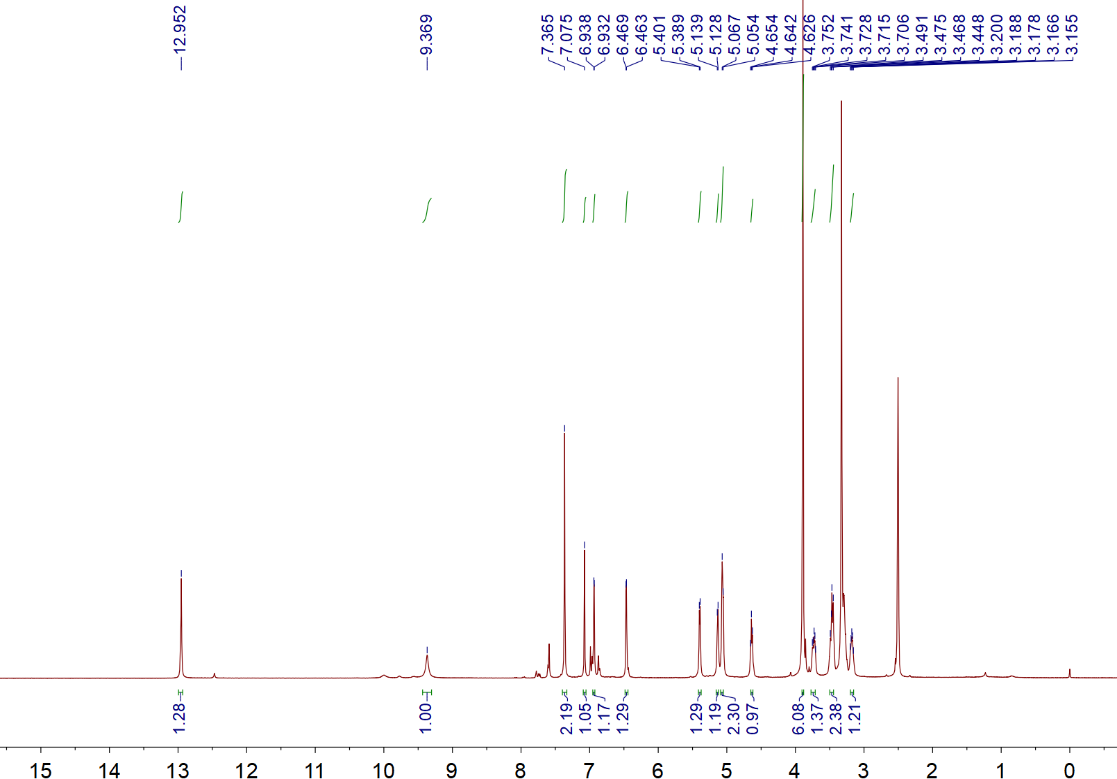


# Figure S46. ^1^H NMR spectrum of compound **10** in DMSO-*d*_6_


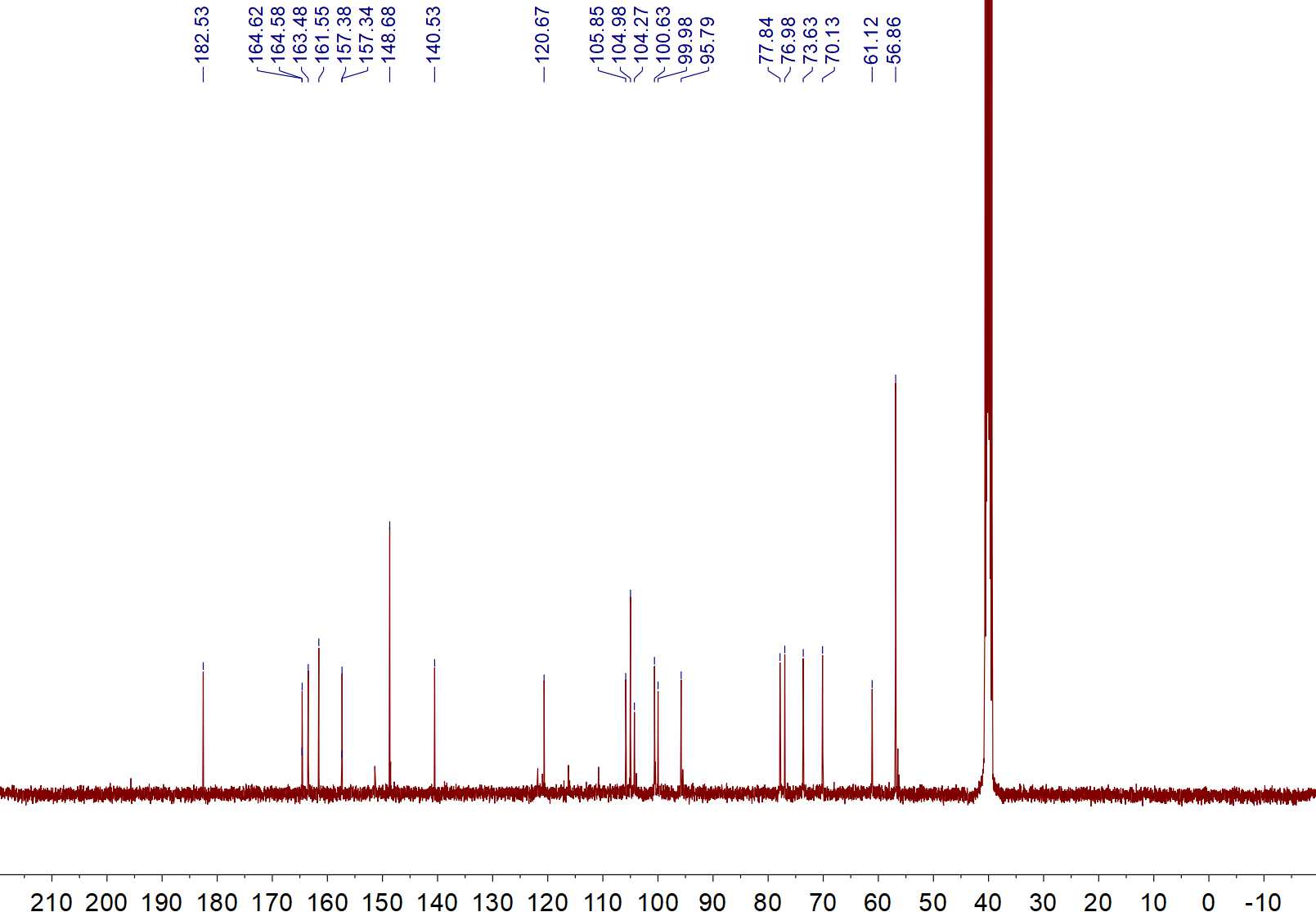


# Figure S47. ^13^C NMR spectrum of compound **10** in DMSO-*d*_6_


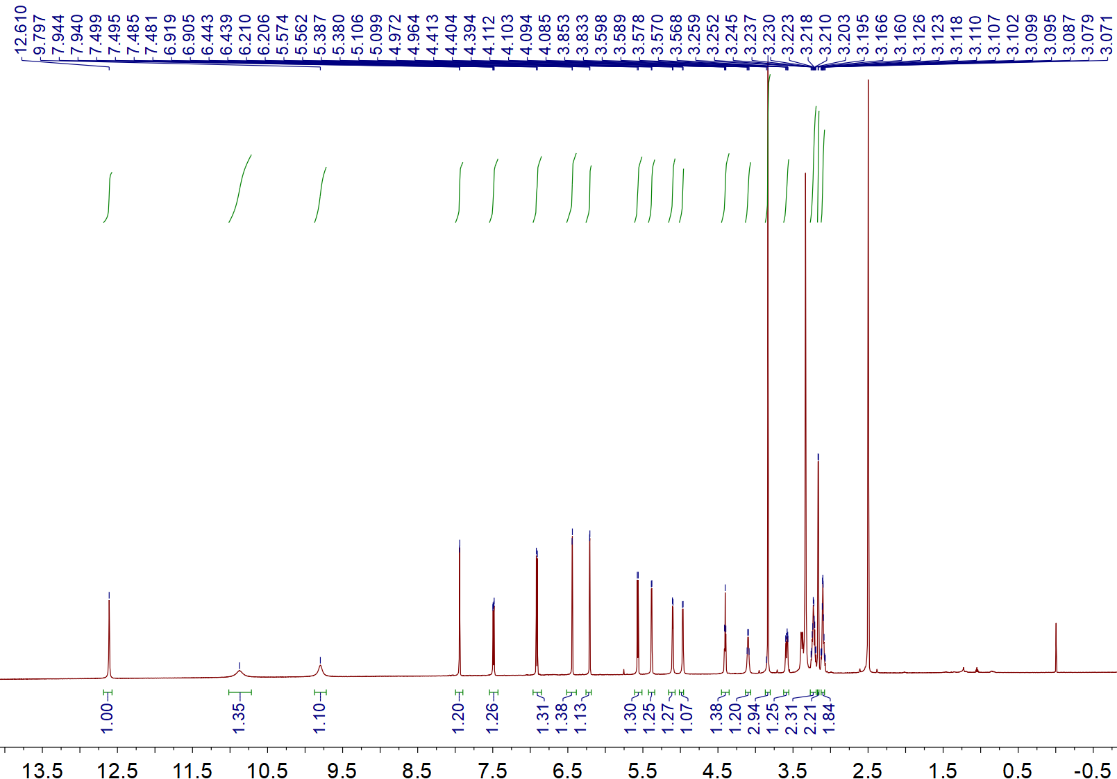


# Figure S48. ^1^H NMR spectrum of compound **11** in DMSO-*d*_6_


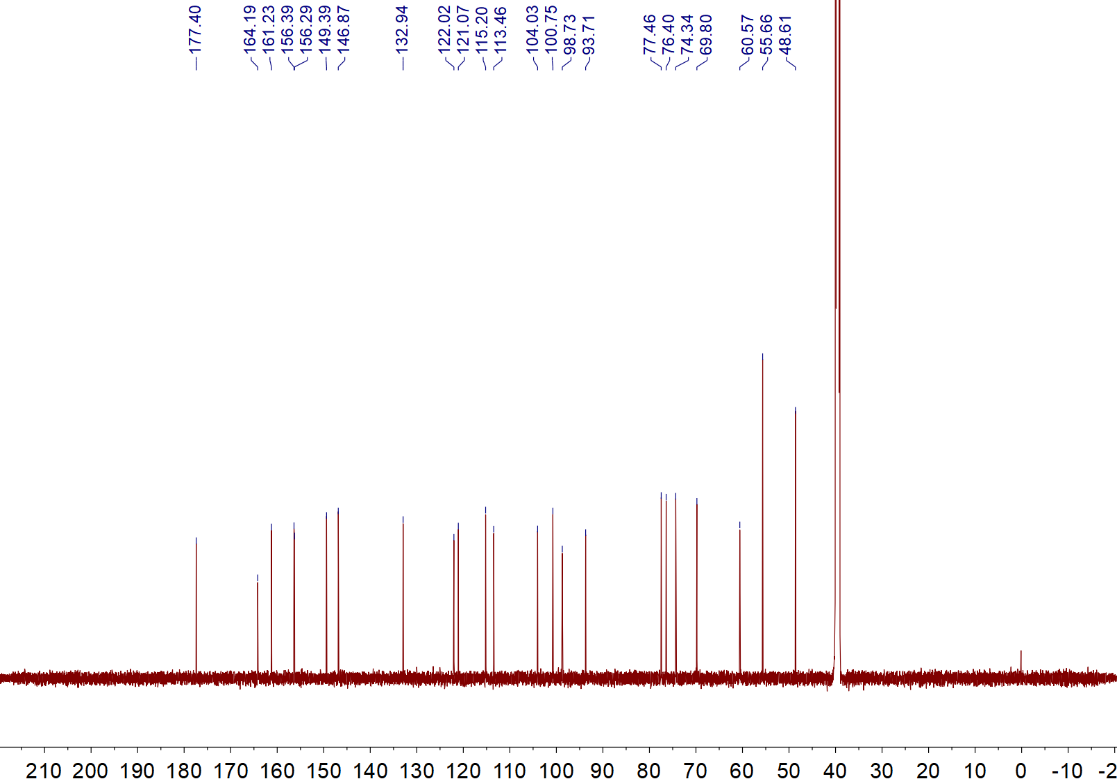


# Figure S49. ^13^C NMR spectrum of compound **11** in DMSO-*d*_6_


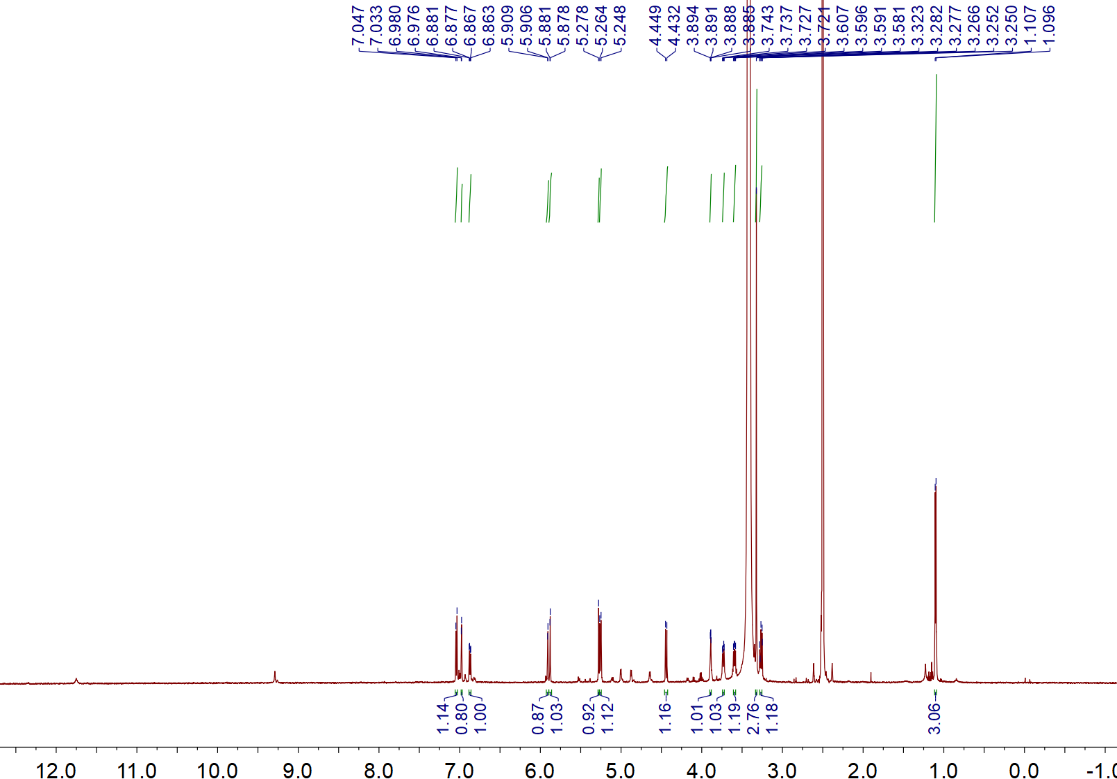


# Figure S50. ^1^H NMR spectrum of compound **12** in DMSO-*d*_6_


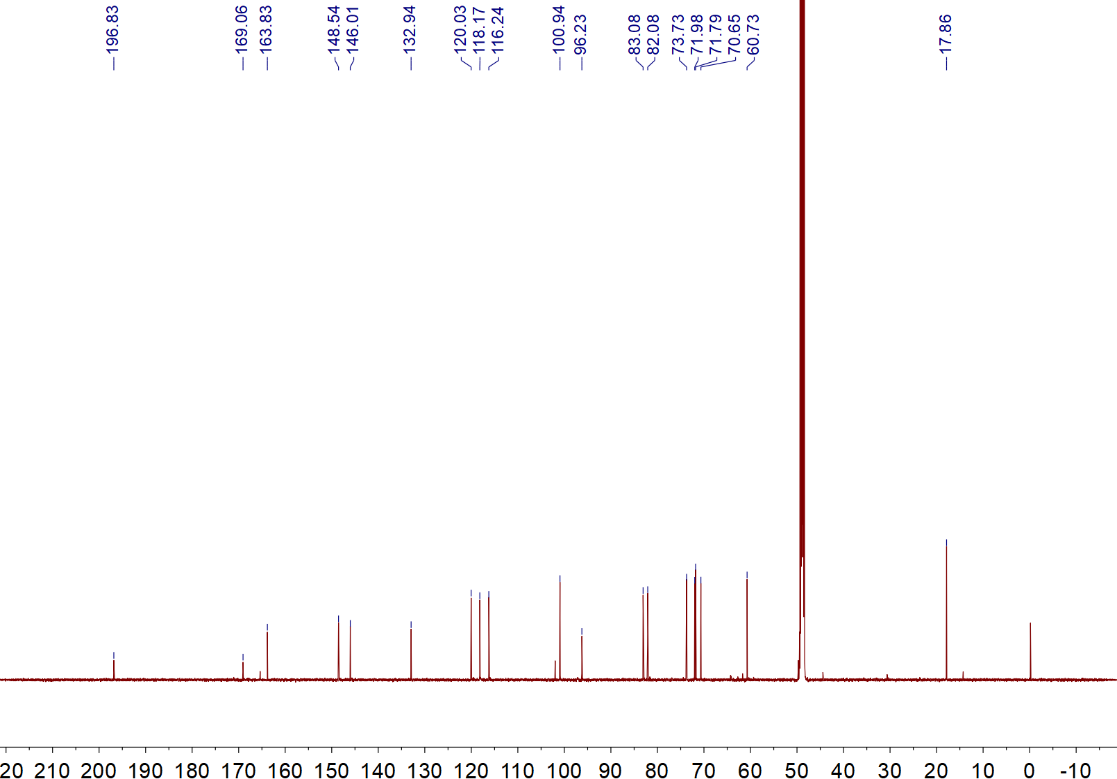


# Figure S51. ^13^C NMR spectrum of compound **12** in DMSO-*d*_6_


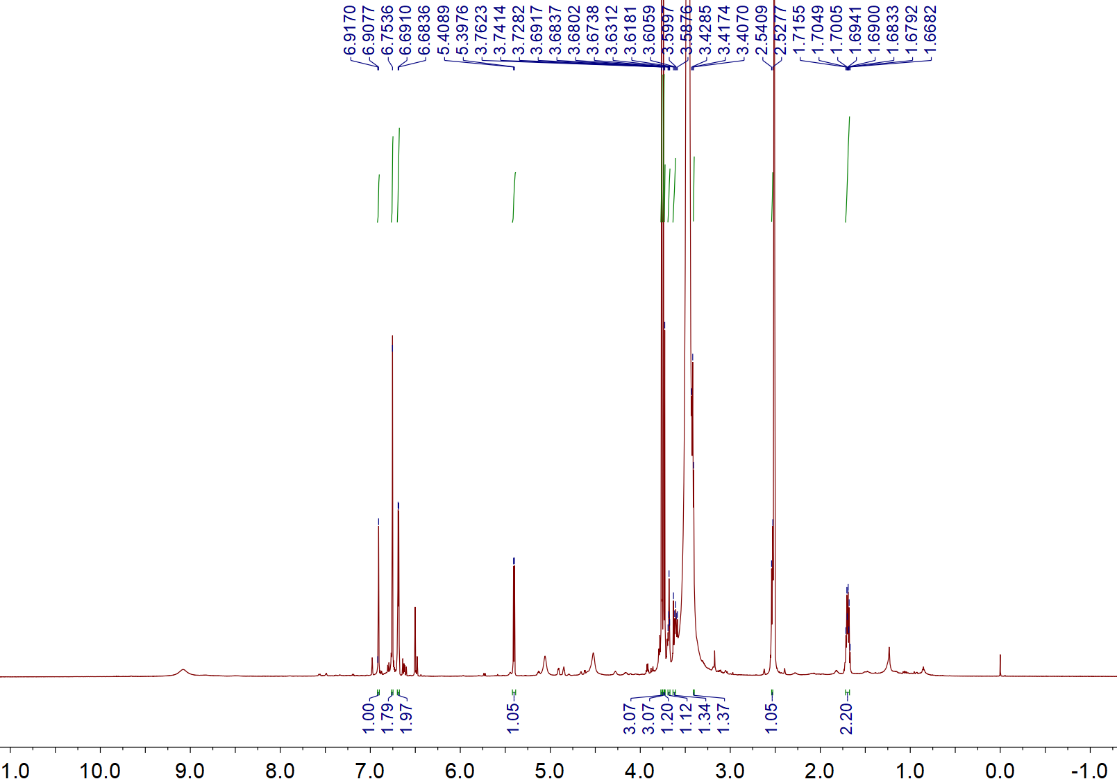


# Figure S52. ^1^H NMR spectrum of compound **13** in DMSO-*d*_6_


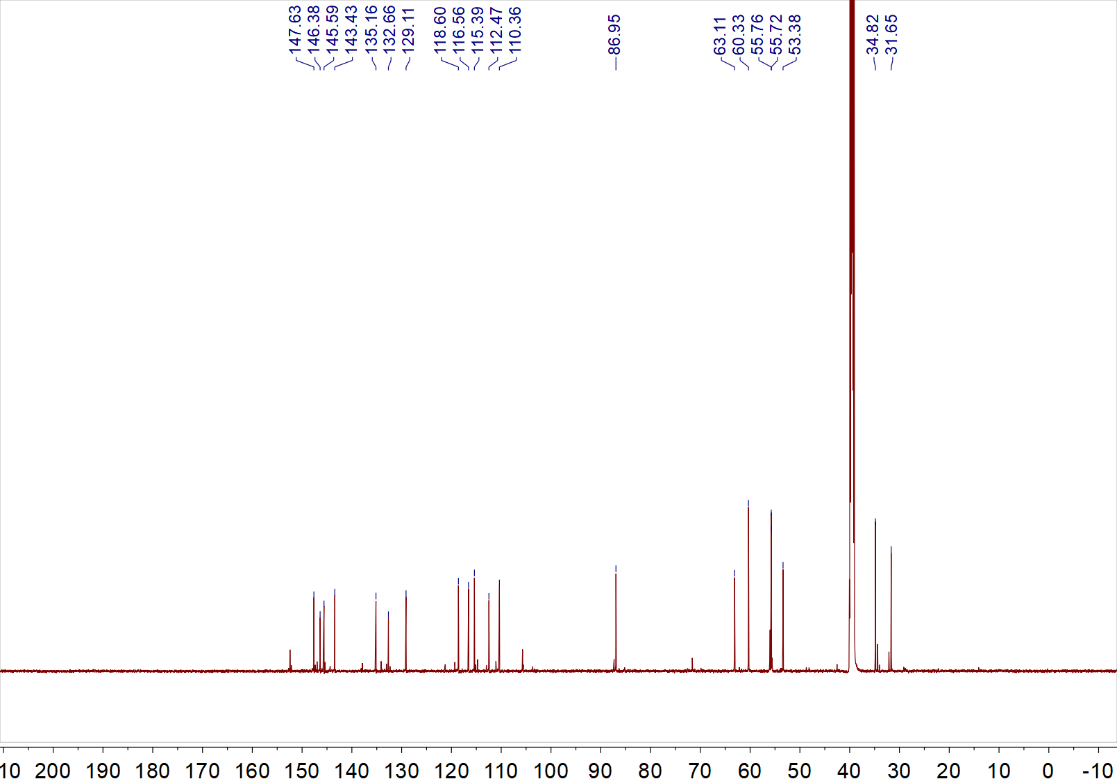


# Figure S53. ^13^C NMR spectrum of compound **13** in DMSO-*d*_6_


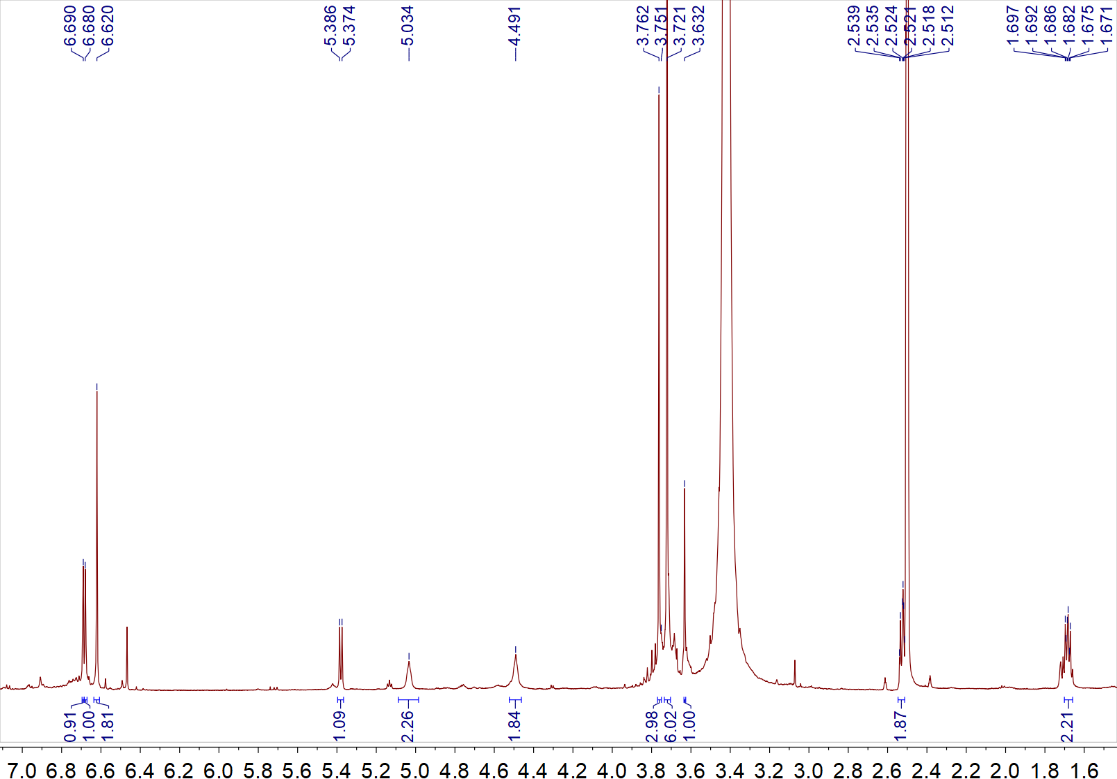


# Figure S54. ^1^H NMR spectrum of compound **14** in DMSO-*d*_6_


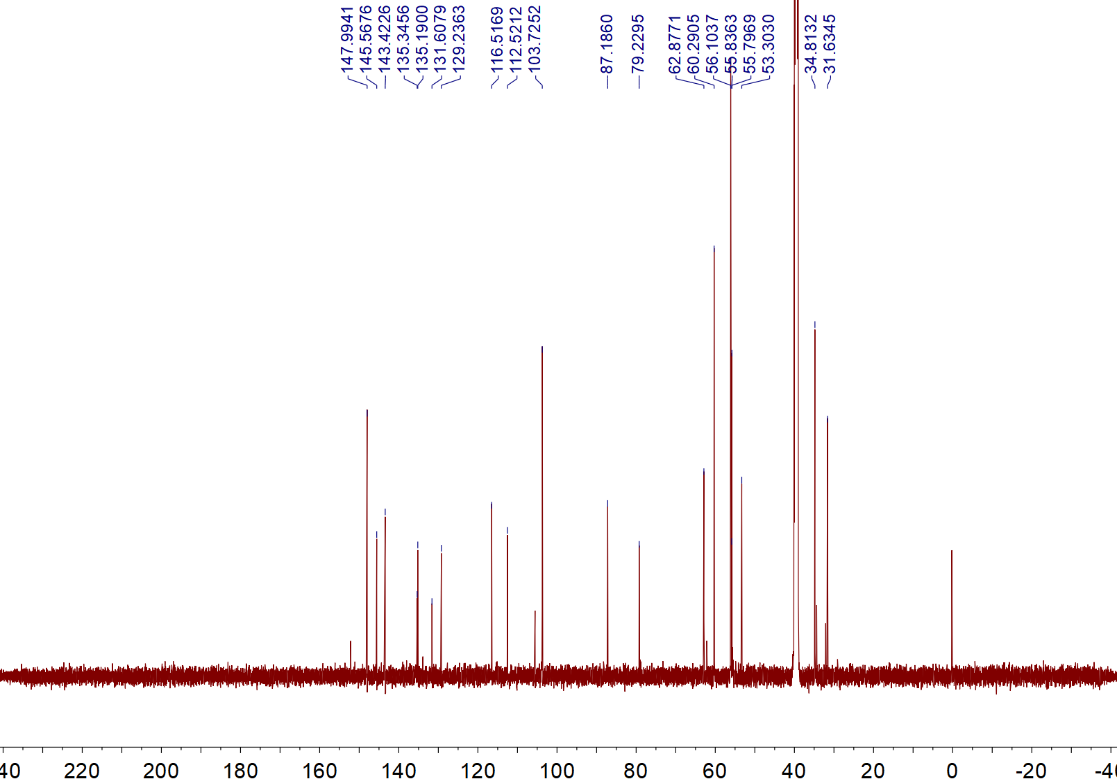


# Figure S55. ^13^C NMR spectrum of compound **14** in DMSO-*d*_6_


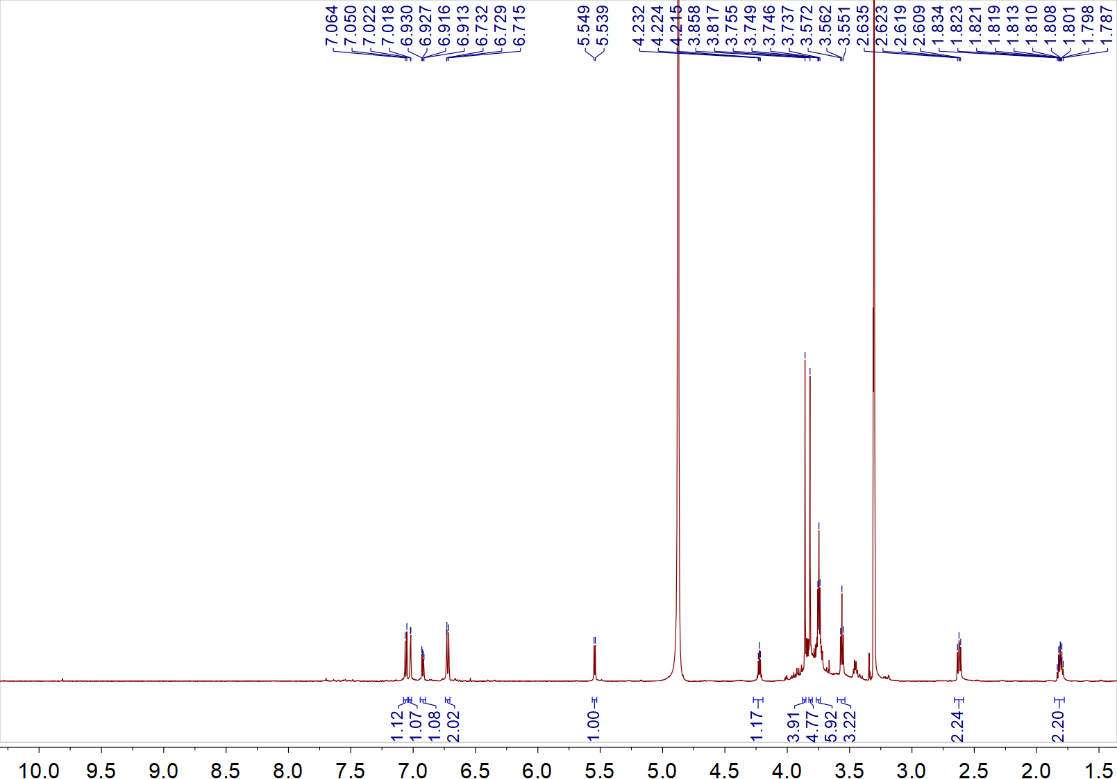


# Figure S56. ^1^H NMR spectrum of compound **15** in DMSO-*d*_6_


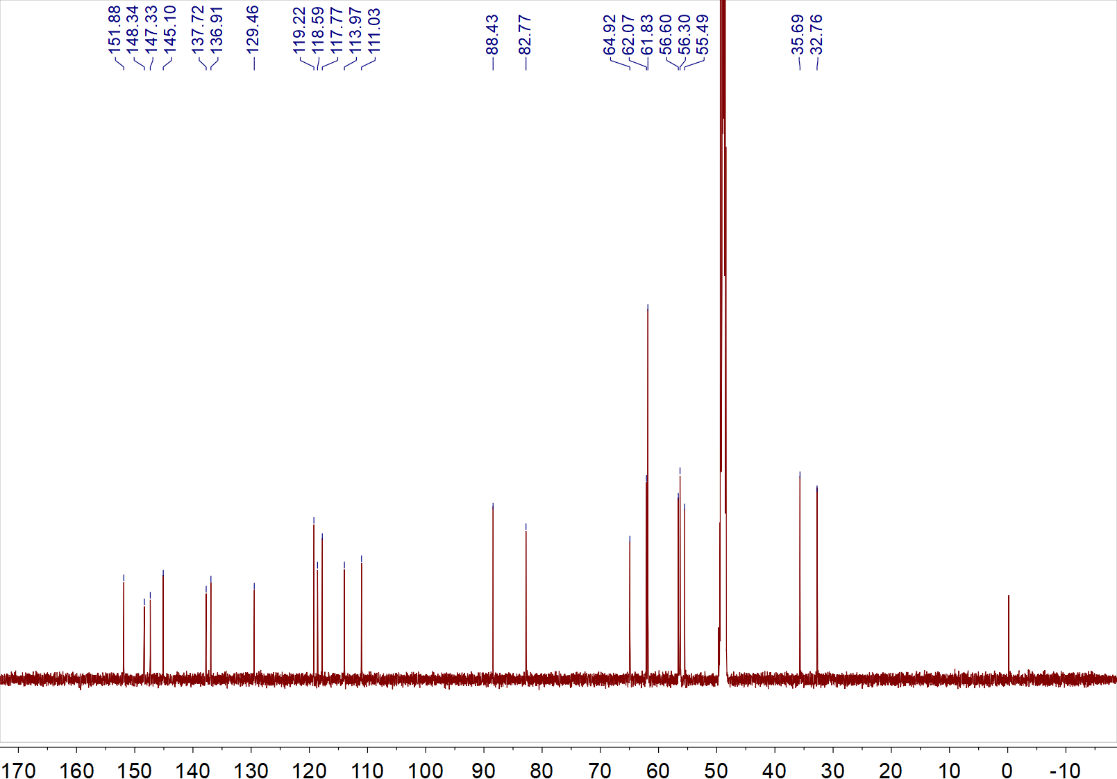


# Figure S57. ^13^C NMR spectrum of compound **15** in DMSO-*d*_6_


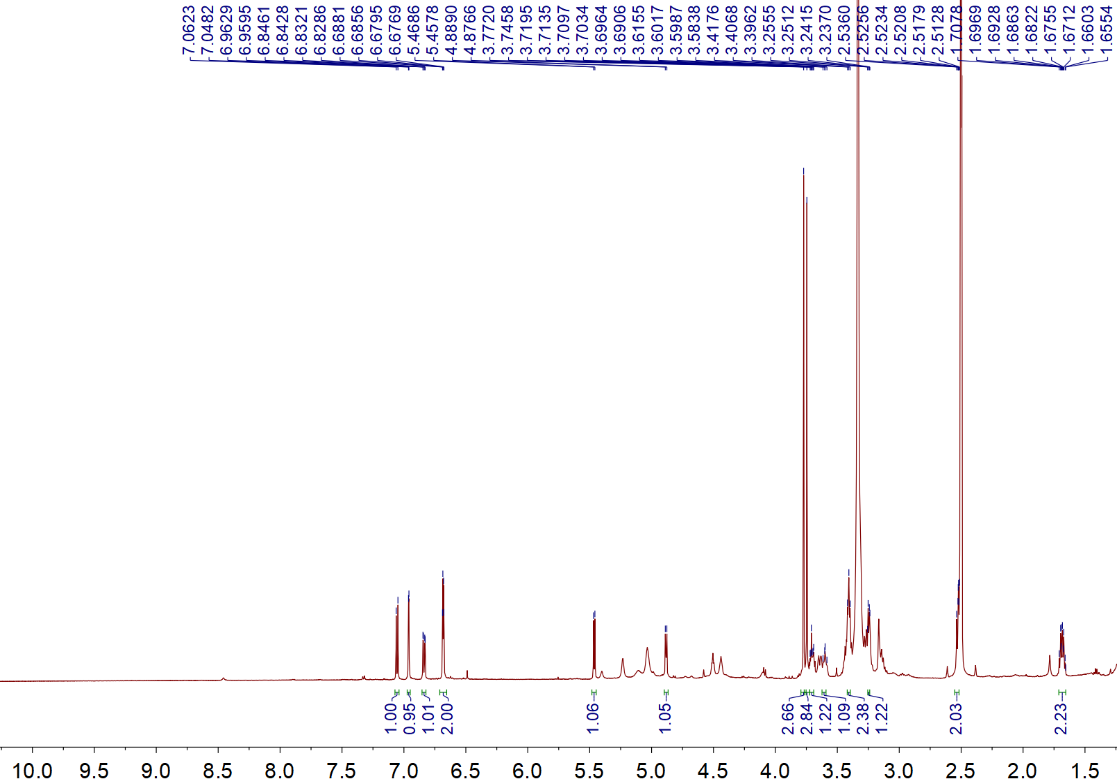


# Figure S58. ^1^H NMR spectrum of compound **16** in DMSO-*d*_6_


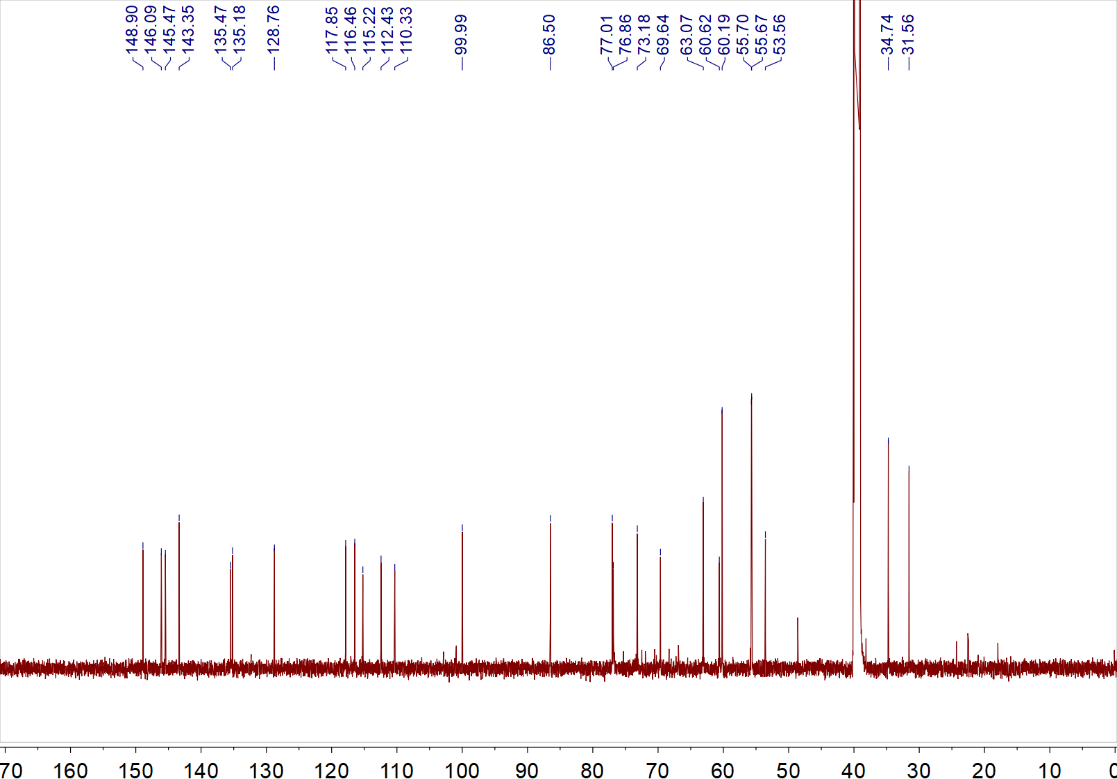


# Figure S59. ^13^C NMR spectrum of compound **16** in DMSO-*d*_6_


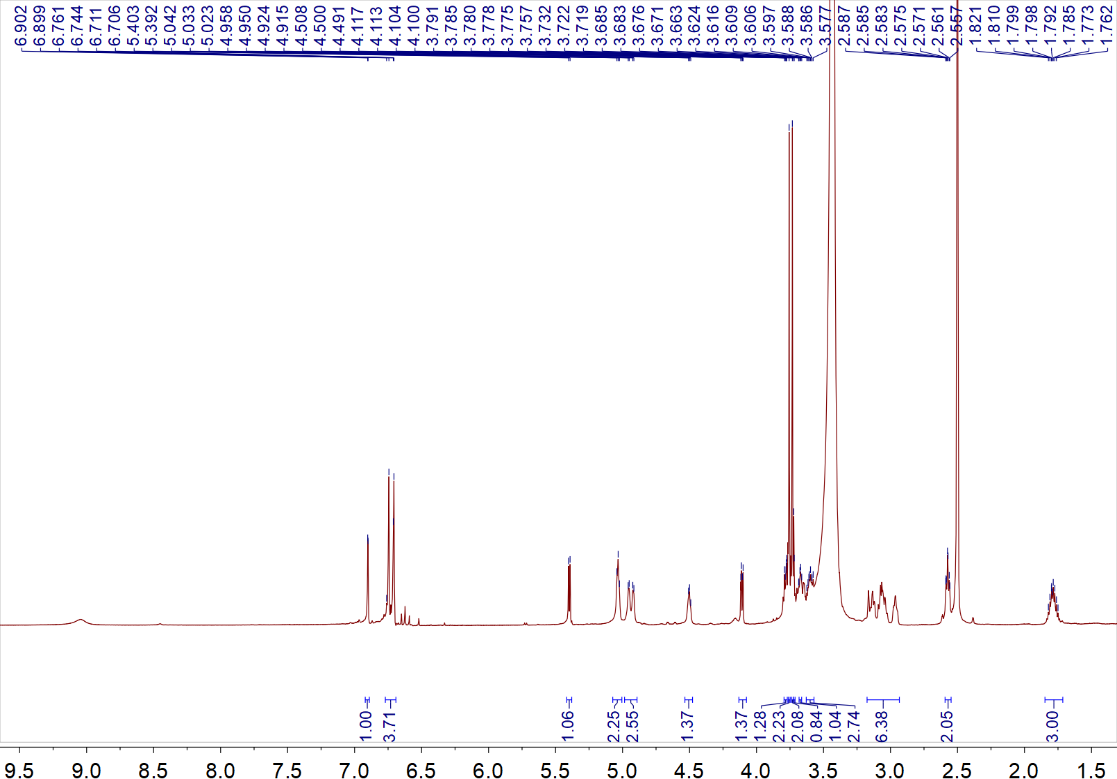


# Figure S60. ^1^H NMR spectrum of compound **17** in DMSO-*d*_6_


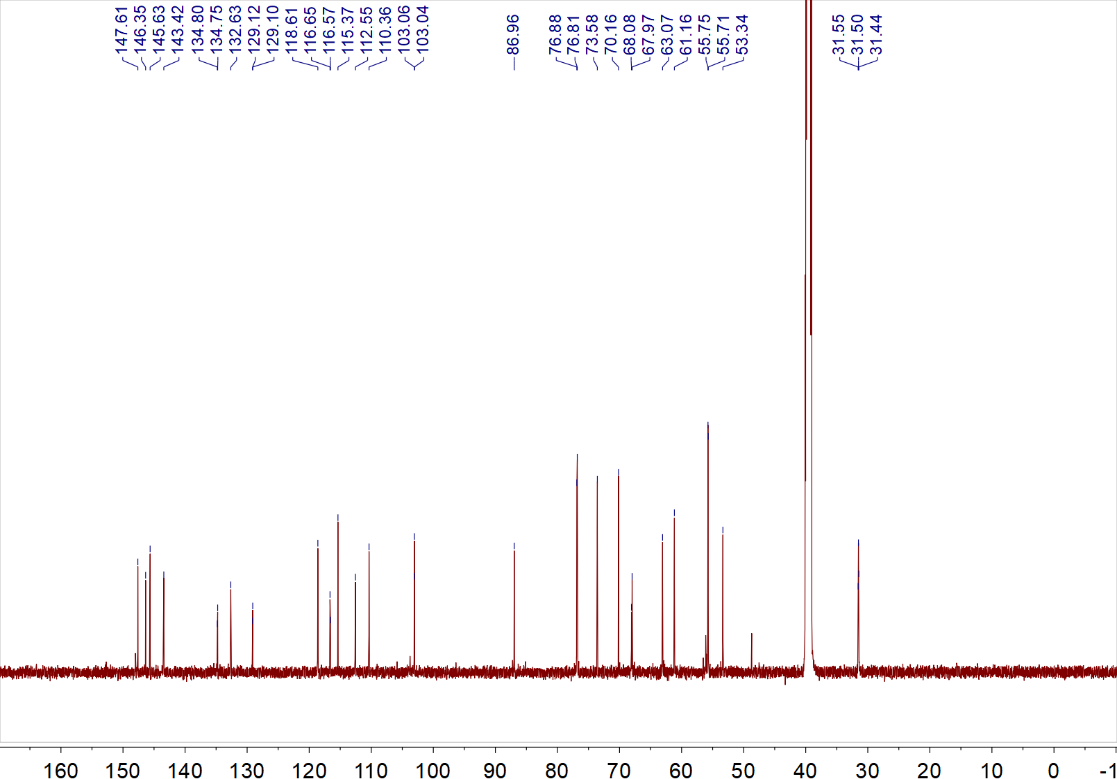


# Figure S61. ^13^C NMR spectrum of compound **17** in DMSO-*d*_6_


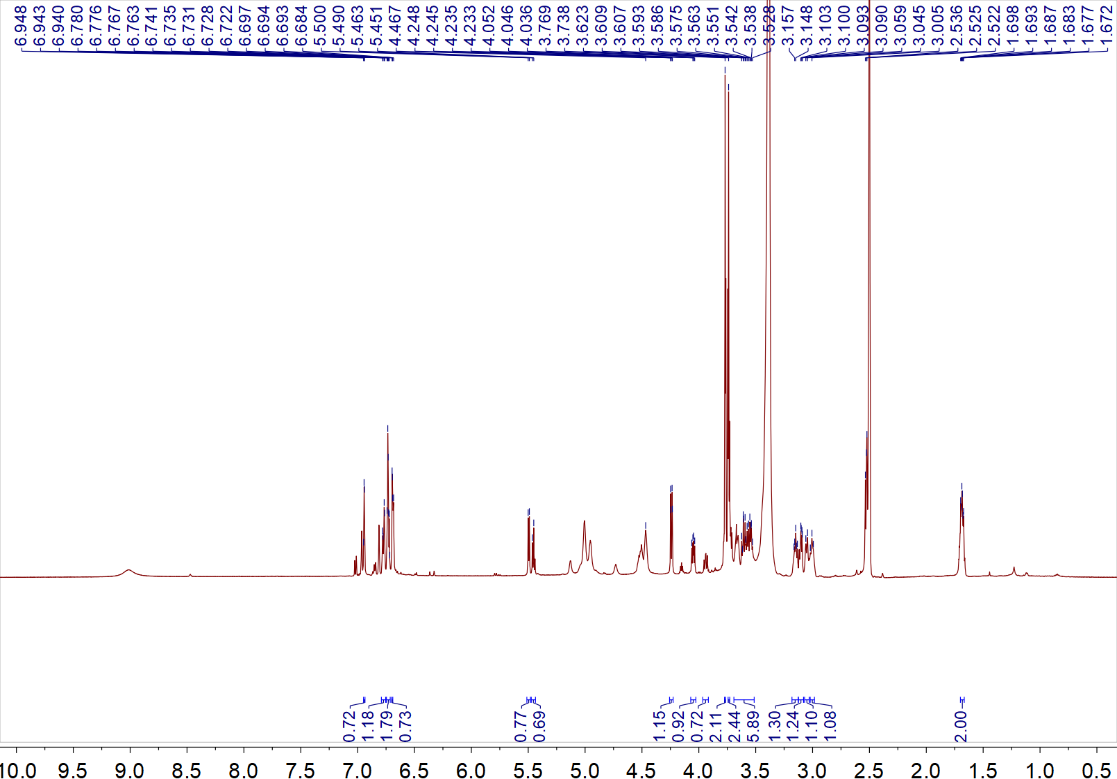


# Figure S62. ^1^H NMR spectrum of compound **18** in DMSO-*d*_6_


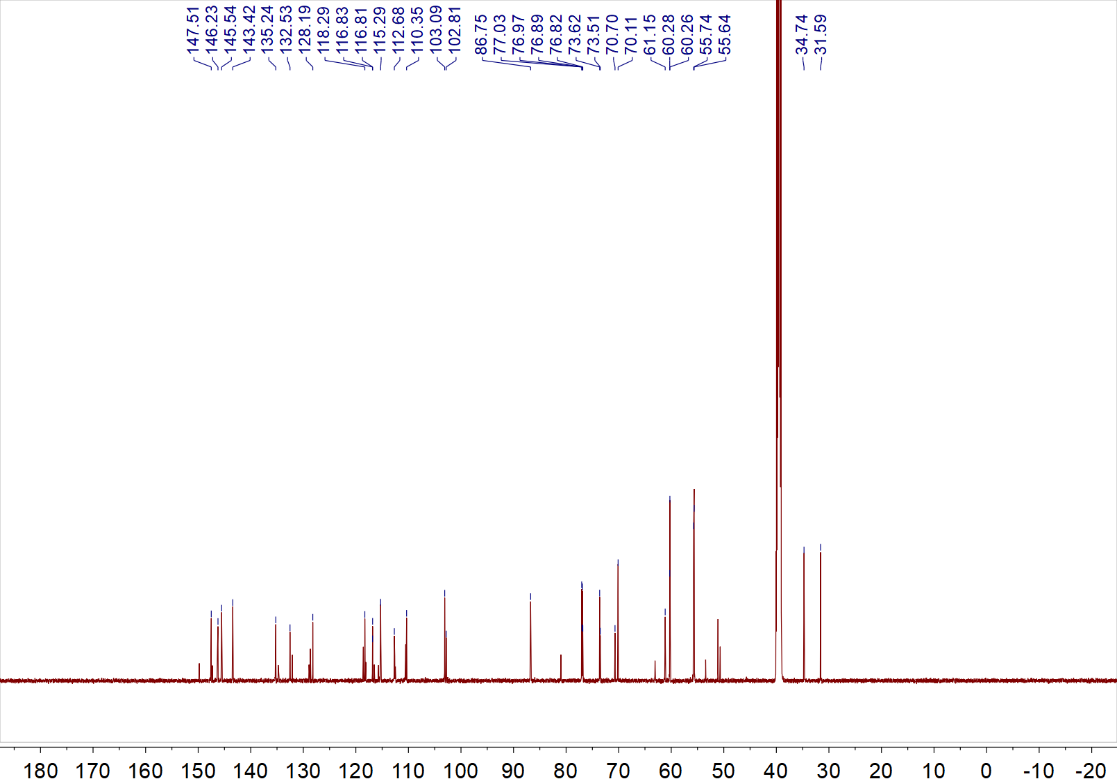


# Figure S63. ^13^C NMR spectrum of compound **18** in DMSO-*d*_6_


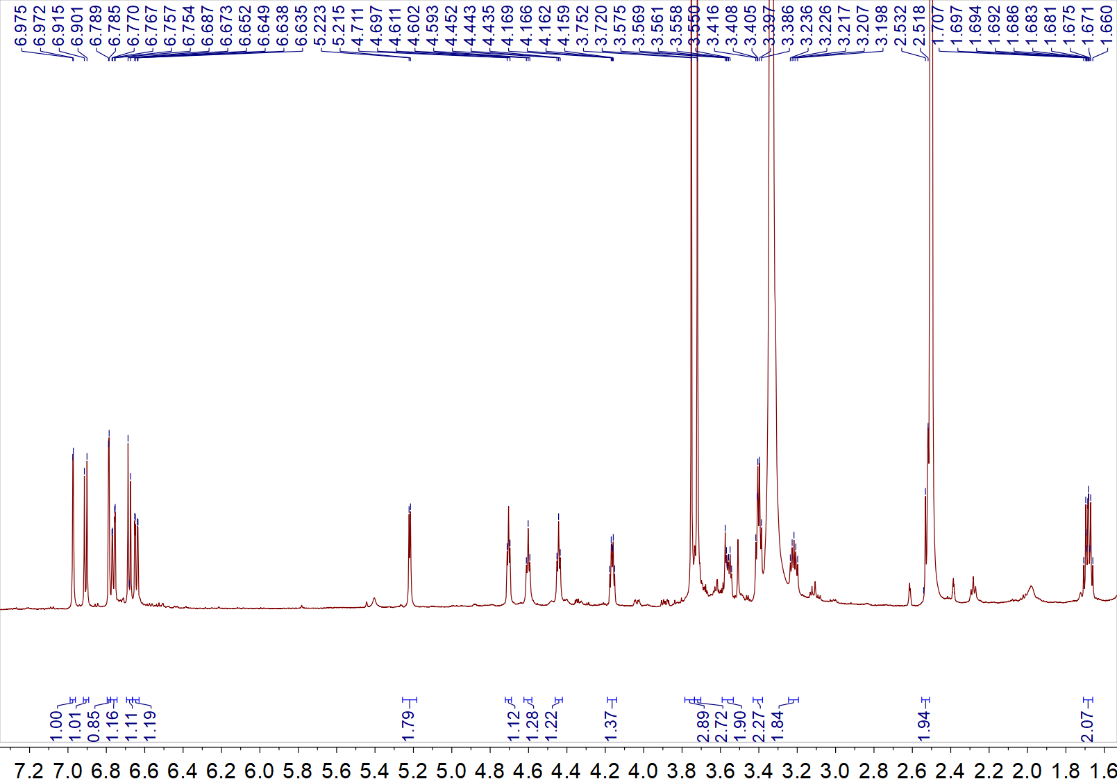


# Figure S64. ^1^H NMR spectrum of compound **19** in DMSO-*d*_6_


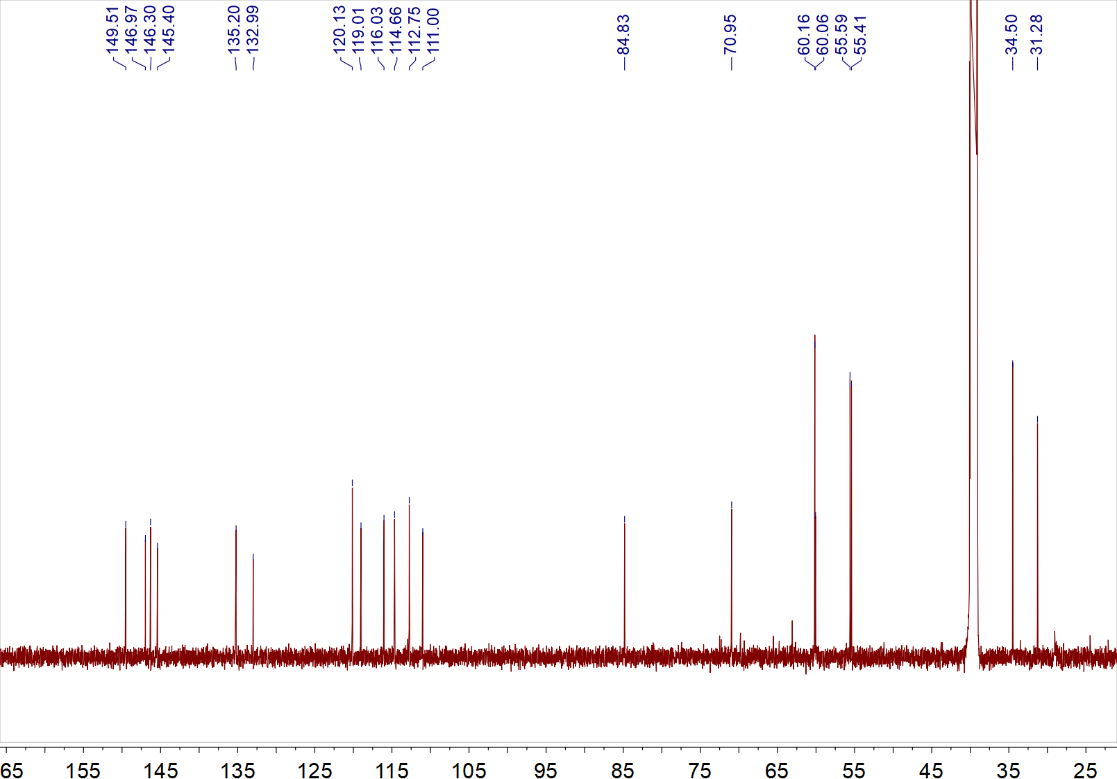


# Figure S65. ^13^C NMR spectrum of compound **19** in DMSO-*d*_6_


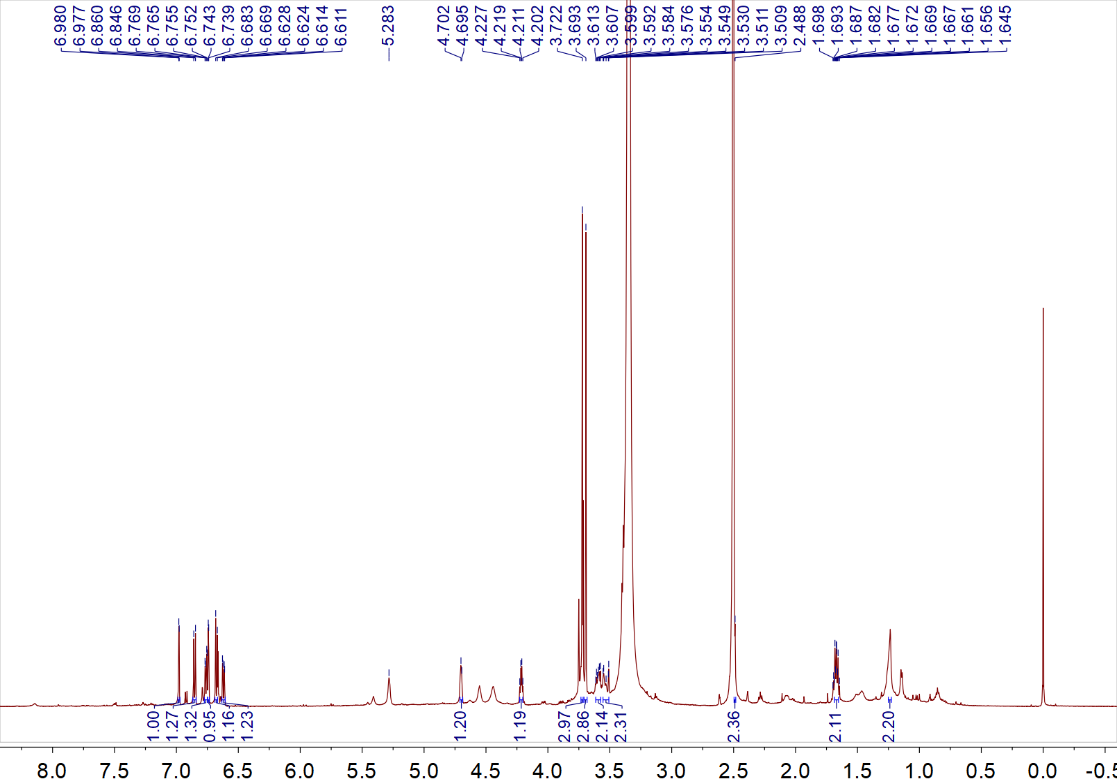


# Figure S66. ^1^H NMR spectrum of compound **20** in DMSO-*d*_6_


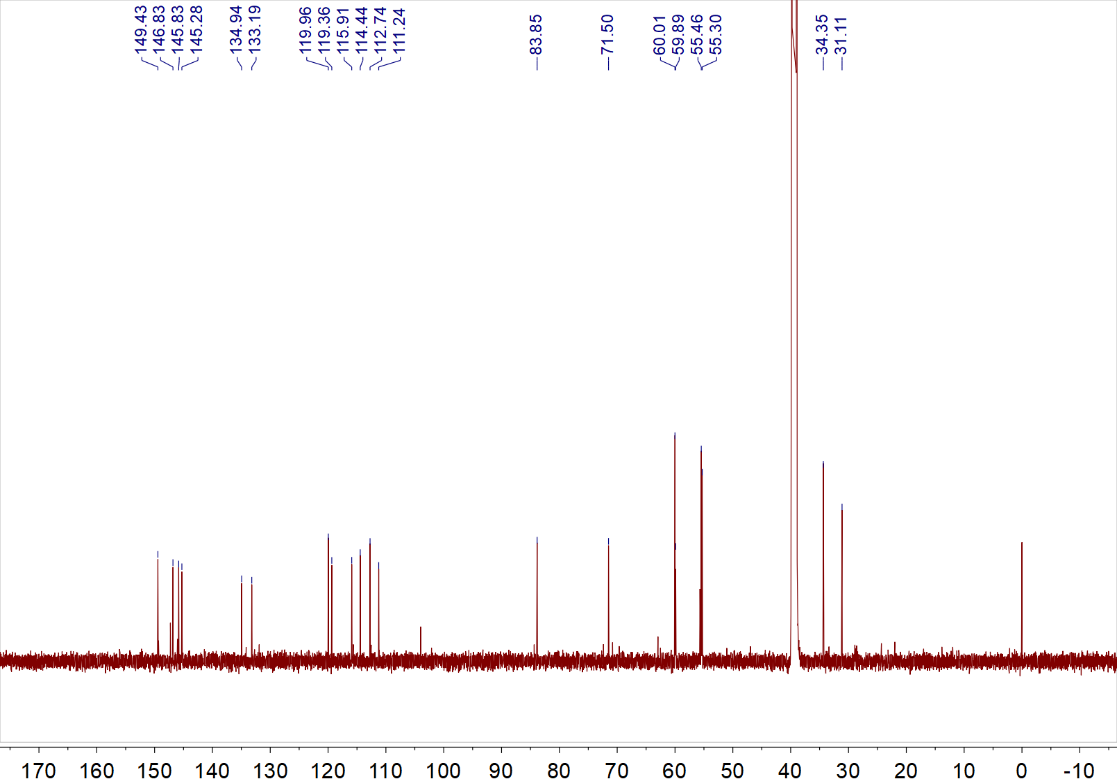


# Figure S67. ^13^C NMR spectrum of compound **20** in DMSO-*d*_6_


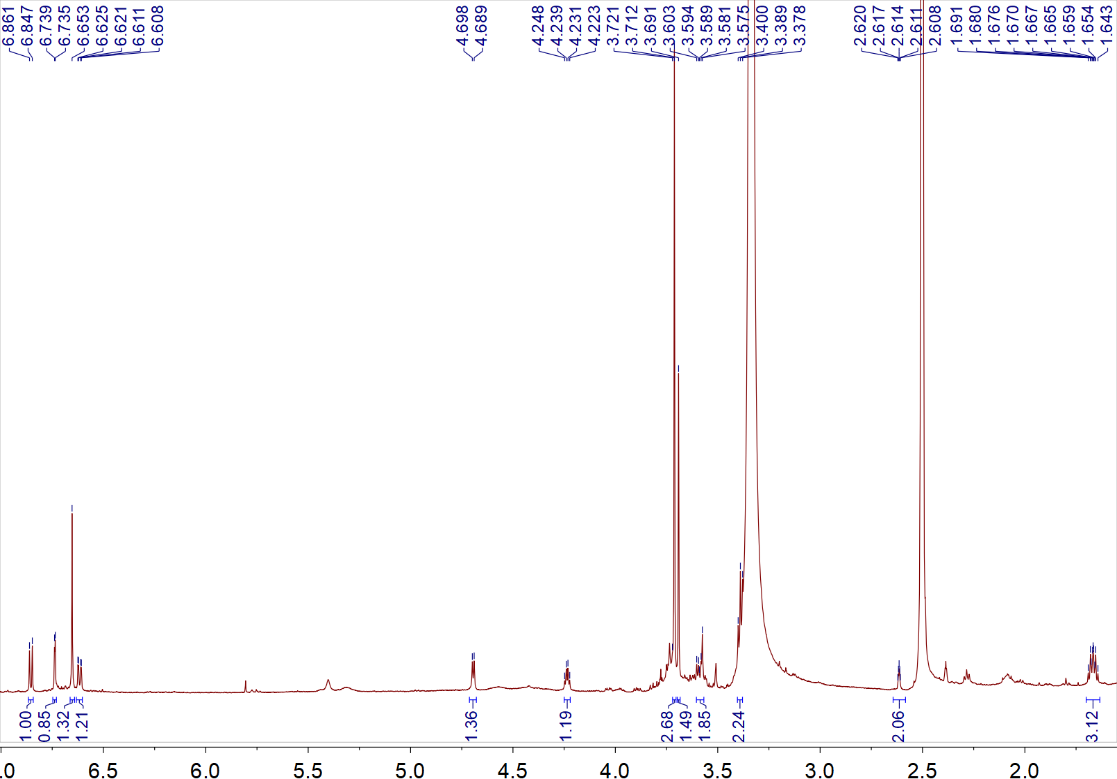


# Figure S68. ^1^H NMR spectrum of compound **21** in DMSO-*d*_6_


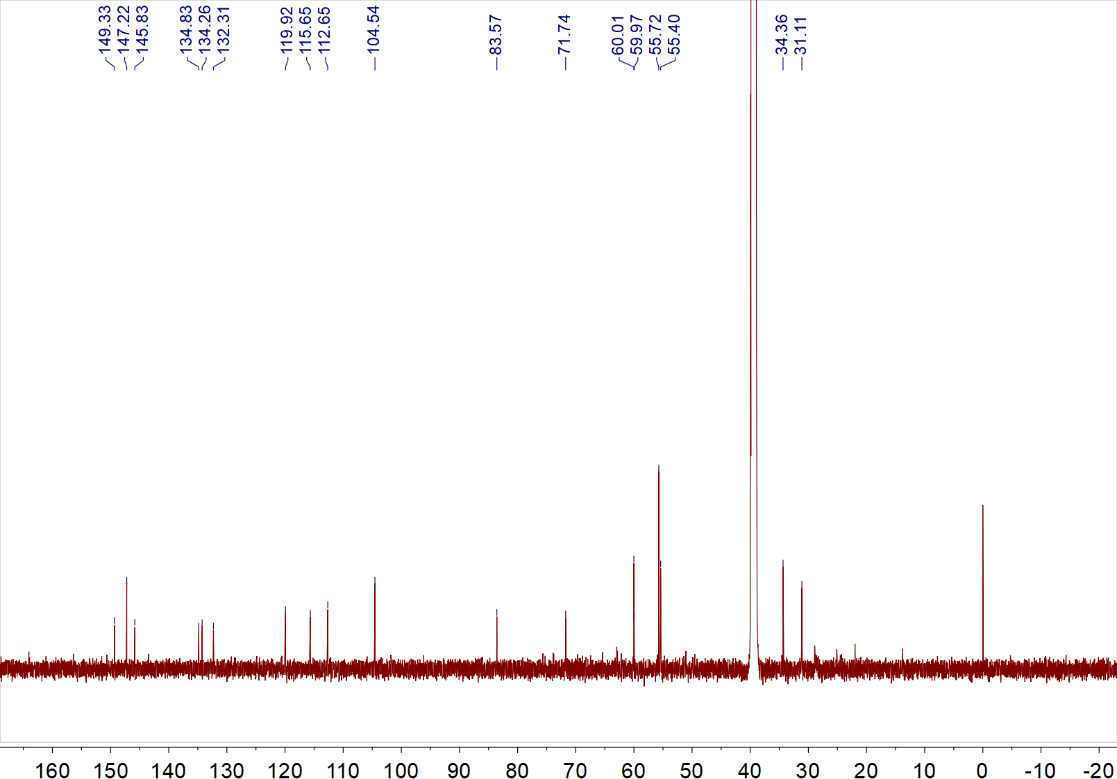


# Figure S69. ^13^C NMR spectrum of compound **21** in DMSO-*d*_6_


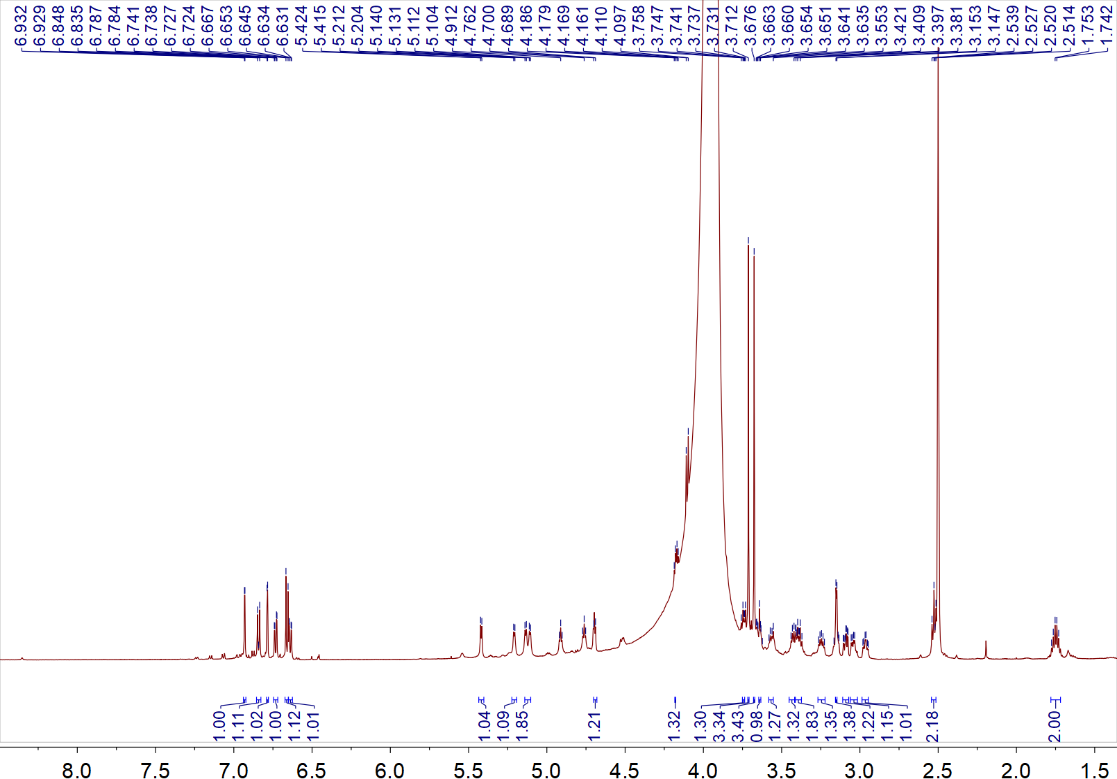


# Figure S70. ^1^H NMR spectrum of compound **22** in DMSO-*d*_6_


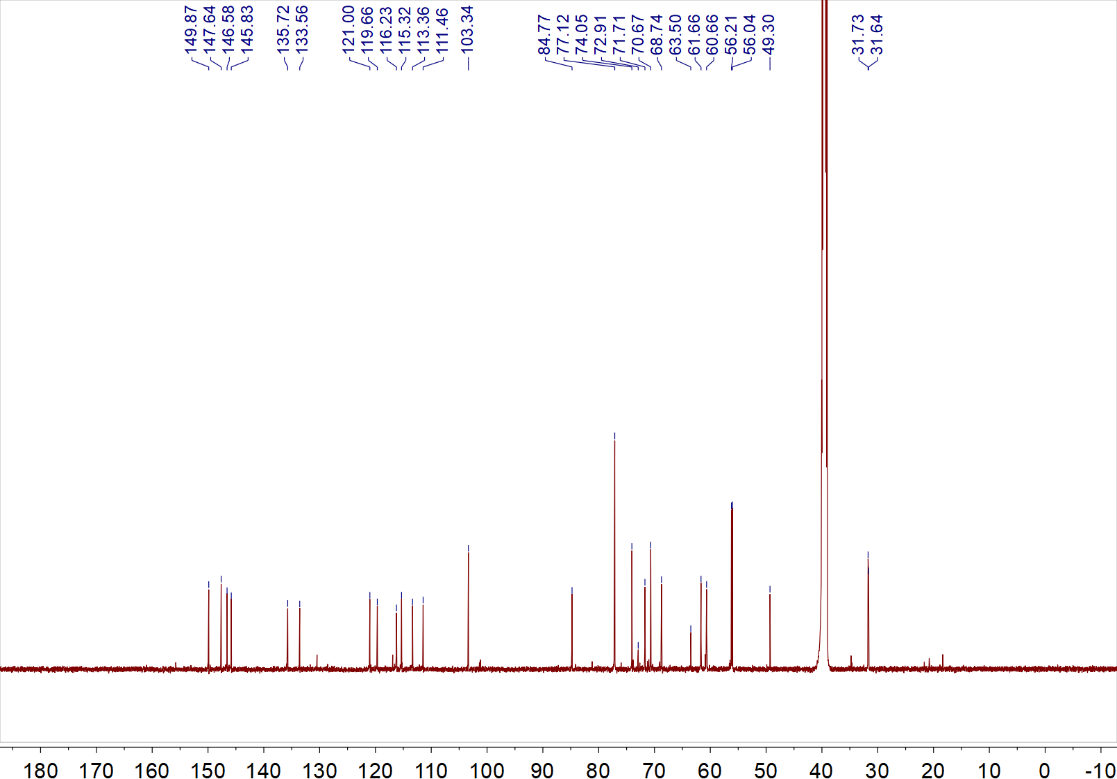


# Figure S71. ^13^C NMR spectrum of compound **22** in DMSO-*d*_6_


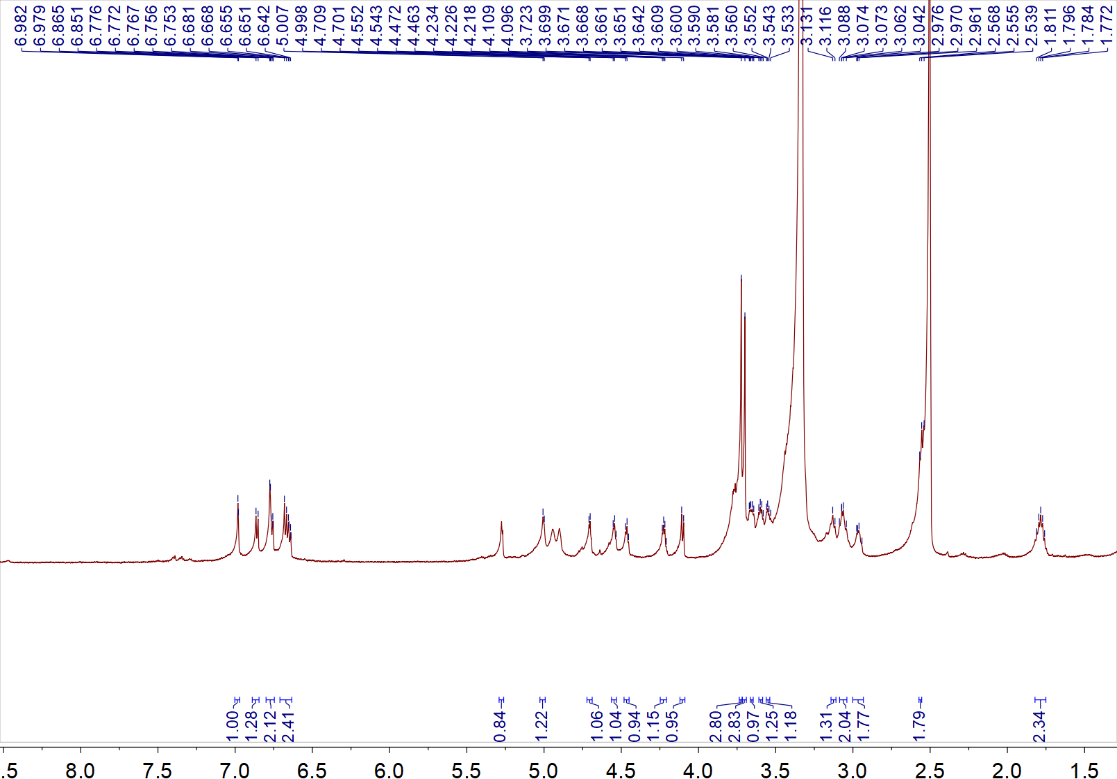


# Figure S72. ^1^H NMR spectrum of compound **23** in DMSO-*d*_6_


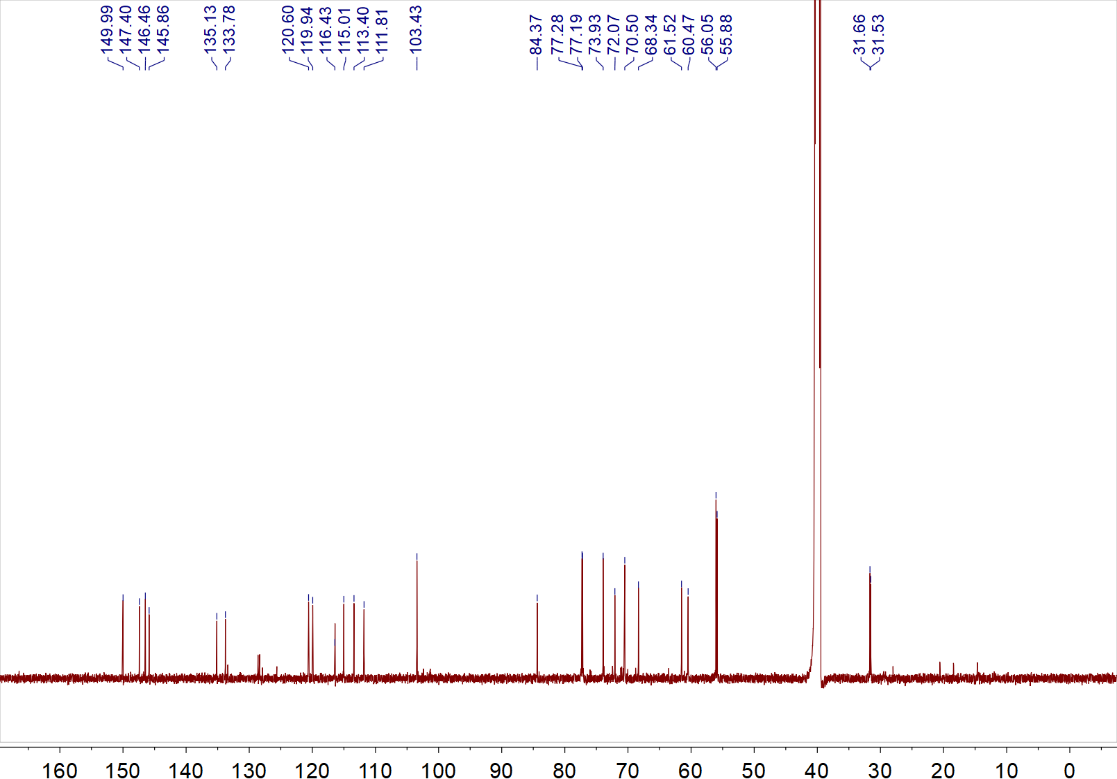


# Figure S73. ^13^C NMR spectrum of compound **23** in DMSO-*d*_6_


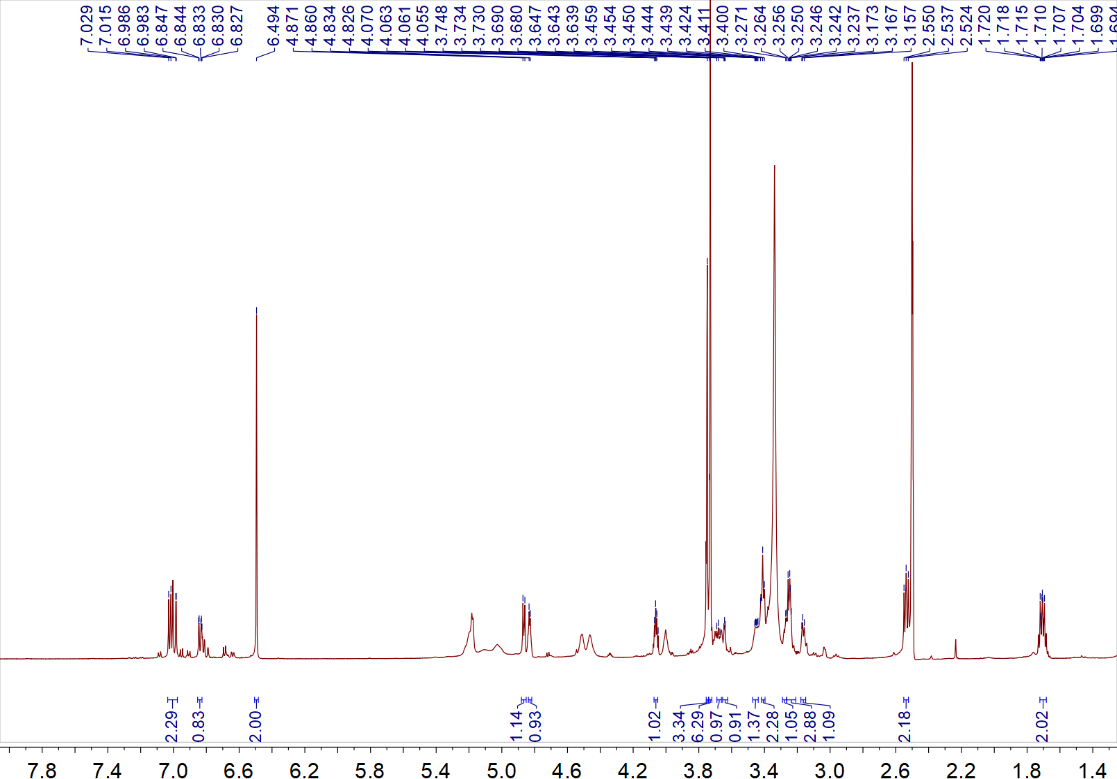


# Figure S74. ^1^H NMR spectrum of compound **24** in DMSO-*d*_6_


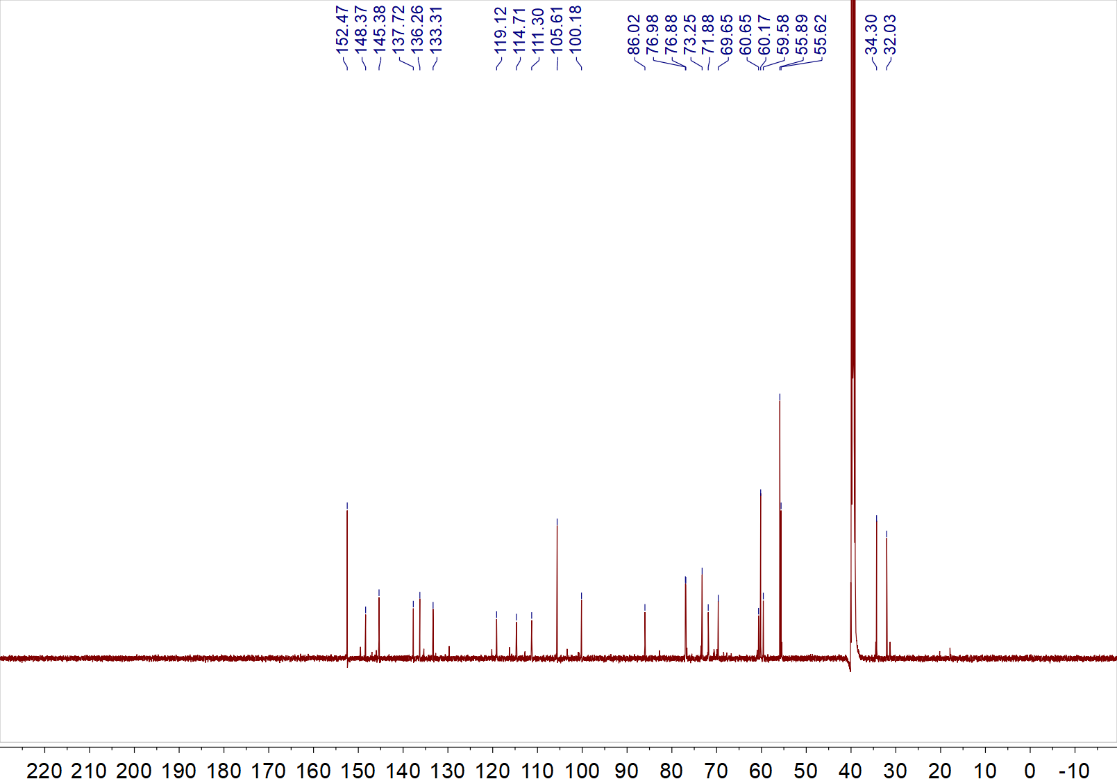


# Figure S75. ^13^C NMR spectrum of compound **24** in DMSO-*d*_6_


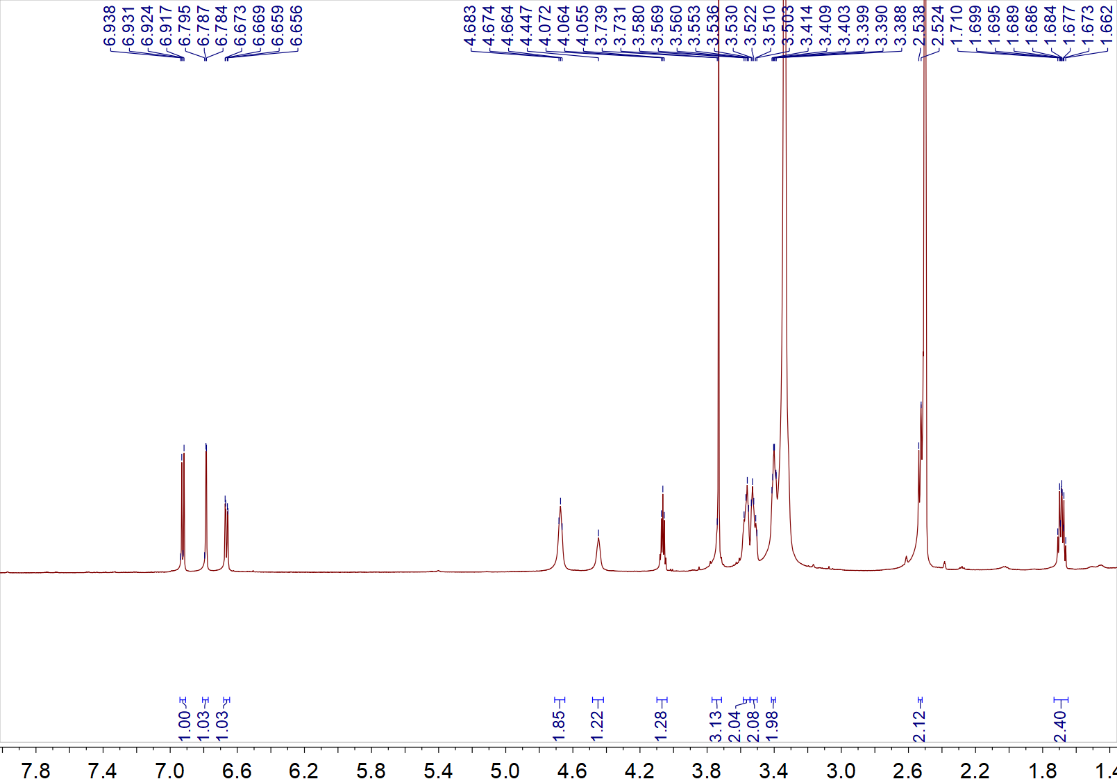


# Figure S76. ^1^H NMR spectrum of compound **25** in DMSO-*d*_6_


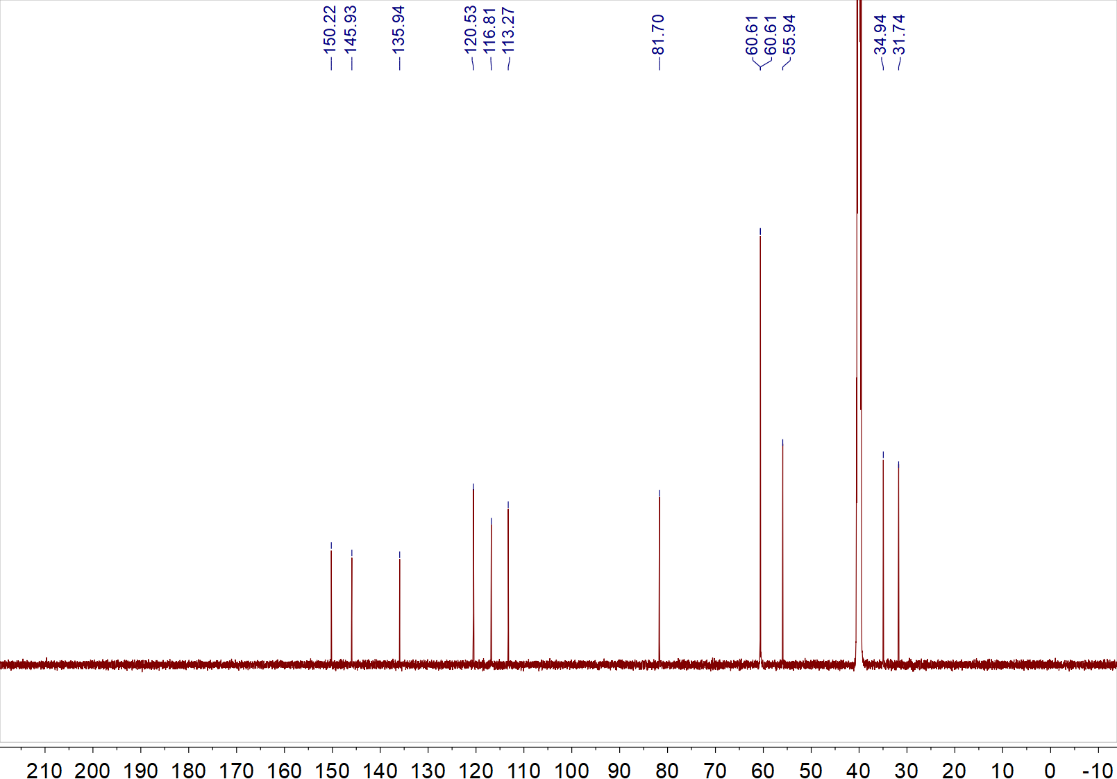


# Figure S77. ^13^C NMR spectrum of compound **25** in DMSO-*d*_6_


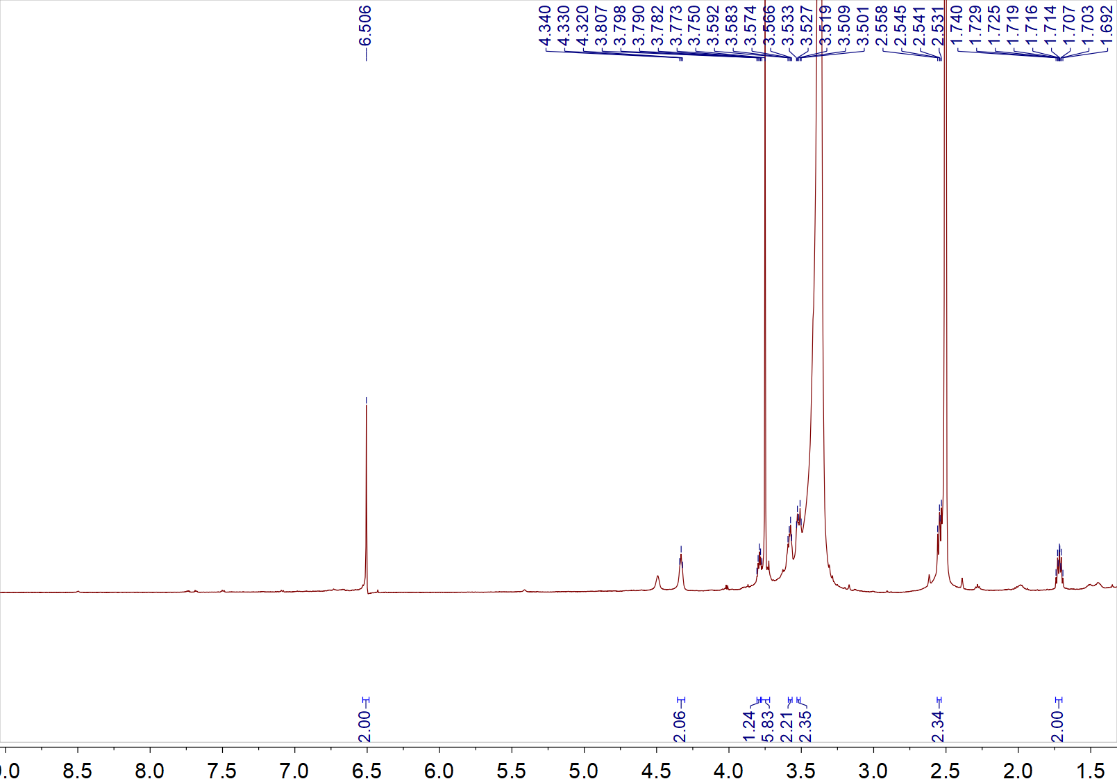


# Figure S78. ^1^H NMR spectrum of compound **26** in DMSO-*d*_6_


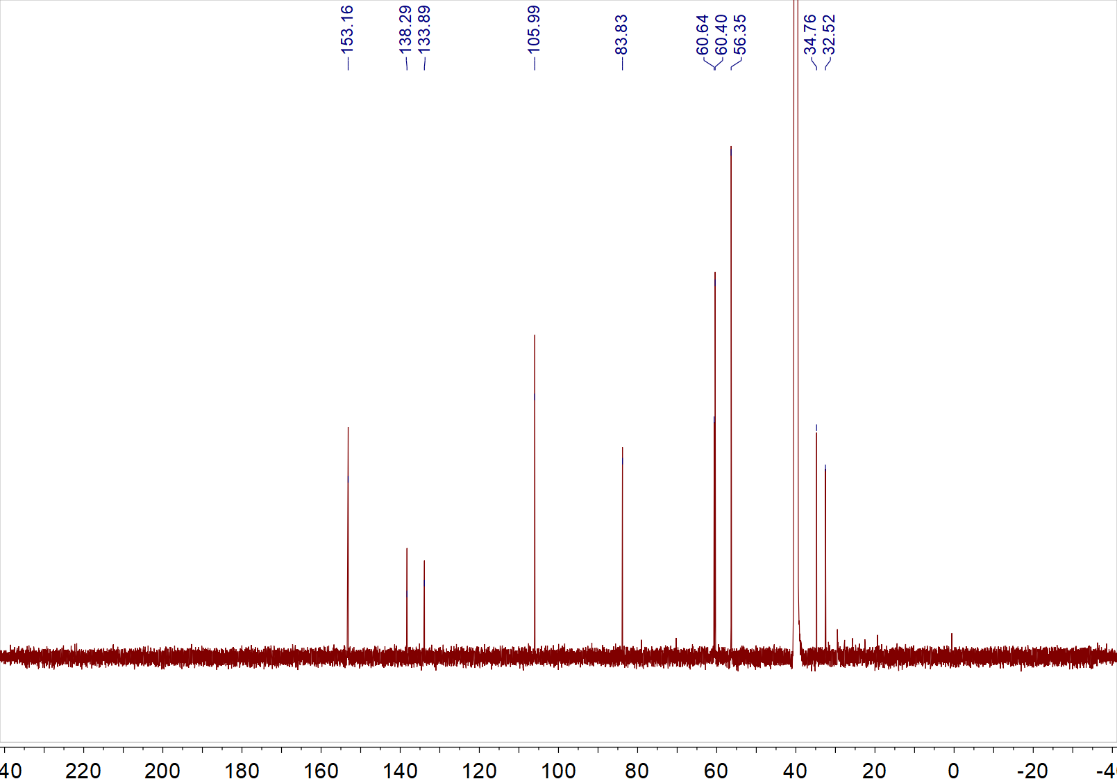


# Figure S79. ^13^C NMR spectrum of compound **26** in DMSO-*d*_6_


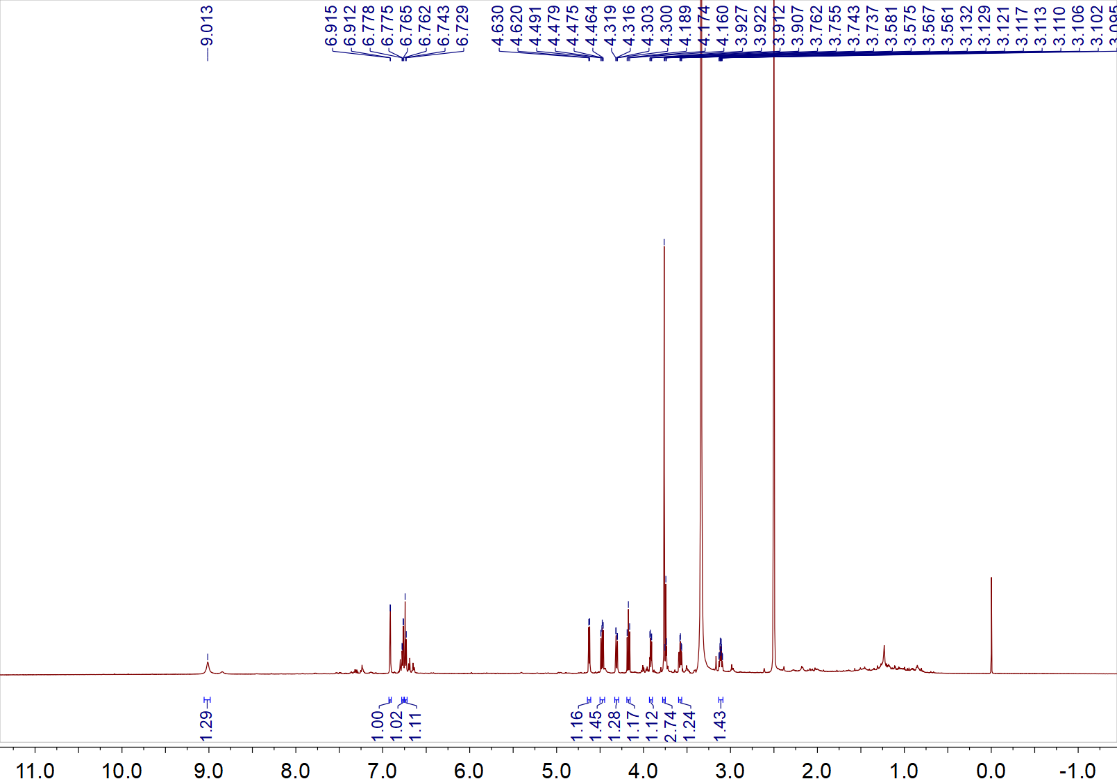


# Figure S80. ^1^H NMR spectrum of compound **27** in DMSO-*d*_6_


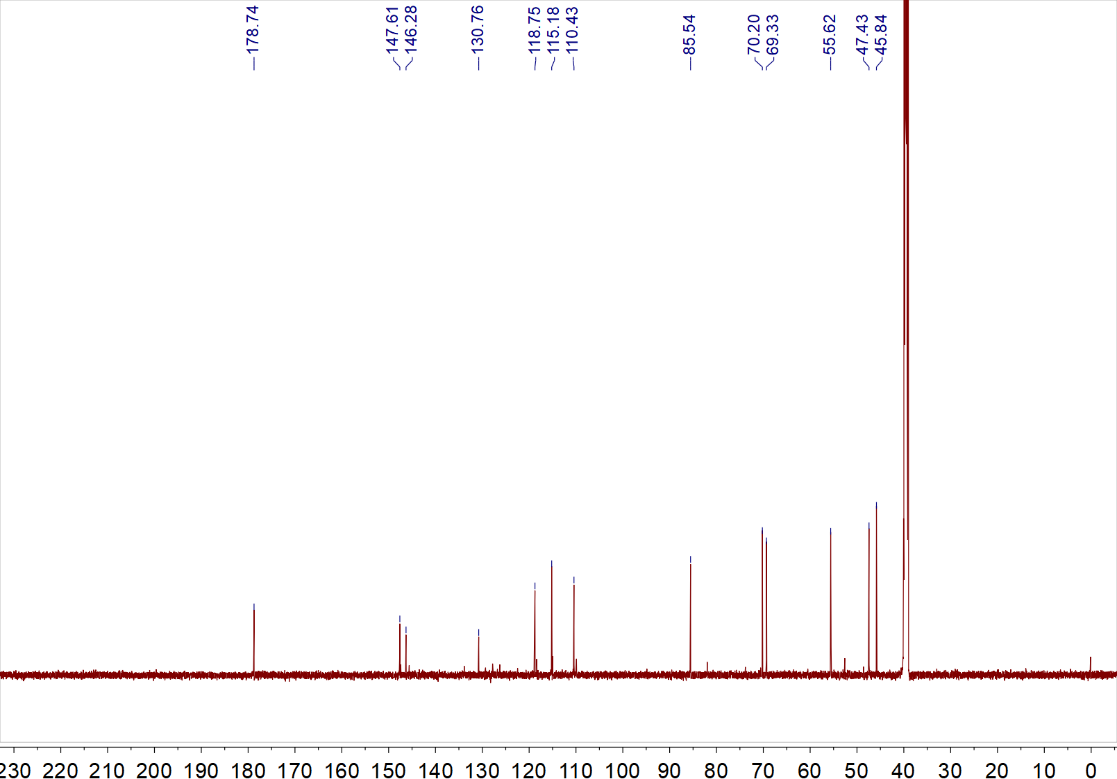


# Figure S81. ^13^C NMR spectrum of compound **27** in DMSO-*d*_6_


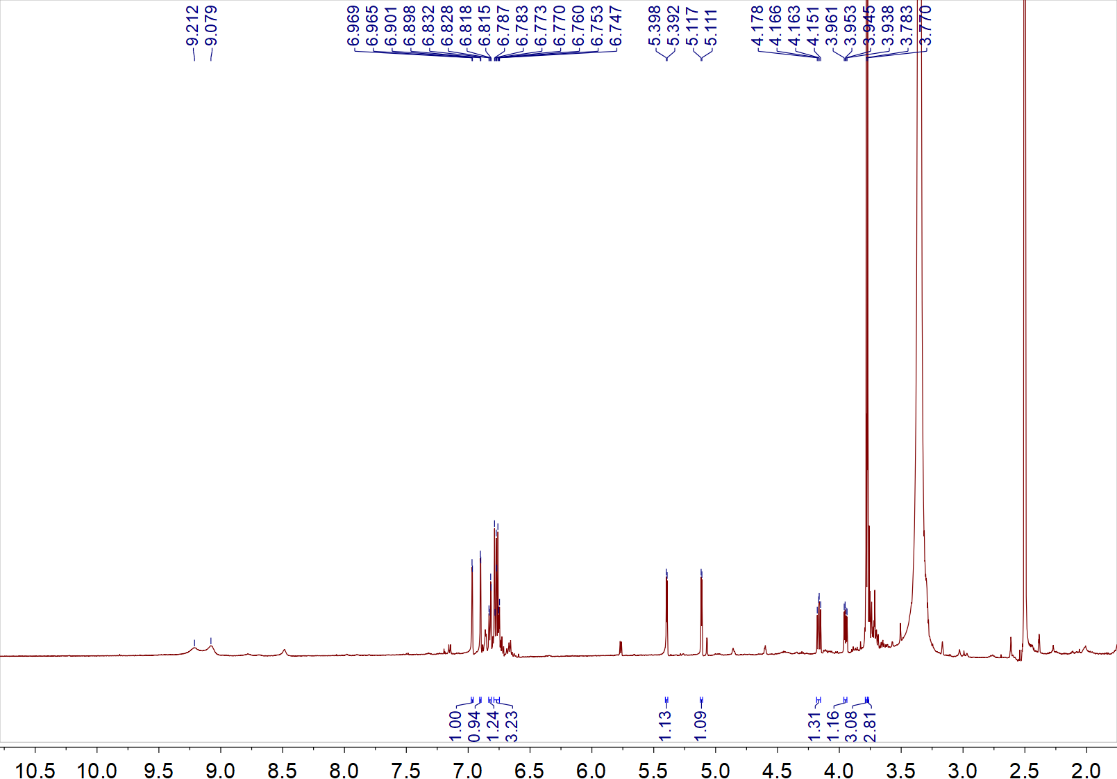


# Figure S82. ^1^H NMR spectrum of compound **28** in DMSO-*d*_6_


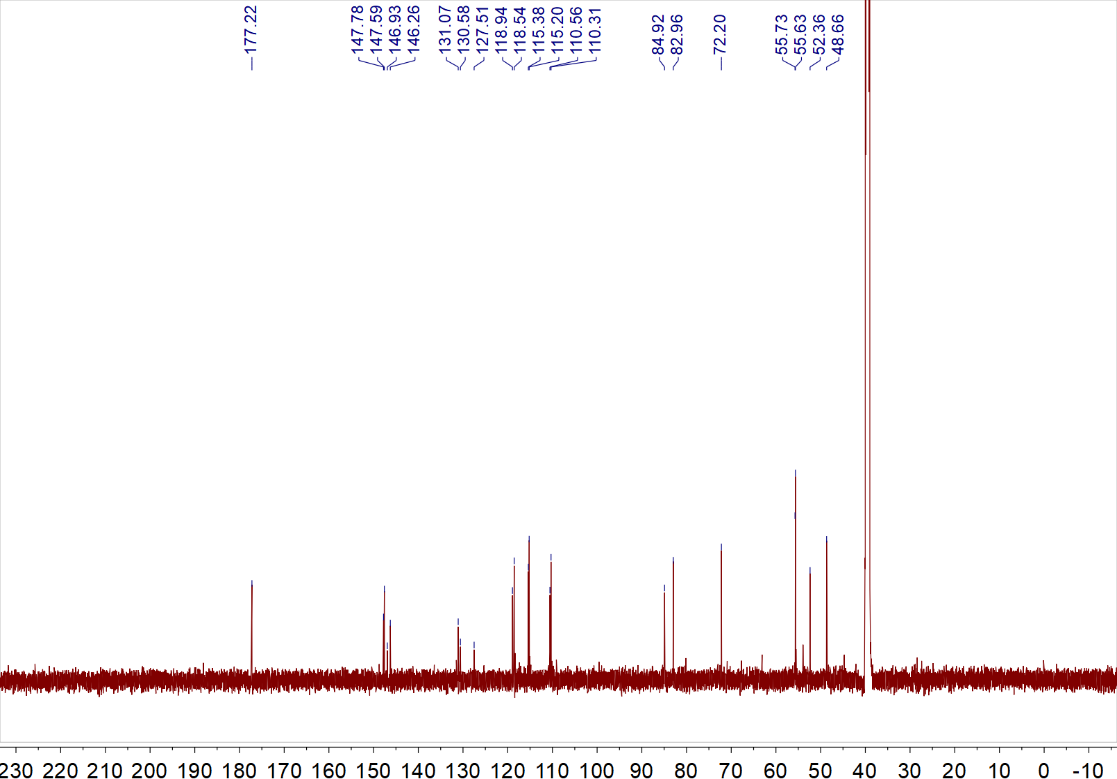


# Figure S83. ^13^C NMR spectrum of compound **28** in DMSO-*d*_6_


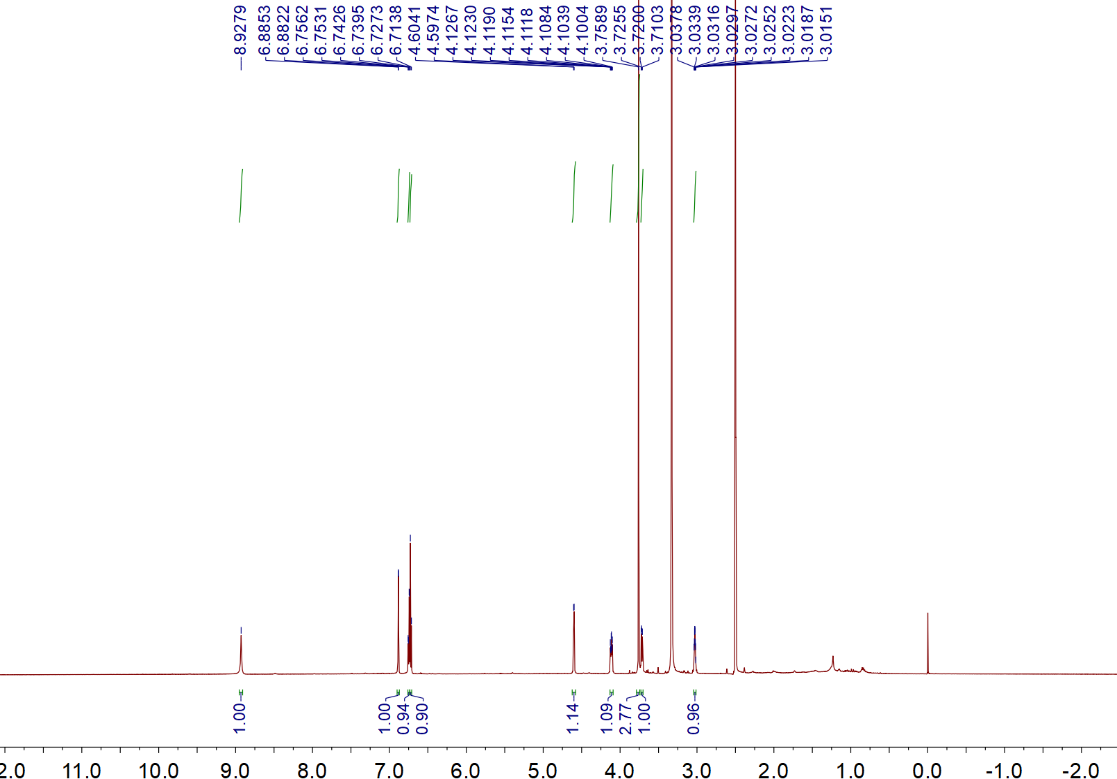


# Figure S84. ^1^H NMR spectrum of compound **29** in DMSO-*d*_6_


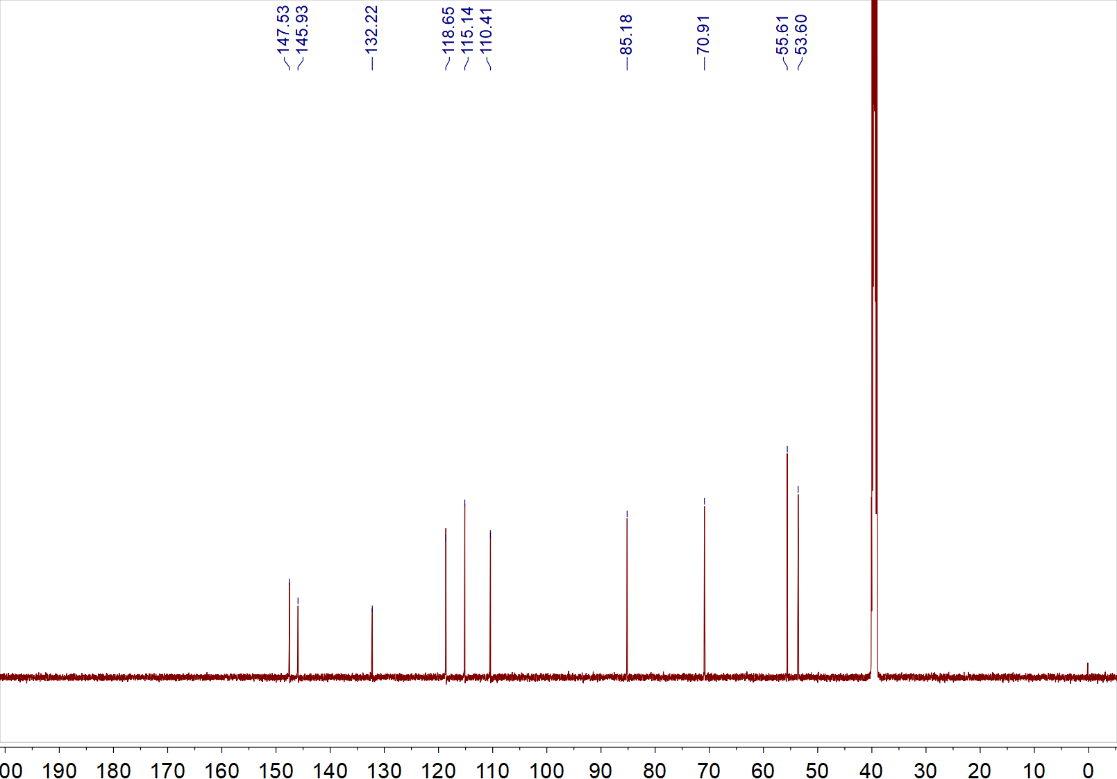


# Figure S85. ^13^C NMR spectrum of compound **29** in DMSO-*d*_6_


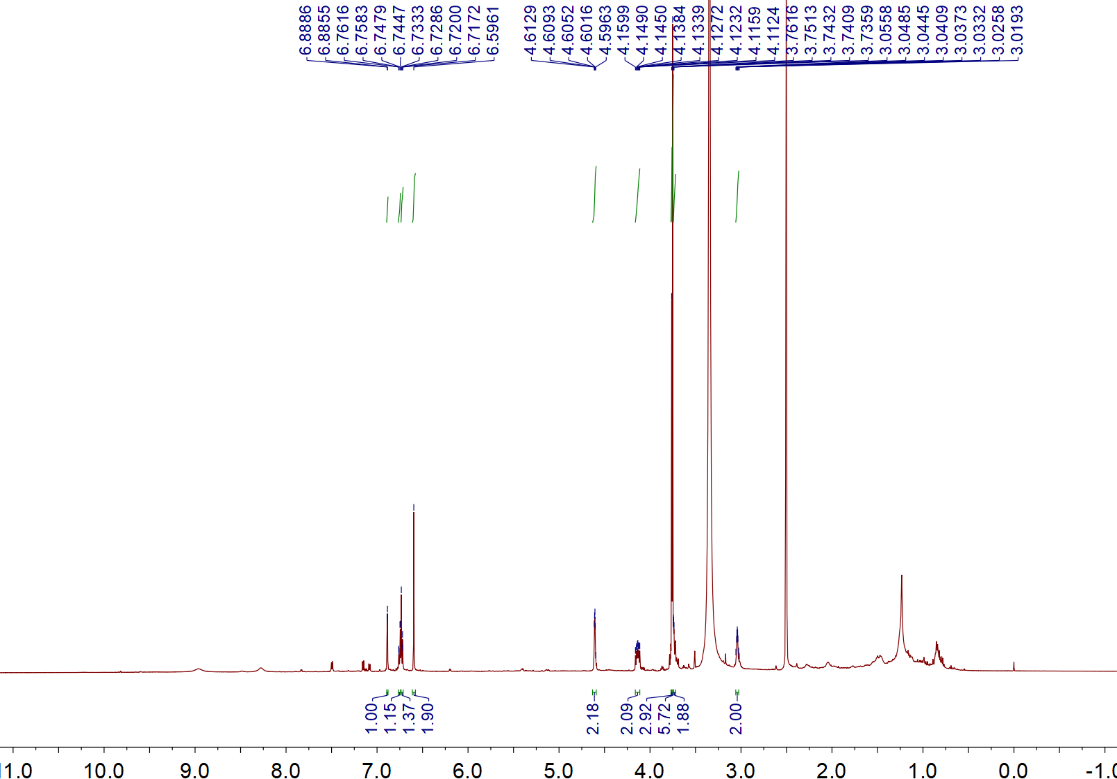


# Figure S86. ^1^H NMR spectrum of compound **30** in DMSO-*d*_6_


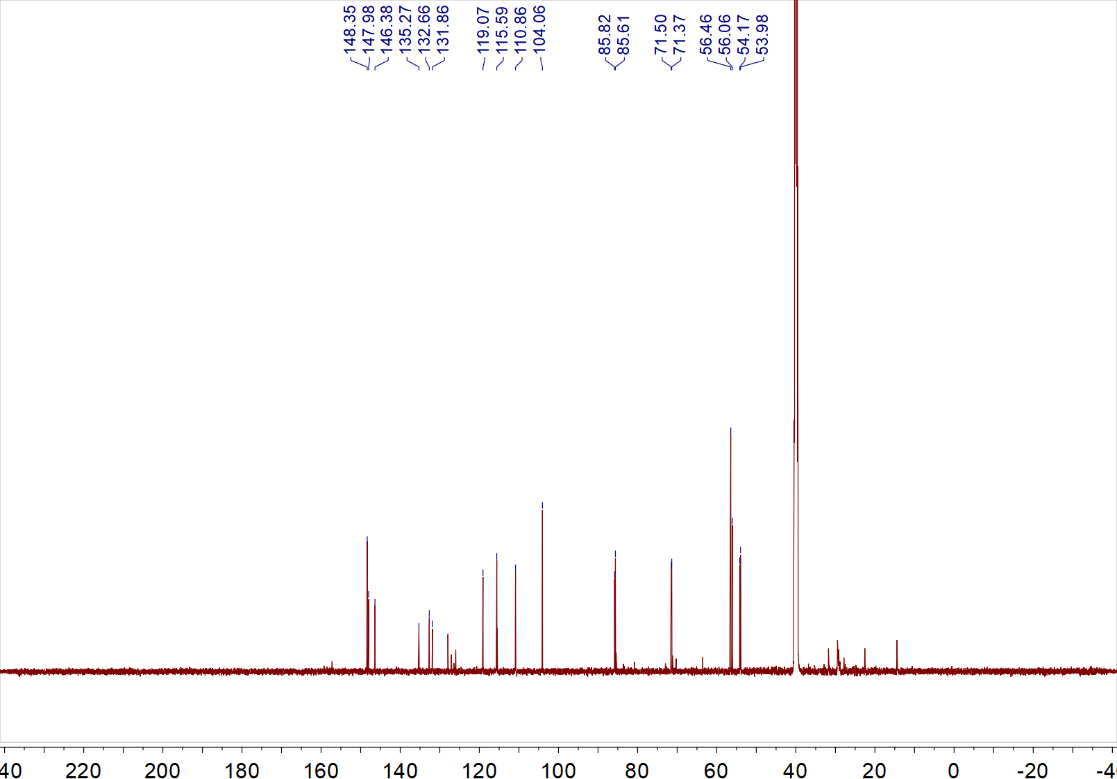


# Figure S87. ^13^C NMR spectrum of compound **30** in DMSO-*d*_6_


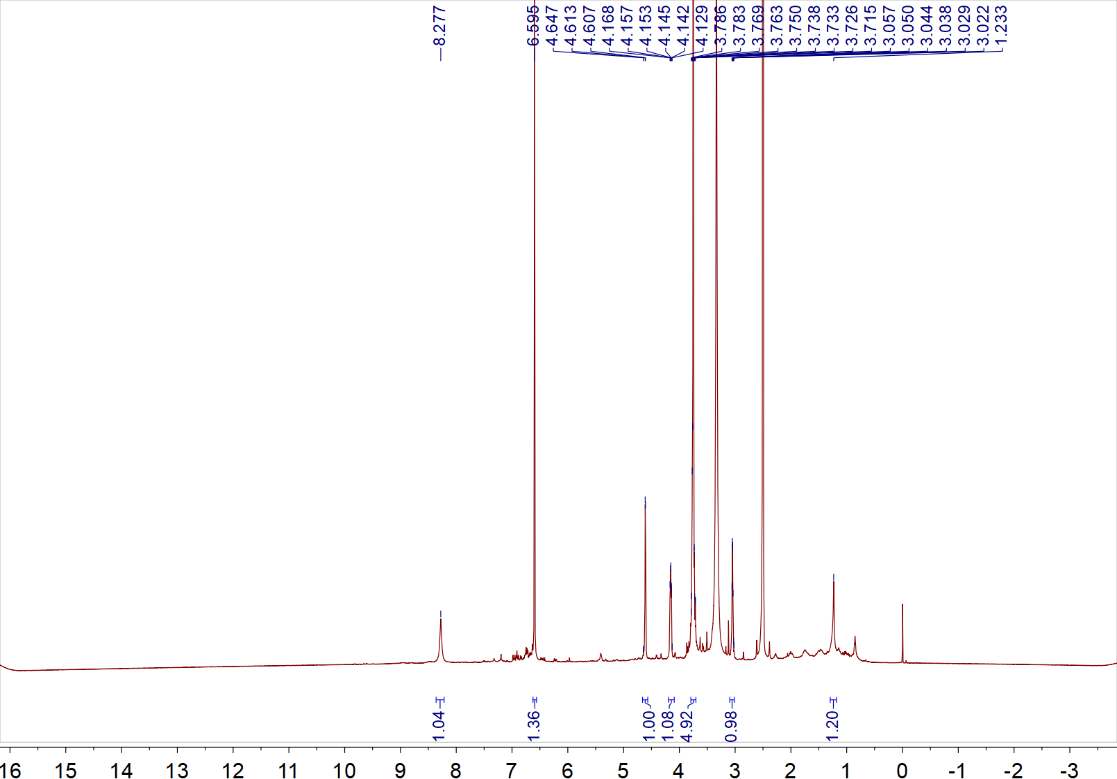


# Figure S88. ^1^H NMR spectrum of compound **31** in DMSO-*d*_6_


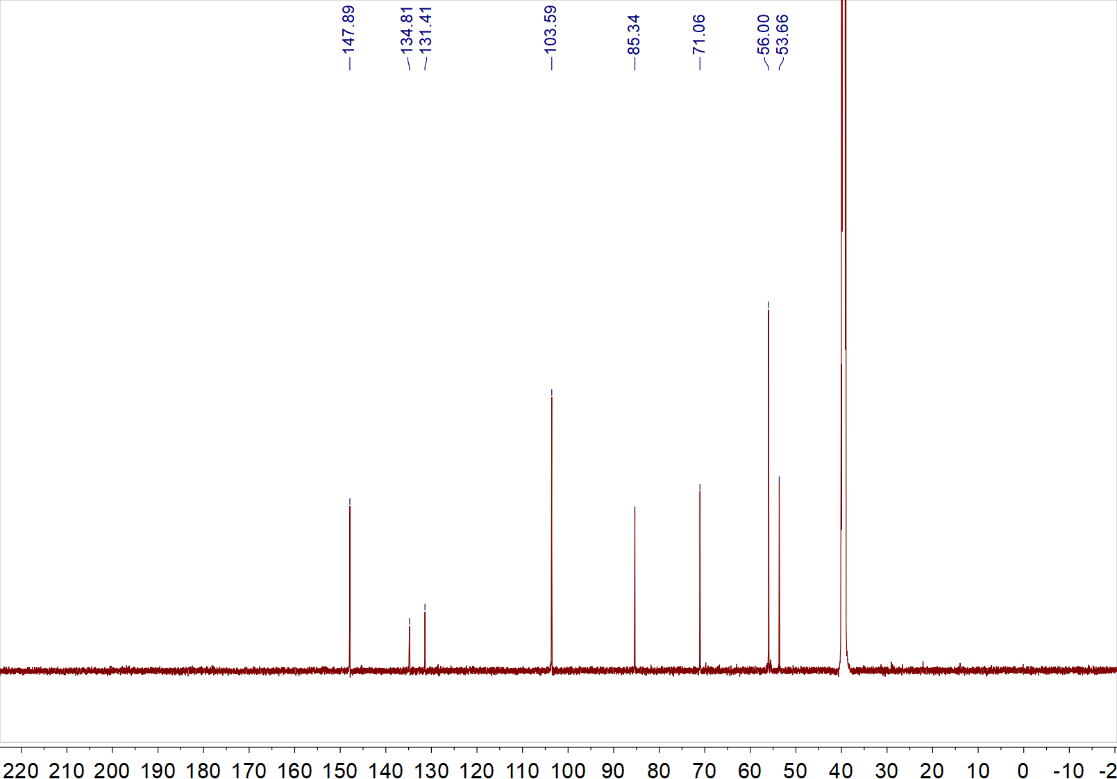


# Figure S89. ^13^C NMR spectrum of compound **31** in DMSO-*d*_6_


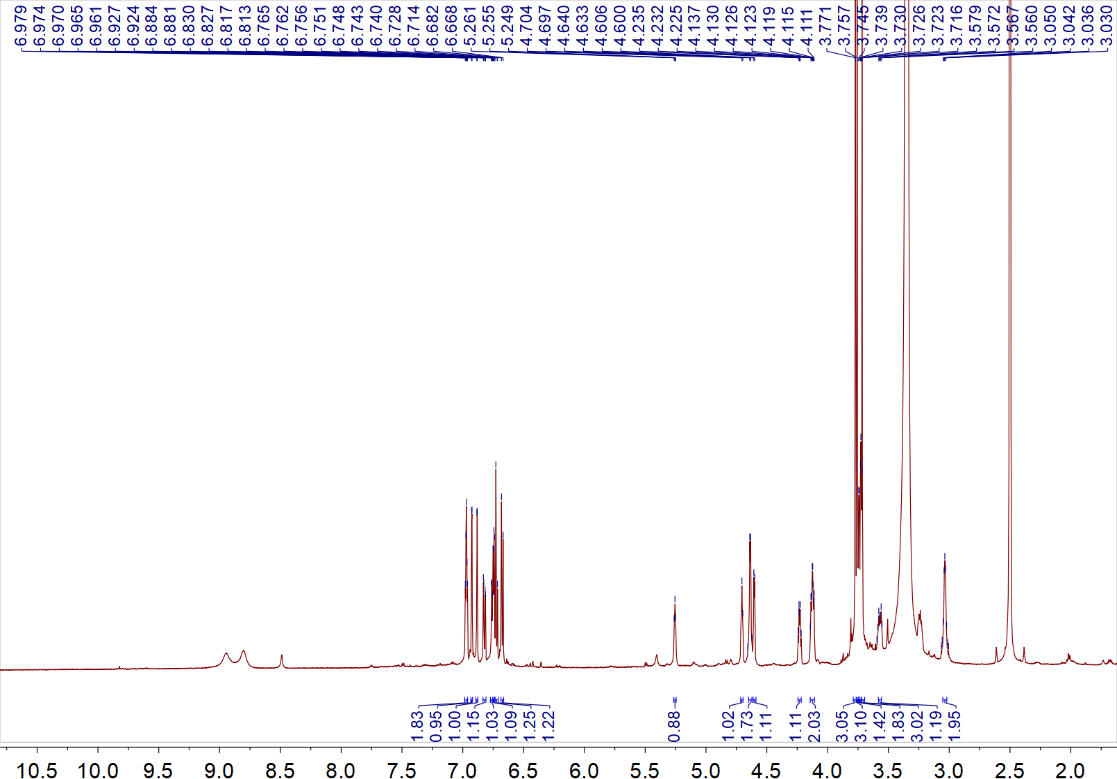


# Figure S90. ^1^H NMR spectrum of compound **32** in DMSO-*d*_6_


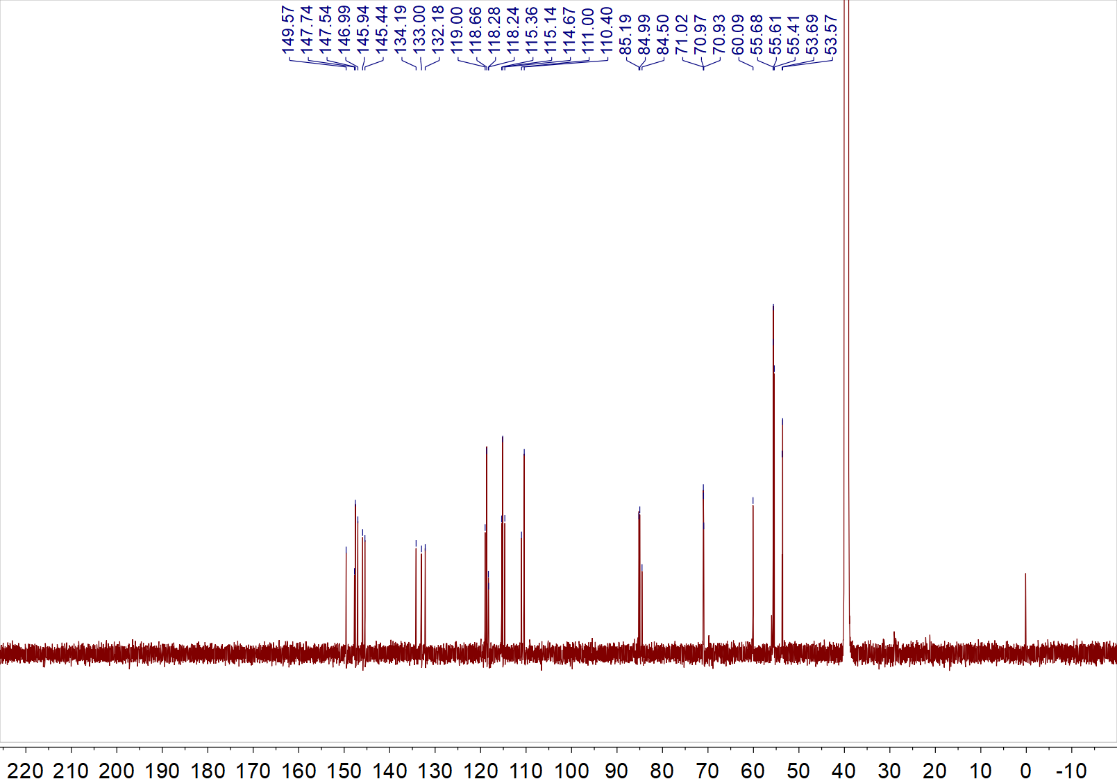


# Figure S91. ^13^C NMR spectrum of compound **32** in DMSO-*d*_6_


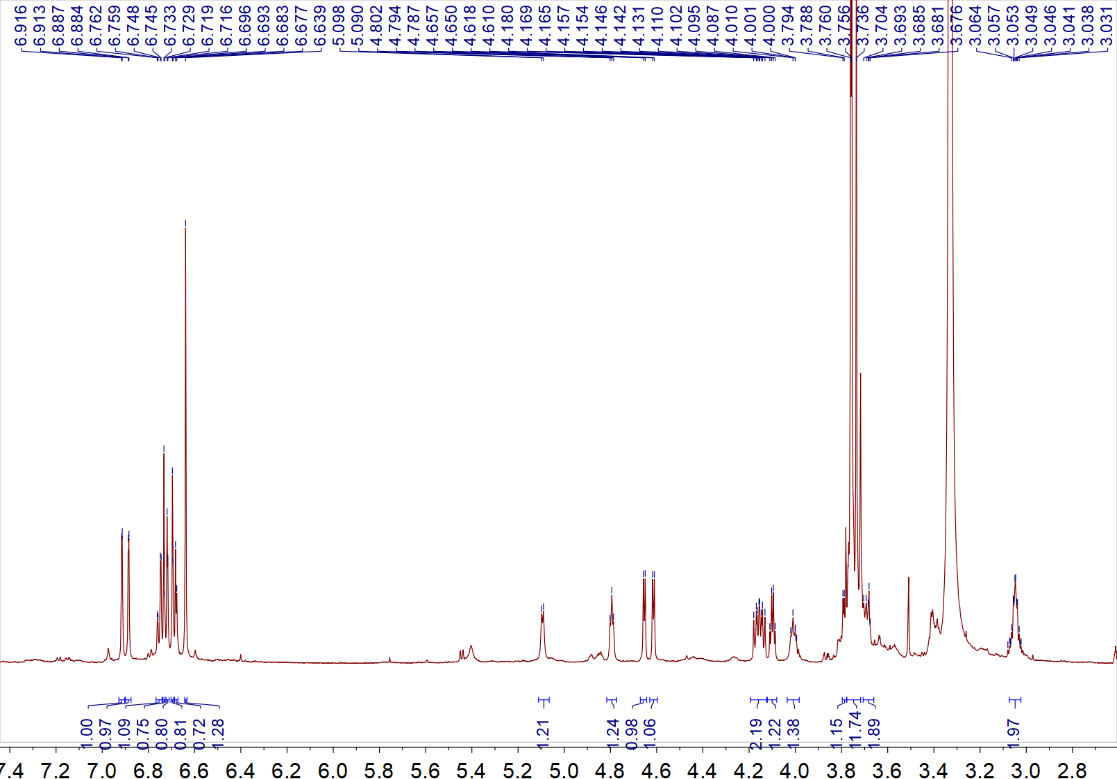


# Figure S92. ^1^H NMR spectrum of compound **33** in DMSO-*d*_6_


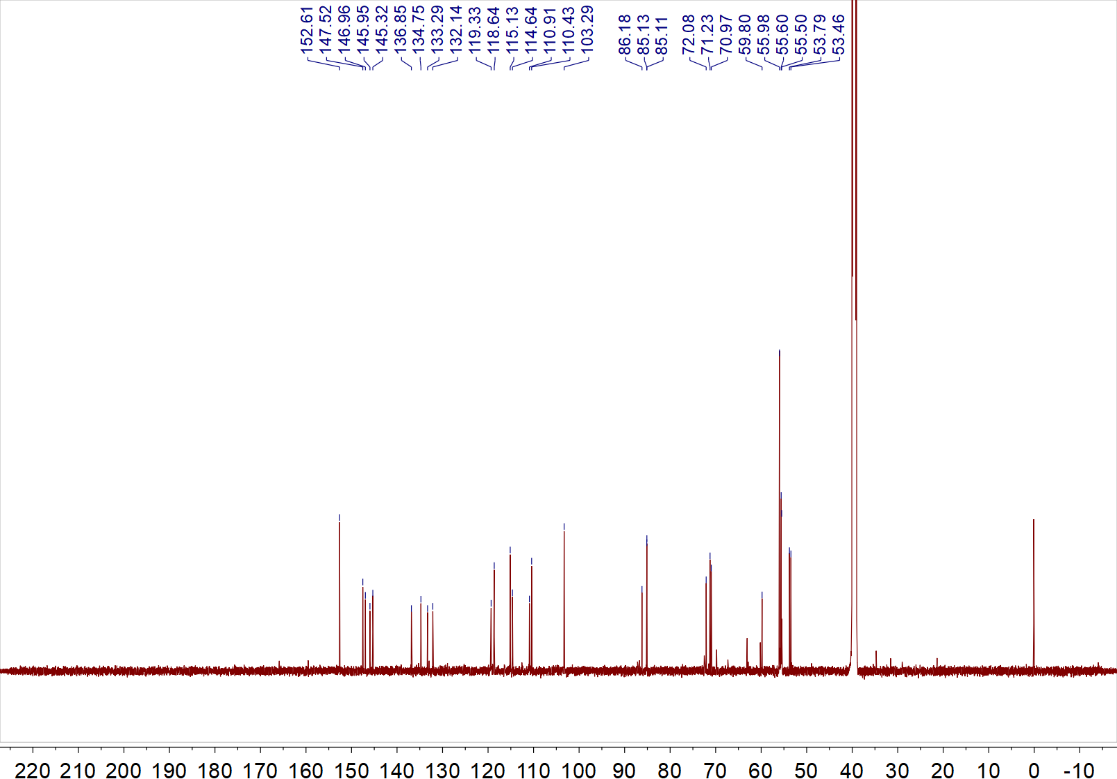


# Figure S93. ^13^C NMR spectrum of compound **33** in DMSO-*d*_6_


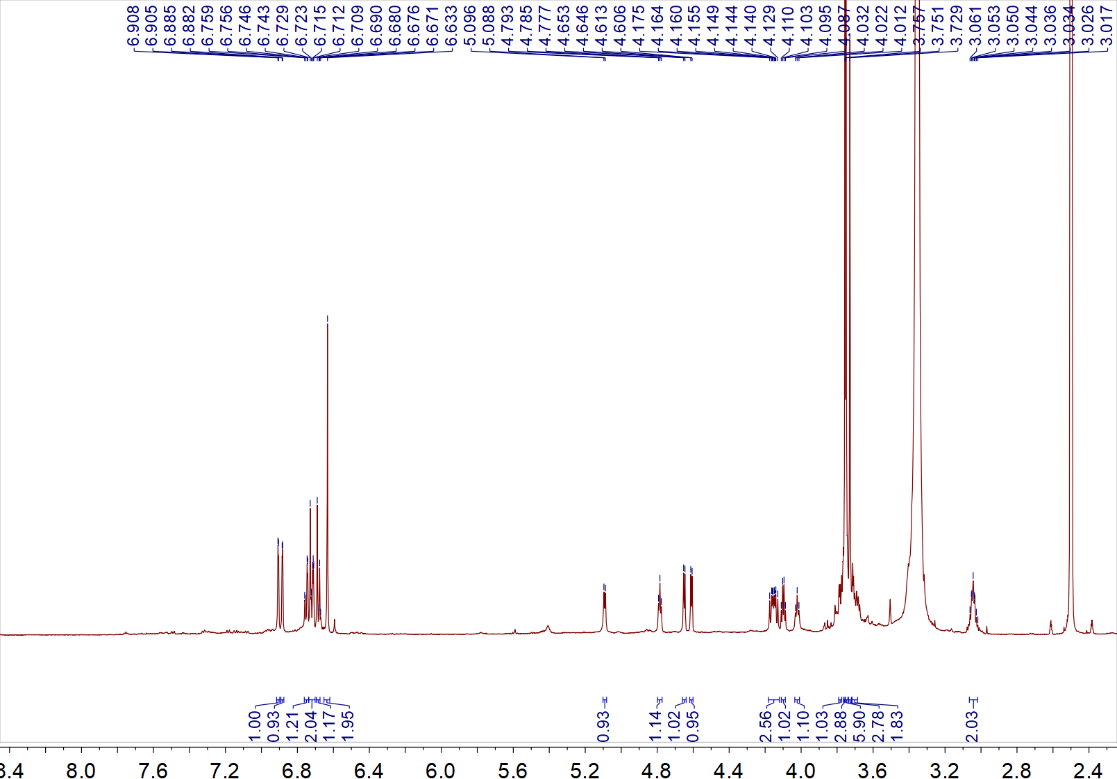


# Figure S94. ^1^H NMR spectrum of compound **34** in DMSO-*d*_6_


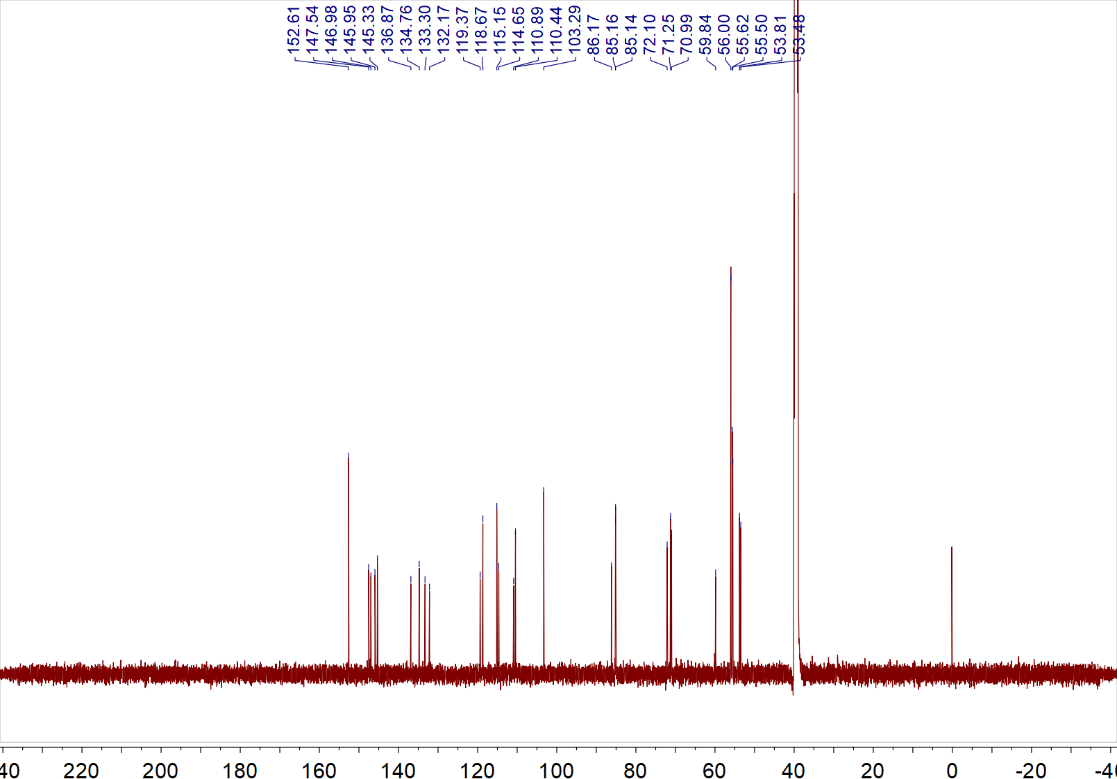


# Figure S95. ^13^C NMR spectrum of compound **34** in DMSO-*d*_6_


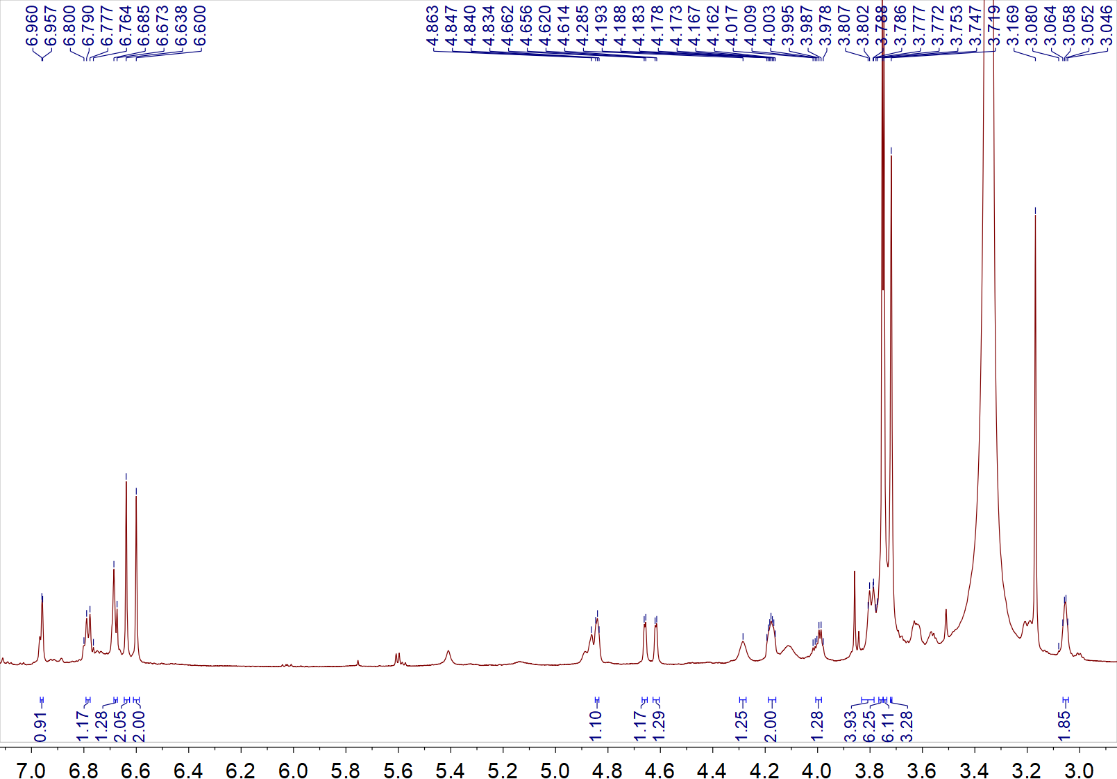


# Figure S96. ^1^H NMR spectrum of compound **35** in DMSO-*d*_6_


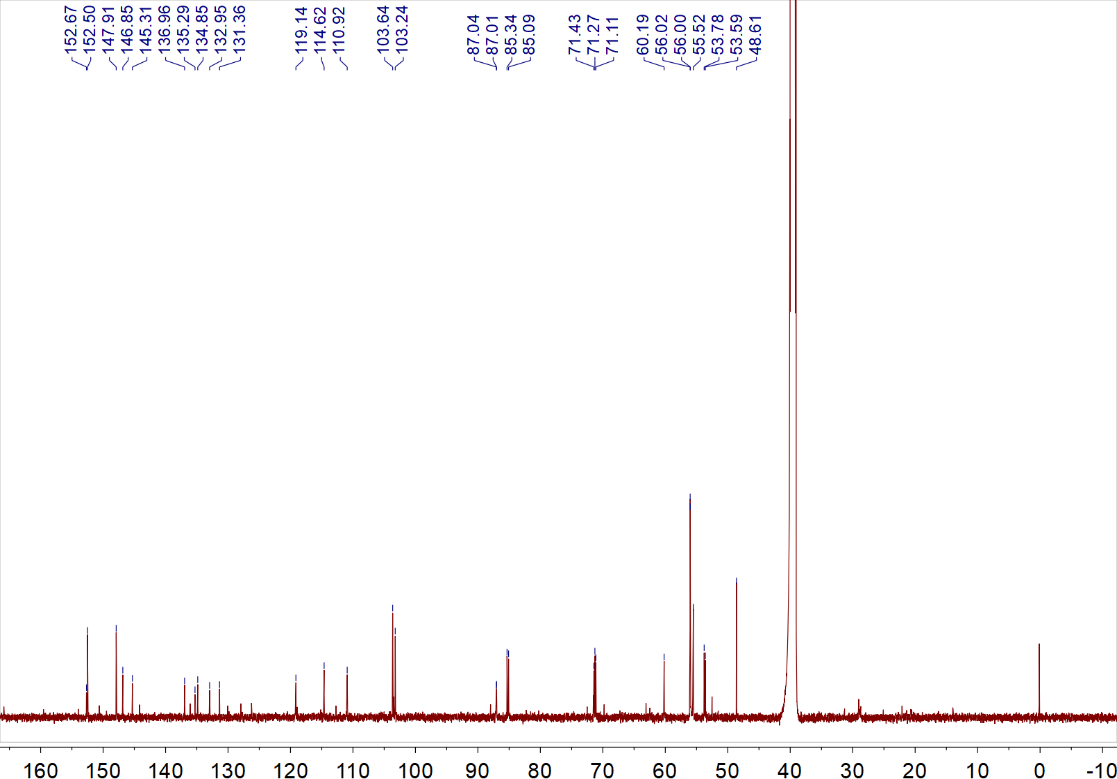


# Figure S97. ^13^C NMR spectrum of compound **35** in DMSO-*d*_6_


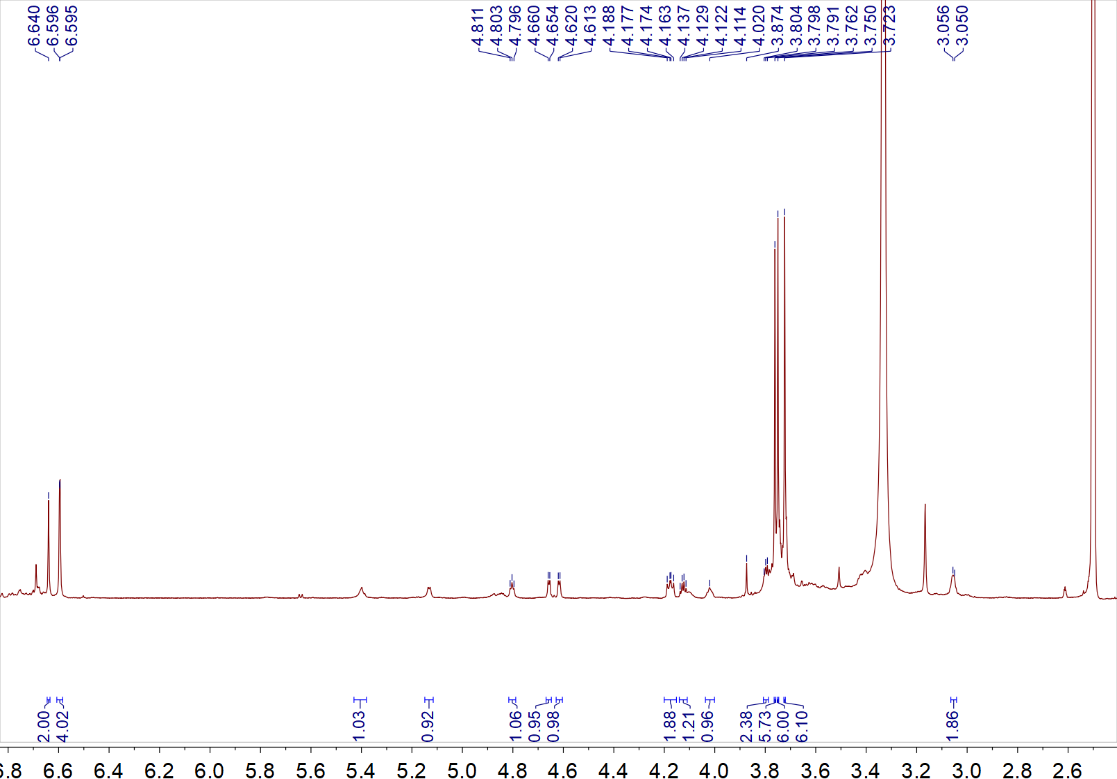


# Figure S98. ^1^H NMR spectrum of compound **36** in DMSO-*d*_6_


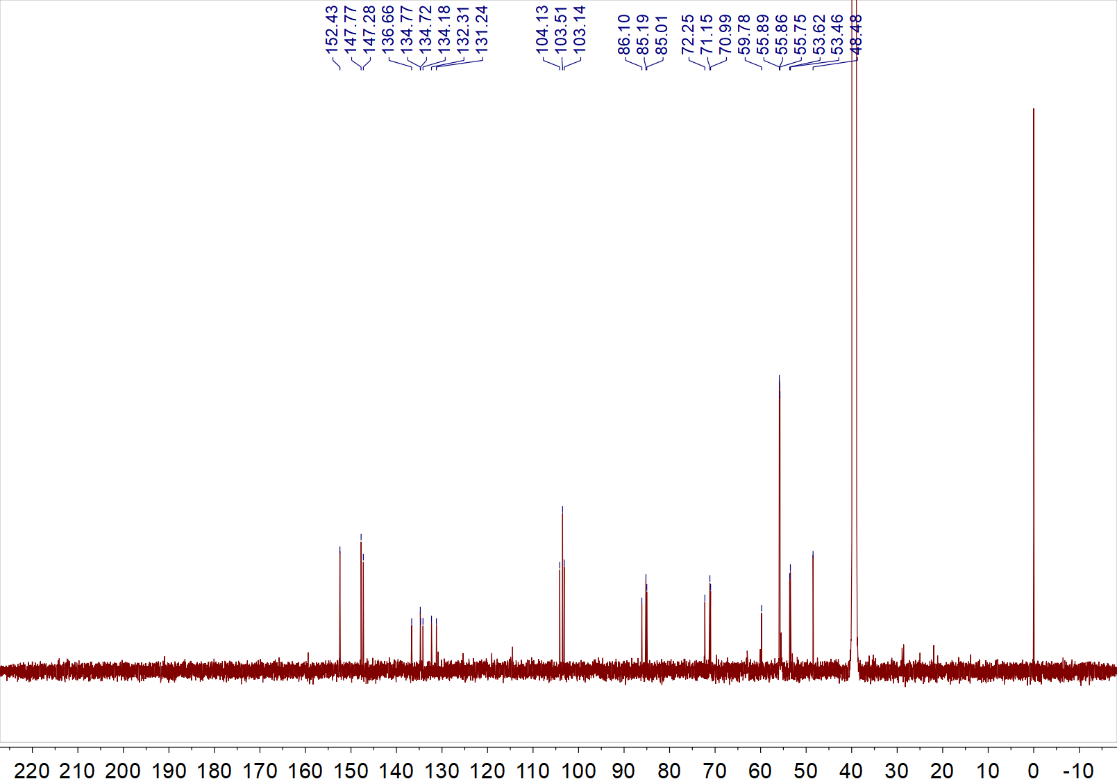


# Figure S99. ^13^C NMR spectrum of compound **36** in DMSO-*d*_6_


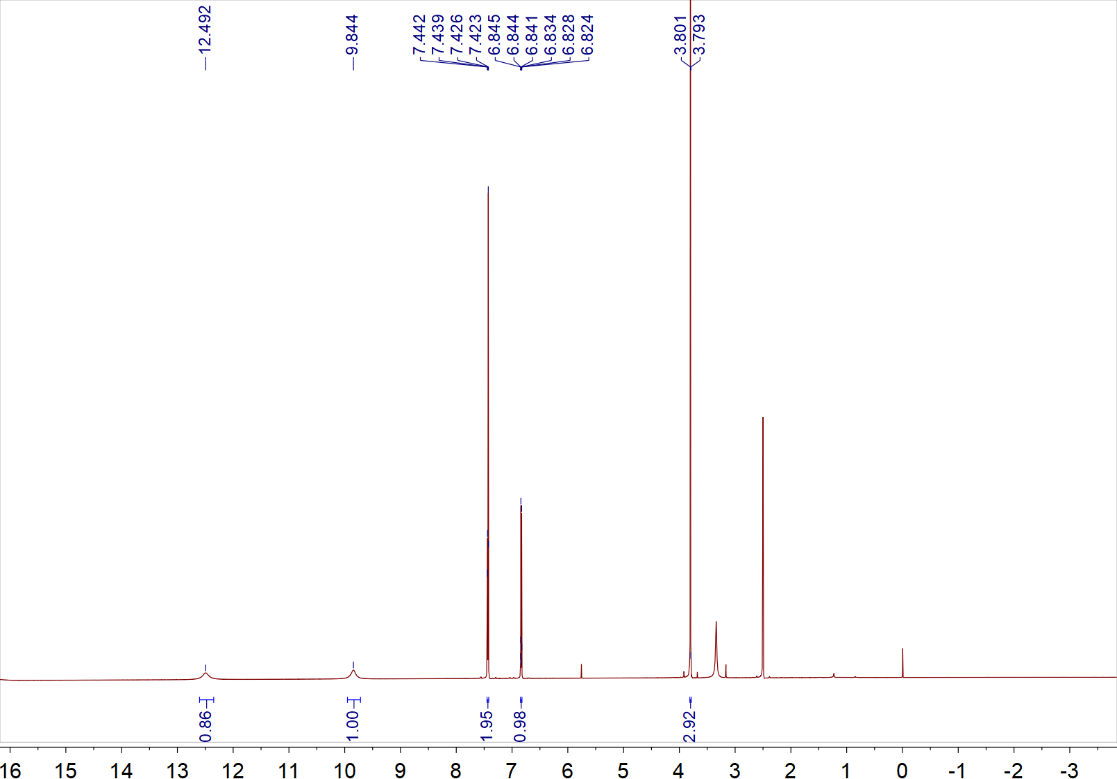


# Figure S100. ^1^H NMR spectrum of compound **37** in DMSO-*d*_6_

# Figure S101. ^13^C NMR spectrum of compound **37** in DMSO-*d*_6_

# Figure S102. ^1^H NMR spectrum of compound **38** in Methanol-*d*_4_

# Figure S103. ^13^C NMR spectrum of compound **38** in Methanol-*d*_4_

# Figure S104. ^1^H NMR spectrum of compound **39** in Methanol-*d*_4_

# Figure S105. ^1^H NMR spectrum of compound **40** in Methanol-*d*_4_

# Figure S106. ^13^C NMR spectrum of compound **40** in Methanol-*d*_4_

# Figure S107. ^1^H NMR spectrum of compound **41** in Methanol-*d*_4_

# Figure S108. ^13^C NMR spectrum of compound **41** in Methanol-*d*_4_

# Figure S109. ^1^H NMR spectrum of compound **42** in DMSO-*d*_6_

# Figure S110. ^13^C NMR spectrum of compound **42** in DMSO-*d*_6_

# Figure S111. ^1^H NMR spectrum of compound **43** in DMSO-*d*_6_

# Figure S112. ^13^C NMR spectrum of compound **43** in DMSO-*d*_6_

# Figure S113. ^1^H NMR spectrum of compound **45** in DMSO-*d*_6_

# Figure S114. ^13^C NMR spectrum of compound **45** in DMSO-*d*_6_

# Figure S115. ^1^H NMR spectrum of compound **48** in DMSO-*d*_6_

# Figure S116. ^13^C NMR spectrum of compound **48** in DMSO-*d*_6_

# Figure S117. ^1^H NMR spectrum of compound **49** in DMSO-*d*_6_

# Figure S118. ^13^C NMR spectrum of compound **49** in DMSO-*d*_6_

# Figure S119. ^1^H NMR spectrum of compound **50** in Methanol-*d*_4_

# Figure S120. ^13^C NMR spectrum of compound **50** in Methanol-*d*_4_

# Figure S121. GO enrichment analysis of the candidate targets

# Figure S122. KEGG pathway enrichment analysis of the candidate targets

# Figure S123. The correlation between PTP1B IC_50_ values and Stern-Volmer constants (K_sv_) of compounds **1**, **6**, **30**, **44** and **45**

# Table S1. Fifteen key targets of the fruits of Livistona chinensis against diabetes identified with network analyzer

| Uniprot ID | Gene name | Protein name | Degree |
| --- | --- | --- | --- |
| P29274 | ADORA2A | Adenosine receptor A2a | 17 |
| Q9NUW8 | TDP1 | Tyrosyl-DNA phosphodiesterase 1 | 15 |
| P30542 | ADORA1 | Adenosine receptor A1 | 14 |
| P31639 | SLC5A2 | Sodium/glucose cotransporter 2 | 13 |
| P23219 | PTGS1 | Prostaglandin G/H synthase 1 | 12 |
| P35354 | PTGS2 | Prostaglandin G/H synthase 2 | 12 |
| P10253 | GAA | Lysosomal alpha-glucosidase | 12 |
| P09917 | ALOX5 | Polyunsaturated fatty acid 5-lipoxygenase | 12 |
| Q16665 | HIF1A | Hypoxia-inducible factor 1-alpha | 12 |
| O75908 | SOAT2 | Sterol O-acyltransferase 2 | 12 |
| P00918 | CA2 | Carbonic anhydrase 2 | 11 |
| P43166 | CA7 | Carbonic anhydrase 7 | 11 |
| P35610 | SOAT1 | Sterol O-acyltransferase 1 | 11 |
| Q9ULX7 | CA14 | Carbonic anhydrase 14 | 11 |
| P18031 | PTPN1 | Tyrosine-protein phosphatase non-receptor type 1 | 11 |

# **UPLC-Q-TOF Parameters**

For the analysis, a 1290 series UPLC system coupled to a 6540 quadrupole TOF MS was used. The 6540 Q-TOF system was equipped with an Agilent JetStream ESI interface and was operated by Masshunter Workstation B.04.01 software. The general source settings in the positive (pos.) and negative (neg.) ionization modes were as follows: gas temperature, 350°C; gas flow, 8 L/min; reference nebulizer, 1 psi; sheath gas temperature, 380°C; sheath gas flow, 8 L/min; capillary voltage, 3500 V; nozzle voltage, 1500 V; capillary outlet voltage, 175 V; collision energy, 30 V; fragmentor voltage 75 V and reference mass, m/z 121.0509, 922.0117 (pos.), 112.9855, 966.0007 (neg.). The mass spectrometer was scanned from m/z 100 to 1700 in full scan mode.

# **The identification methodologies of known compounds**

(-)-epiafzelechin (**2**): white amorphous powder; ${[\alpha]}_{D}^{20}$ -32.8° (c 0.23, MeOH); ^1^H NMR data matched literature values [1]; the same Rf with the reference substance.

(+)-catechin (**3**): white amorphous powder; ${[\alpha]}_{D}^{20}$ +42.8° (c 0.23, MeOH); ^1^H and ^13^C NMR data were in agreement well with published data [2].

(-)-epicatechin (**4**): orange amorphous powder; ${[\alpha]}_{D}^{20}$ -45.7° (c 0.23, MeOH); ^1^H and ^13^C NMR data agreed well with literature values [3].

(-)-epiafzelechin-5-*O*-*β*-_D_-glucoside (**5**): orange amorphous powder; ${[\alpha]}_{D}^{20}$ -40.3° (c 0.21, MeOH); ^13^C NMR data matched literature values [4].

wogonin (**6**): yellow amorphous powder; HRESIMS m/z 285.0759 ([M+H]^+^, calculated for C_16_H_13_O_5_, 285.0763); ^1^H and ^13^C NMR data were in agreement well with published data [5].

genkwanin (**7**): yellow needle crystal; ^1^H and ^13^C NMR data agreed well with literature values [6].

tricin (**8**): yellow needle crystal; ^1^H data agreed well with reference values [7]; the same Rf with the reference substance.

vitexin (**9**): yellow needle crystal; ^1^H and ^13^C NMR data matched literature values [8]; the same Rf with the reference substance.

tricin-7-O-*β*-_D_-glucoside (**10**): yellow amorphous powder; ^1^H and ^13^C NMR data matched literature values [9].

isorhamnetin-3-*O*-*β*-_D_-glucoside (**11**): yellow amorphous powder; ^1^H and ^13^C NMR data agreed well with literature values [10].

astilbin (**12**): yellow amorphous powder; ^1^H and ^13^C NMR data matched literature values [40].

(7*S*,8*R*)-dihydrodehydrodiconiferyl alcohol (**13**): colorless colloidal solid; in CD spectrum, 221 nm: negative cotton, 239 nm: positive cotton; ^1^H and ^13^C NMR data agreed well with reference values [11].

(7*S*,8*R*)-5-methoxydihydrodehydroconiferyl alcohol (**14**): colorless colloidal solid; in CD spectrum, 221 nm: negative cotton, 239 nm: positive cotton; ^1^H and ^13^C NMR data matched literature values [12]

(7*S*,8*R*)-9,9′-dihydroxyl-3,3′-dimethoxyl-4-*O*-glycerol-7,8-dihydrobenzofuran-1′-propanolneoligan (**15**): colorless colloidal solid; in CD spectrum, 221 nm: negative cotton, 239 nm: positive cotton; ^1^H and ^13^C NMR data were in agreement well with published data [13].

(7*S*,8*R*)-dihydrodehydrodiconiferyl alcohol-4-*O*-*β*-_D_-glucopyranoside (**16**): colorless colloidal solid; in CD spectrum, 221 nm: negative cotton, 239 nm: positive cotton; ^1^H and ^13^C NMR data agreed well with reference values [14].

(7*S*,8*R*)-dihydrodehydrodiconiferyl alcohol-9'-*O*-*β*-_D_-glucoside (**17**): colorless colloidal solid; in CD spectrum, 221 nm: negative cotton, 239 nm: positive cotton; ^1^H and ^13^C NMR data were in agreement well with published data [15].

(7*S*,8*R*)-3,3′-dimethoxy-4-9-9′-trihydroxy-4′,7-epoxy-5,8′-lignan-4,9-bis-*O*-*β*-_D_-glucopyranoside (**18**): colorless colloidal solid; in CD spectrum, 221 nm: negative cotton, 239 nm: positive cotton; ^1^H and ^13^C NMR data were in agreement well with published data [16].

*threo*-(7*S*,8*S*)-guaiacyl-glycerol-*β*-*O*-4′-dihydroconiferyl ether (**19**): colorless colloidal solid; in CD spectrum, 230 nm: positive cotton; ^1^H and ^13^C NMR data agreed well with reference values [17].

*erythro*-(7*R*,8*S*)-guaiacyl-glycerol-*β*-*O*-4′-dihydroconiferyl ether (**20**): colorless colloidal solid; in CD spectrum, 240 nm: positive cotton; ^1^H and ^13^C NMR data were in agreement well with published data [18].

*erythro*-(7*R*,8*S*)-4,7,9,9′-tetrahydroxy-3,5,2′-trimethoxy-8-*O*-4′-neolignan (**21**): colorless colloidal solid; in CD spectrum, 240 nm: positive cotton; ^1^H and ^13^C NMR data agreed well with reference values [19].

*erythro*-(7*R*,8*S*)-4,7,9,9′-tetrahydroxy-3,3′-dimethoxy-8-*O*-4′-neolignan-9′-*O*-*β*-_D_-glucopyranoside (**22**): colorless colloidal solid; in CD spectrum, 240 nm: positive cotton; ^1^H and ^13^C NMR data were in agreement well with published data [20].

*threo*-(7*R*,8*R*)-4,7,9,9′-tetrahydroxy-3,3′-dimethoxy-8-*O*-4′-neolignan-9′-*O*-*β*-_D_-glucopyranoside (**23**): colorless colloidal solid; in CD spectrum, 240 nm: positive cotton; ^1^H and ^13^C NMR data agreed well with reference values [20].

*erythro*-(7*R*,8*S*)-7,9,9-trihydroxy-3,3,5-trimethoxy-8-*O*-4-neolignan-4-*O*-*β*-_D_-glucopyranoside (**24**): colorless colloidal solid; in CD spectrum, 240 nm: positive cotton; ^1^H and ^13^C NMR data were in agreement well with published data [21].

2-[4-(3-hydroxypropyl)-2-methoxyphenoxy]propane-1,3-diol (**25**): yellow amorphous powder; ^1^H and ^13^C NMR data agreed well with reference values [22].

2-[4-(3-hydroxy-propyl)-2,6-dimethoxyphenoxy]-propane-1,3-diol (**26**): yellow amorphous powder; ^1^H and ^13^C NMR data were in agreement well with published data [23].

salicifoliol (**27**): colorless colloidal solid; ^1^H and ^13^C NMR data agreed well with reference values [24].

4-ketopinoresinol (**28**): colorless colloidal solid; ^1^H and ^13^C NMR data agreed well with reference values [25].

(+)-pinoresinol (**29**): colorless colloidal solid; ${[\alpha]}_{D}^{20}$ +36.7° (c 0.20, CHCl_3_); ^1^H and ^13^C NMR data were in agreement well with published data [26].

(+)-medioresinol (**30**): colorless colloidal solid; ${[\alpha]}_{D}^{20}$ +43.1° (c 0.20, CHCl_3_); ^1^H and ^13^C NMR data agreed well with reference values [27].

(+)-syringaresinol (**31**): colorless colloidal solid; ${[\alpha]}_{D}^{20}$ +40.2°(c 0.20, CHCl_3_); ^1^H and ^13^C NMR data agreed well with reference values [28].

(7*R*,7′*R*,7′′*R*,8*S*,8′*S*,8′′*S*)-4′,4′′-dihydroxy-3,3′,3′′-triethoxy-7,9′:7′,9-diepoxy-4,8′′-oxy-8,8′-sesquineolignan-7′′,9′′-diol (**32**): colorless colloidal solid; in CD spectrum, 239 nm: positive cotton, 281 nm: positive cotton; ^1^H and ^13^C NMR data agreed well with reference values [28].

(7*S*,7′*S*,7′′*S*,8*R*,8′*R*,8′′*R*)-4′,4′′-dihydroxy-3,3′,3′′,5-tetramethoxy-7,9′:7′,9-diepoxy-4,8′′-oxy-8,8′-sesquineolignan-7′′,9′′-diol (**33**): colorless colloidal solid; in CD spectrum, 239 nm: positive cotton, 281 nm: positive cotton; ^1^H and ^13^C NMR data matched literature values [28].

(7*R*,7′*R*,7′′*R*,8*S*,8′*S*,8′′*S*)-4′,4′′-dihydroxy-3,3′,3′′,5-tetramethoxy-7,9′:7′,9-diepoxy-4,8′′-oxy-8,8′-sesquineolignan-7′′,9′′-diol (**34**): colorless colloidal solid; in CD spectrum, 239 nm: positive cotton, 281 nm: negative cotton; ^1^H and ^13^C NMR data agreed well with reference values [28].

(7*R*,7′*R*,7′′*R*,8*S*,8′*S*,8′′*S*)-4′,4′′-dihydroxy-3,3′,3′′,5,5′-pentamethoxy-7,9′:7′,9-diepoxy-4,8′′-oxy-8,8′-sesquineolignan-7′′,9′′-diol (**35**): colorless colloidal solid; in CD spectrum, 239 nm: positive cotton, 281 nm: negative cotton; ^1^H and ^13^C NMR data agreed well with reference values [29].

(7*S*,7′*S*,7′′*S*,8*R*,8′*R*,8′′*R*)-4′,4′′-dihydroxy-3,3′,3′′,5,5′,5′′-hexamethoxy-7,9′:7′,9-diepoxy-4,8′′-oxy-8,8′-sesquineolignan-7′′,9′′-diol (**36**): colorless colloidal solid; in CD spectrum, 239 nm: positive cotton, 281 nm: positive cotton; ^1^H and ^13^C NMR data were in agreement well with published data [27].

vanillic acid (**37**): white needle crystal; ^1^H and ^13^C NMR data matched literature values [30].

protocatechuic acid (**38**): white needle crystal; ^1^H and ^13^C NMR data were in agreement well with published data [31].

4-hydroxybenzoic acid (**39**): colorless needle crystal; ^1^H data agreed well with reference values [32]; the same Rf with the reference substance.

*p*-hydroxybenzaldehyde (**40**): colorless needle crystal; ^1^H data matched literature values [33]; the same Rf with the reference substance.

isovanillic acid (**41**): white needle crystal; ^1^H and ^13^C NMR data matched literature values [34].

protocatechuic acid methyl ester (**42**): colorless flake crystal; ^1^H and ^13^C NMR data matched literature values [35].

3,4-dimethoxybenzyl alcohol (**43**): light yellow colloidal solid; ^1^H and ^13^C NMR data were in agreement well with published data [36].

variecolorquinones A (**44**): brown solid; HRESIMS m/z 401.0878 ([M-H]^-^, calculated for C_20_H_17_O_9_, 401.0873); ^1^H and ^13^C NMR data were in agreement well with published data [37].

physcion (**45**): yellow needle crystal; ^1^H and ^13^C NMR data were in agreement well with published data [38].

1*H*-indole-3-carbaldehyde (**48**): colorless needle crystal; ^1^H and ^13^C NMR data matched literature values [39].

uridine (**49**): white amorphous powder; ^1^H and ^13^C NMR data agreed well with reference values [40].

aurantiamide acetate (**50**): white amorphous powder; ^1^H and ^13^C NMR data were in agreement well with published data [41].

**Reference**

[1] B. Yang, Z. Fan, J.-P. Zhu, T. Yang, G.-T. Peng, Q.-l. TAN, Z.-X. Zhao, Chemical constituents from Phymatopteris hastate, Zhong Cao Yao 2014 (2014) 21.

[2] S.A. Khalid, S. Yagi, P. Khristova, H. Duddeck, (+)-Catechin-5-galloyl ester as a novel natural polyphenol from the bark of Acacia nilotica of Sudanese origin1, Planta Med. 55(06) (1989) 556-558.

[3] Z. Zhou, C. Yang, Chemical constituents of crude green Pu-Er tea in Yunnan, Acta Botanica Yunnanica 22(3) (2000) 343-350.

[4] V. Sethi, S. Taneja, K. Dhar, C. Atal, (−)-Epiafzelechin 5-O-βD-glucoside from crataeva religiosa, Phytochemistry 23(10) (1984) 2402-2403.

[5] X.Q. Zhou, H. Liang, X.H. Lu, S.Q. Cai, B. Wang, Y.Y. Zhao, Flavonoids from Scutellaria baicalensis and their bioactivities, J. Peking Univ. Health Sci. 41(5) (2009) 578-584.

[6] S. Li, Chemical constituents of Gendarussa vulgaris, China. Tradit. Zhong Cao Yao 49 (2018) 3998-4002.

[7] Y. Hu, J. Zhang, D. Liu, J. Guo, T. Liu, Z. Xin, Pencitrin and pencitrinol, two new citrinin derivatives from an endophytic fungus Penicillium citrinum salicorn 46, Phytochem. Lett. 22 (2017) 229-234.

[8] F. Yin, L. Hu, R. Pan, Novel dammarane-type glycosides from Gynostemma pentaphyllum, Chem. Pharm. Bull. 52(12) (2004) 1440-1444.

[9] X.-L. Lu, Y. Qiao, X.-M. Zhang, B.-L. Ma, M.-H. Qiu, Chemical constituents from Ceratophyllum demersum (Ceratophyllaceae), Yunnan Zhi Wu Yan Jiu 29(2) (2007) 263-264.

[10] M. Wolbiś, M. Królikowska, Flavonol glycosides from Sedum acre, Phytochemistry 27(12) (1988) 3941-3943.

[11] R. Kasai, S. Hirono, W.-H. Chou, O. TANAKA, F.-H. Chen, Sweet dihydroflavonol rhamnoside from leaves of Engelhardtia chrysolepis, a Chinese folk medicine, Hung-qi, Chem. Pharm. Bull. 36(10) (1988) 4167-4170.

[12] C.H. Park, K.H. Kim, I.K. Lee, S.Y. Lee, S.U. Choi, J.H. Lee, K.R. Lee, Phenolic constituents of Acorus gramineus, Arch. Pharm. Res. 34(8) (2011) 1289-1296.

[13] X.-X. Hyang, D.-M. Li, L.-Z. Li, D.-D. Guo, R.-T. Ren, S.-J. Song, Isolation and identification of chemical constituents from leaves of Crataegus pinnatifida Bge, J. Shenyang Pharm. Univ. 5 (2012).

[14] W.-J. Xiang, L. Ma, L.-H. Hu, Neolignans and flavonoids from the root bark of Illicium henryi, Fitoterapia 81(8) (2010) 1228-1231.

[15] X.-C. Zhao, J.-L. Du, Y.-G. Xie, Y. Zhang, H.-Z. Jin, Chemical Constituents of the Flowers of Hemerocallis minor, Chem. Nat. Compd. 54(3) (2018) 556-558.

[16] Y. Takeda, C. Mima, T. Masuda, E. Hirata, A. Takushi, H. Otsuka, Glochidioboside, a glucoside of (7S, 8R)-dihydrodehydrodiconiferyl alcohol from leaves of Glochidion obovatum, Phytochemistry 49(7) (1998) 2137-2139.

[17] D.-Q. Dou, X.-Y. Hu, Y.-R. Zhao, T.-G. Kang, F.-Y. Liu, H.-X. Kuang, D.C. Smith, Studies on the anti-psoriasis constituents of Oplopanax elatus Nakai, Nat. Prod. Res. 23(4) (2009) 334-342.

[18] X.-X. Huang, C.-C. Zhou, L.-Z. Li, F.-F. Li, L.-L. Lou, D.-M. Li, T. Ikejima, Y. Peng, S.-J. Song, The cytotoxicity of 8-O-4′ neolignans from the seeds of Crataegus pinnatifida, Bioorganic Med. Chem. Lett. 23(20) (2013) 5599-5604.

[19] Y. Zhang, Y.-B. Liu, Y. Li, S.-G. Ma, L. Li, J. Qu, D. Zhang, J.-D. Jiang, S.-S. Yu, Phenolic constituents from the roots of Alangium chinense, Chin. Chem. Lett. 28(1) (2017) 32-36.

[20] M. Gan, Y. Zhang, S. Lin, M. Liu, W. Song, J. Zi, Y. Yang, X. Fan, J. Shi, J. Hu, Glycosides from the root of Iodes cirrhosa, J. Nat. Prod. 71(4) (2008) 647-654.

[21] C. Huo, H. Liang, B. Wang, Y. Zhao, New neolignan glycosides and a new cerebroside from Symplocos caudata, Chem. Nat. Compd. 46(3) (2010) 343-347.

[22] L. Zhao, L.-Z. Li, Y. Peng, S.-J. Song, Isolation and identification of chemical constituents from seeds of Crataegus pinnatifida Bge, J. Shenyang Pharm. Univ. 1 (2012).

[23] J. Chen, X.-Q. Xu, X.-D. Kang, W.-T. Zhang, X.-H. Huang, S.-H. Li, C.-C. Zhao, Three New Phenolic Compounds from Eucommia ulmoides, Chem. Nat. Compd. 53(2) (2017) 254-256.

[24] Q. Liu, J. Li, X. Chai, Y. Jiang, P. Tu, Chemical constituents from Qianliang tea, J. Chinese Pharm. Sci. 22(5) (2013) 427-430.

[25] C.S. Na, S.S. Hong, Y.-H. Choi, Y.H. Lee, S.H. Hong, J.-Y. Lim, B.H. Kang, S.-Y. Park, D. Lee, Neuroprotective effects of constituents of Eragrostis ferruginea against Aβ-induced toxicity in PC12 cells, Arch. Pharm. Res. 33(7) (2010) 999-1003.

[26] T. Deyama, The constituents of Eucommia ulmoides Oliv. I. Isolation of (+)-medioresinol di-O-β-D-glucopyranoside, Chem. Pharm. Bull. 31(9) (1983) 2993-2997.

[27] H. TSUKAMOTO, S. HISADA, S. NISHIBE, Lignans from bark of Fraxinus mandshurica var. japonica and F. japonica, Chem. Pharm. Bull. 32(11) (1984) 4482-4489.

[28] L. Xiong, C. Zhu, Y. Li, Y. Tian, S. Lin, S. Yuan, J. Hu, Q. Hou, N. Chen, Y. Yang, Lignans and neolignans from Sinocalamus affinis and their absolute configurations, J. Nat. Prod. 74(5) (2011) 1188-1200.

[29] J.-L. Li, N. Li, H.-S. Lee, S.-S. Xing, S.-Z. Qi, Z.-D. Tuo, L. Zhang, B.-B. Li, J.-G. Chen, L. Cui, Four new sesqui-lignans isolated from Acanthopanax senticosus and their diacylglycerol acyltransferase (DGAT) inhibitory activity, Fitoterapia 109 (2016) 185-189.

[30] D. Shataer, R. Abdulla, Q. Ma, G. Liu, H. Aisa, Chemical Composition of Extract of Corylus avellana Shells, Chem. Nat. Compd. 56(2) (2020) 338-340.

[31] W. Wei, Y. Chong-Ren, Z. Ying-Jun, Phenolic Constituents from the Fruits of Amomum tsao-ko (Zingiberaceae), Plant Divers. 31(03) (2009) 284.

[32] D. Wang, X. Wu, D. Zhang, B. Zhu, Q. Jia, Y. Li, Chemical constituents of carboxylic acid and its derivatives in Cinnamomi Ramulus, Zhong Cao Yao 50 (2019) 8-12.

[33] N. Bouaicha, P. Amade, D. Puel, C. Roussakis, Zarzissine, a new cytotoxic guanidine alkaloid from the Mediterranean sponge Anchinoe paupertas, J. Nat. Prod. 57(10) (1994) 1455-1457.

[34] H. HU, Y.-X. WU, X.-J. HE, Chemical constituents of radix Marsdeniae sinensis, J. Med. Chem. (2010) 06.

[35] J. Zhang, Y. Kuang, L. Liu, S. Yang, L. Zhao, Chemical constituents from root tubers of Fagopyrum dibotrys, Zhong Cao Yao 47(5) (2016) 722-726.

[36] S. McCloskey, S. Noppawan, W. Mongkolthanaruk, N. Suwannasai, T. Senawong, U. Prawat, A new cerebroside and the cytotoxic constituents isolated from Xylaria allantoidea SWUF76, Nat. Prod. Res. 31(12) (2017) 1422-1430.

[37] G. Li, X. Li, L. Cao, L. Zhang, L. Shen, J. Zhu, J. Wang, J. Si, Sesquiterpene coumarins from seeds of Ferula sinkiangensis, Fitoterapia 103 (2015) 222-226.

[38] O. Smetanina, A. Kalinovskii, Y.V. Khudyakova, N. Slinkina, M. Pivkin, T. Kuznetsova, Metabolites from the marine fungus Eurotium repens, Chem. Nat. Compd. 43(4) (2007) 395-398.

[39] C. Wang, Y. Yang, Z. Mei, X. Yang, Cytotoxic compounds from Laminaria japonica, Chem. Nat. Compd. 49(4) (2013) 699-701.

[40] H. Liang, Y.-J. Bai, Y.-Y. Zhao, R.-Y. Zhang, The Chemical Constituents from the Roots of Bupleurum chinense DC, J. Chinese Pharm. Sci. 7(2) (1998) 98.

[41] Q.-X. Mei, X.-l. Chen, X. Xia, Z.-J. Fang, H.-B. Zhou, Y.-Q. Gao, W.-B. Dai, R.-W. Jiang, Isolation and chemotaxonomic significance of chemical constituents from Rubus parvifolius, Zhong Cao Yao 8(1) (2016) 75-79.
